# Differences in the reporting of conflicts of interest and sponsorships in systematic reviews with meta-analyses in dentistry: an examination of factors associated with their reporting

**DOI:** 10.1186/s41073-024-00150-y

**Published:** 2024-09-30

**Authors:** Jonas Heymann, Naichuan Su, Clovis Mariano Faggion

**Affiliations:** 1 Department of Periodontology and Operative Dentistry, University Hospital Münster, Waldeyerstraße 30, 48149 Münster, Germany; 2 Department of Oral Public Health, Academic Centre for Dentistry Amsterdam (ACTA), University of Amsterdam and Vrije Universiteit Amsterdam, 1081 LA Amsterdam, The Netherlands

**Keywords:** Conflict of interest, Methods, Systematic reviews, Abstract, Ethics in publishing

## Abstract

**Background:**

Reporting conflicts of interest (COI) and sources of sponsorship are of paramount importance in adequately interpreting the results of systematic reviews. Some evidence suggests that there is an influence of COI and sponsorship on the study results.

The objectives of this meta-research study were twofold: (a) to assess the reporting of COI and sponsorship statements in systematic reviews published in dentistry in three sources (abstract, journal’s website and article’s full text) and (b) to assess the associations between the characteristics of the systematic reviews and reporting of COI.

**Methods:**

We searched the PubMed database for dental systematic reviews published from database inception to June 2023. We assessed how COI and sponsorship statements were reported in the three sources. We performed a logistic regression analysis to assess the associations between the characteristics of the systematic reviews and the reporting of COI.

**Results:**

We assessed 924 abstracts published in PubMed and on the corresponding journals´ websites. Similarly, full texts associated with the 924 abstracts were also assessed. A total of 639 (69%) and 795 (88%) studies had no statement of COI in the abstracts on PubMed and the journal’s website, respectively. In contrast, a COI statement was reported in 801 (87%) full texts. Sponsorship statements were not reported in 911 (99%) and 847 (93%) abstracts published in PubMed and a journal´s website, respectively. Nearly two-thirds of the full-text articles (*N* = 607) included sponsorship statements. Journal access was significantly associated with COI statement reporting in all three sources. Open-access journals have significantly higher odds to report COI in PubMed and full-texts, while have significantly lower odds to report COI in the websites, compared with subscription or hybrid journals. Abstract type was significantly associated with COI statement reporting on the journal’s website and in the full text. Review registration based on the full text and the number of authors were significantly associated with COI statement reporting in PubMed and in the full texts. Several other variables were found to be significantly associated with COI statement reporting in one of the three sources.

**Conclusions:**

COI and sponsorship statements seem to be underreported in the abstracts and homepage of the journals, compared to the full-texts. These results were particularly more pronounced in abstracts published in both the PubMed database and the journals’ websites. Several characteristics of systematic reviews were associated with COI statement reporting.

**Supplementary Information:**

The online version contains supplementary material available at 10.1186/s41073-024-00150-y.

## Introduction

Systematic reviews are an important source of information to answer clinical questions [1], inform the prevalence of a specific disease or condition [2] and also serve as a basis for the development of clinical guidelines [3].

An important part of an systematic review is the abstract that summarises the most important information reported in the full text. Some published data suggest that many clinicians may access only the abstract of a scientific article, mainly due to a lack of time for reading due to their various duties [4]. Hence, an abstract should contain all the information that could be important for the reader to interpret potential biases that could interfere with the study results. In the biomedical field, abstracts are usually published in major databases, such as PubMed, and on the websites of the journals publishing the article.

Potential financial conflicts of interest (COI) may be a source of bias in studies. For example, financial COI was reported to be associated with more positive results in articles published in two major medical journals [5]; in the review, after controlling for sample size, study design and country of primary authors, studies with COIs had 2.35 (95% confidence interval [CI] 1.08–5.09) times higher odds of reporting positive results than those without COIs among all treatment studies from *The New English Journal of Medicine* and *The Journal of the America Medical Association*. Evidence also suggests that there is a positive association between COI and the report of positive results in dental randomised clinical trials (RCTs) [6]; in the review, the RCTs with COIs had 2.40–9.19 times higher odds of reporting positive results depending on the definition of COI. There has also been a reported association between financial and non-financial COIs and favourable recommendations in other types of scientific publications, such as clinical guidelines, opinion articles and narrative reviews [7].

Sponsorship has also been associated with more favourable efficacy results and conclusions in trials of devices and drugs that are sponsored by their manufacturers [8]; in this systematic review of 25 papers, studies that were sponsored by industry had a 1.27 times higher risk of reporting favourable efficacy results. Reporting sponsorship should also be a requirement for abstracts in order to allow an adequate interpretation of findings. There is a substantial amount of literature assessing the impact of COIs/sponsorship on systematic reviews [9–14].

Therefore, the objectives of this study were twofold: (a) to assess how COI and sponsorship statements are reported in systematic reviews in three sources (the abstract, journal’s website and article full text) and (b) to assess the associations between the characteristics of the systematic reviews and reporting of COI.

## Materials and methods

### Eligibility criteria

In the current research, we included systematic reviews with meta-analyses in the dental field that were published in English and included in PubMed. All types of systematic reviews with meta-analyses were included, and there was no restriction on species. We included only reviews with meta-analyses because we hypothesised that estimates from meta-analyses might be more relevant in the decision-making process. Therefore, one can argue that these meta-analyses are more sensitive to COIs and sponsorship. Systematic reviews without meta-analyses, published in languages other than English or outside of the dental field were excluded.

### Search strategy, data selection and rationale

On 16 June 2023, we searched for dental systematic reviews published in the PubMed database using a pre-defined search strategy (see supplementary file). The search included articles published from database inception to June 2023. Duplicates were removed, and the articles that remained had their abstracts assessed for the reporting of COI.

The abstracts available in PubMed were then assessed to identify the reporting of statements on COI. We chose PubMed to check the information on COIs for two main reasons: (1) the abstracts in PubMed are widely and freely available to anyone, and (2) PubMed is the most well-known biomedical database; many clinicians and interested readers likely use it as a reference to assess abstracts of indexed articles. After checking the abstracts reported in PubMed and the journals’ websites, we scrutinised the full text associated with each abstract for information on included statements reporting potential COIs. The research procedures in the use of PubMed, the journals’ websites and full text articles were conducted independently and in duplicate by two authors (JH, CMF) with a sample of 10% of the included abstracts/articles until an agreement of at least 80% was reached among the assessors. One assessor (JH) then conducted the procedures with the remaining sample (90%).

### Data extraction

The following data were directly extracted into a standardised Excel form: (1) reporting of COI statement (YES/NO); (2) reporting of sponsorship (YES/NO); (3) type of COI reported (financial or non-financial); (4) type of systematic review (interventional or non-interventional); (5) type of primary studies in the systematic review (in-vitro or animals, humans or both); (6) journal type (dentistry or other); (7) systematic review registration (YES/NO); (8) continent of origin (North America, South America, Europe, Asia, Africa or Oceania); (9) country of origin (developing and developed); (10) abstract type (structured or non-structured); (11) journal impact factor (IF) (JCR 2022); (12) number of citations in Google Scholar; (13) journal access (subscription/hybrid or open access); (14) journal reporting a COI policy in the instructions to authors (YES/NO); (15) journal reporting the type of COI in the instructions to authors (financial, non-financial, both, only say that authors need to report COI, or no information); and (16) number of authors. Two assessors (JH, CMF) independently extracted a sample of articles until they reached at least 80% agreement [15]. One assessor (JH) then extracted the remaining data.

### Statistical analysis

Descriptive statistics were used to summarise the characteristics of the included systematic reviews. Proportions were used for categorical variables, and median and interquartile range (IQR) were used for continuous variables because all the three continuous variables (i.e. journal impact factor, number of citations, and number of authors) were not normally distributed based on Kolmogorov–Smirnov tests. To compare the prevalence of the reporting of COI and sponsorship among the three sources (PubMed, website and full text), Cochran’s Q tests were used. To assess the associations between the characteristics of the systematic reviews (independent variables) and the reporting of COI on the three sources separately, binary logistic regression analysis was performed. First, univariate binary logistic regression analysis was performed to assess the association of each independent variable with the reporting of COI separately. Second, the multicollinearity of the independent variables that were significant in the univariate analyses (*P* < 0.05) were tested using the variance inflation factor (VIF) before they were included in the subsequent multivariate binary logistic regression analysis. When a VIF value of a variable was > 5, collinearity was considered present, and the variable was excluded from the following analysis [16]. Third, a multivariate binary logistic regression analysis with backward selection was performed to further assess the association between the independent variables and the reporting of COIs.

## Results

### Selection of abstracts/full-text

**Fig. 1:**
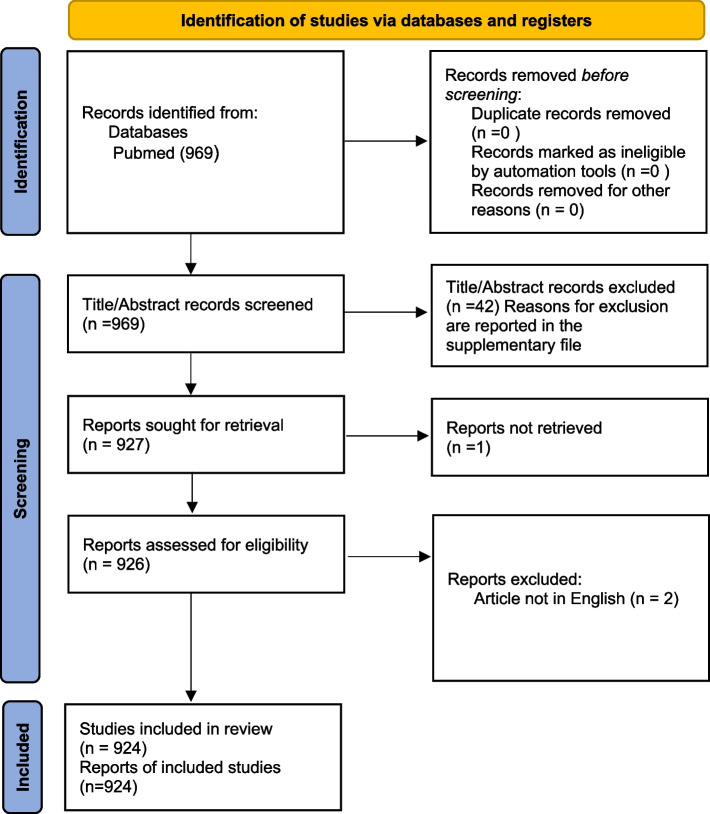
Flow of the selection process

A total of 969 abstracts were initially retrieved from the PubMed database. After the title and abstract assessment, 42 abstracts (4%) were excluded because they did not meet the eligibility criteria. After the full-text analysis, three other articles were excluded. Therefore, 924 abstracts with their respective full texts were finally included. Figure 1 reports the selection of the abstracts/articles.

### Descriptive results

The most prevalent country of affiliation of the first author was Brazil (*n* = 185, 20%), followed by China (*n* = 88, 10%) and the USA (*n* = 86, 9%). Most of the studies (*n* = 524, 57% of the whole sample) were published between 2020 and 2023. A large range of dentistry-associated journals, for example, *Clinical Oral Investigations* (*n* = 44, 5%), *BMC Oral Health* (*n* = 22, 2%), *Journal of Evidence-Based Dental Practice* (*n* = 10, 1%) and others (in total: *n* = 762; 82%), were identified. About two-thirds (*n* = 622, 67%) of the journals were either accessible via subscription or had a hybrid system. Articles accessible via open access comprised 33% (*n* = 302) of the present sample. In addition, 758 of the 924 (82%) reviews analysed were based on interventional studies, and 816 (88%) reviews included studies on human subjects. The most observed dental discipline was implant dentistry, with a total of 352 (38%) articles. The journal impact factor ranged from 0.2 to 18.6 (median = 3.4) and the median of number of citations of the included articles was 22.0. A total of 849 (92%) reviews have been published in a journal with an impact factor.

The characteristics of the assessed abstracts/articles are reported in detail in Table 1.

**Table 1:** Characteristics of the systematic reviews included

Characteristics	Frequency(%)
**Country of First Author**	
Brazil	185(20.02)
China	88(9.52)
USA	86(9.31)
Italy	55(5.95)
Spain	54(5.84)
India	52(5.63)
Germany	36(3.90)
Iran	36(3.90)
Switzerland	35(3.79)
United Kingdom	26(2.81)
Sweden	24(2.60)
Australia	22(2.38)
Netherlands	15(1.62)
Saudi Arabia	14(1.52)
Denmark	13(1.40)
Egypt	11(1.19)
Malaysia	11(1.19)
Portugal	10(1.08)
Canada	10(1.08)
Others	141(15.26)
**Year of Publication**	
2023	107(11.58)
2022	161(17.42)
2021	151(16.34)
2020	105(11.36)
2019	81(8.77)
2018	102(11.04)
2017	54(5.84)
2016	69(7.47)
2015	30(3.25)
2014	25(2.71)
2013	12(1.30)
2012	7(0.76)
2011	5(0.54)
2010	6(0.65)
2009	3(0.32)
2005	1(0.11)
2002	3(0.32)
2001	1(0.11)
1997	1(0.11)
**Review Type**	
Interventional reviews	758(82.03)
Observational reviews	166(17.97)
**Abstract Type**	
Structured	668(72.29)
Non-Structured	256(27.71)
**Journal**	
Clinical Oral Investigations	44(4.76)
Clinical Oral Implants Research	43(4.65)
International Journal of Oral and Maxillofacial Surgery	38(4.11)
Journal of Dentistry	36(3.90)
Journal of Prosthetic Dentistry	28(3.03)
The International Journal of Oral & Maxillofacial Implants	25(2.70)
The Journal of the American Dental Association	23(2.49)
BMC Oral Health	22(2.38)
Journal of Periodontology	21(2.27)
Clinical Implant Dentistry & Related Research	21(2.27)
Journal of Dental Research	20(2.16)
Journal of Clinical Periodontology	20(2.16)
Journal of Endodontics	16(1.73)
Journal of Oral Rehabilitation	15(1.62)
Journal of Clinical Medicine	14(1.52)
Journal of Oral & Maxillofacial Research	11(1.19)
PLOS One	11(1.19)
European Journal of Orthodontics	11(1.19)
International Endodontic Journal	10(1.08)
BMJ Open	10(1.08)
Journal of Evidence-Based Dental Practice	10(1.08)
Other	474(51.30)
**Journal Type**	
Dentistry	762(82.47)
Other Field than Dentistry	162(17.53)
**Journal Access**	
Subscription/ Hybrid	622(67.32)
Open Access	302(32.68)
**Dental Discipline**	
Implant Dentistry	352(38.10)
Orthodontics	95(10.28)
Endodontics	77(8.33)
Oral Surgery	63(6.82)
Pediatric Dentistry	44(4.76)
Periodontics	40(4.33)
Oral Pathology	18(1.95)
Restorative Dentistry	18(1.95)
Diagnostics	16(1.73)
Prosthetic Dentistry	14(1.52)
Oral Health	11(1.19)
General Dentistry/Others	176(19.05)
**Review Registration (Statement Full Text)**	
Yes	507(54.87)
No	417(45.13)
**Primary Studies included in Reviews (Full Text)**	
In-vitro and/or Animal Studies	77(8.33)
Human Studies	816(88.31)
Both	31(3.35)
**COI Statement reported in PubMed**	
Yes	285(30.84)
No	639(69.16)
**COI Statement reported on the Website of the Journal**	
Yes	112(12.35)
No	795(87.65)
**COI Statement reported in the Full Text Article**	
Yes	801(86.69)
No	123(13.31)
**Sponsorship Statement reported in PubMed**	
Yes	13(1.41)
No	911(98.59)
**Sponsorship Statement reported on the Website of the Journal**	
Yes	60(6.62)
No	847(93.38)
**Sponsorship Statement reported in the Full Text Article**	
Yes	607(65.76)
No	316(34.24)
**Article Characteristics**	**Median (Interquartile range)**
Journal Impact Factor	3.4 (2.4-4.3)
Number of Citations	22.0 (7.0-59.0)
Number of Authors	5.0 (4.0-6.0)

### Reporting of COI statement and sponsorship

A high percentage of the abstracts (PubMed = 69%, journals’ websites = 88%) did not include a statement about COIs. When a COI statement was reported, “no COI” was the most frequently mentioned COI type in all three examined sources (PubMed: *n* = 283, 99%; journals’ websites: *n* = 109, 97%; full text: *n* = 780, 97%). In the full texts (*n* = 13, 2%), the second most declared COI type after “no COI” mentioned by at least one of the authors of the respective review was a financial COI. In contrast, a statement regarding COI was frequently observed in the full texts (*n* = 801, 87%). Overall, the prevalence of COI statement reporting differed significantly among the three sources (*P* < 0.01) (Table 2). Similarities were observed regarding sponsorship statements in the abstracts, where an even higher proportion of no sponsorship statement was observed (PubMed = 99%, journals’ websites = 93%). In comparison, nearly two-thirds of the full-text articles included a sponsorship statement (66%). The prevalence of sponsorship statement reporting in the full texts was significantly higher than that in the PubMed abstracts (*P* < 0.01) and journal website abstracts (*P* < 0.01). However, no significant difference was found in sponsorship statement reporting between the abstracts on PubMed and the journals’ websites (*P* = 0.05) (Table 3).

**Table 2:** Characteristics of the independent variables based on COI reporting at different sources

		PubMed		Website^a^		Full text	
Variables	Total (*N* = 924)	Yes (*N* = 285)	No (i = 639)	Yes (*N* = 112)	No (*N* = 795)	Yes (*N* = 801)	No (*N* = 123)
**Type of primary studies based on the full-text**							
In-vitro or animals	77 (8%)	44 (57%)	33 (43%)	10 (13%)	67 (87%)	67 (87%)	10 (13%)
Humans	816 (88%)	226 (28%)	590 (72%)	100 (13%)	699 (88%)	704 (86%)	112 (14%)
Both	31 (3%)	15 (48%)	16 (52%)	2 (7%)	29 (94%)	30 (97%)	1 (3%)
**Review type**							
Intervention	758 (82%)	224 (30%)	534 (70%)	88 (12%)	654 (88%)	655 (86%)	103 (14%)
Non-intervention	166 (18%)	61 (37%)	105 (63%)	24 (15%)	141 (85%)	146 (88%)	20 (12%)
**Review registration based on full-text**							
Yes	506 (55%)	181 (36%)	325 (64%)	68 (14%)	432 (86%)	453 (90%)	53 (10%)
No	417 (45%)	104 (25%)	313 (75%)	44 (11%)	362 (89%)	347 (83%)	70 (17%)
**Continents of origin of the reviews**							
North America	101 (11%)	20 (20%)	81 (80%)	13 (13%)	85 (87%)	89 (88%)	12 (12%)
South America	196 (21%)	45 (23%)	151 (77%)	21 (11%)	172 (89%)	163 (83%)	33 (17%)
Europe	314 (34%)	108 (34%)	206 (66%)	32 (10%)	275 (90%)	270 (86%)	44 (14%)
Asia	268 (29%)	102 (38%)	166 (62%)	42 (16%)	222 (84%)	246 (92%)	22 (8%)
Africa	14 (2%)	5 (36%)	9 (64%)	0 (0%)	14 (100%)	11 (79%)	3 (21%)
Oceania	31 (3%)	5 (16%)	26 (84%)	4 (13%)	27 (87%)	22 (71%)	9 (29%)
**Countries of origin of the reviews**							
Developing	480 (52%)	151 (32%)	329 (68%)	62 (13%)	411 (87%)	422 (88%)	58 (12%)
Developed	444 (48%)	134 (30%)	310 (70%)	50 (12%)	384 (89%)	379 (85%)	65 (15%)
**Abstract type**							
Structured	668 (72%)	187 (28%)	481 (72%)	95 (14%)	563 (86%)	567 (85%)	101 (15%)
Non-structured	256 (28%)	98 (38%)	158 (62%)	17 (7%)	232 (93%)	234 (91%)	22 (9%)
**Review access based on homepage**							
Yes	611 (66%)	263 (43%)	348 (57%)	59 (10%)	537 (90%)	546 (89%)	65 (11%)
No	313 (34%)	22 (7%)	291 (93%)	53 (17%)	258 (83%)	255 (82%)	58 (18%)
**Review access based on internet**							
Yes	864 (94%)	283 (33%)	581 (67%)	104 (12%)	744 (88%)	747 (87%)	117 (13%)
No	60 (6%)	2 (3%)	58 (97%)	8 (14%)	51 (86%)	54 (90%)	6 (10%)
**Journal type**							
Dentistry	762 (82%)	177 (23%)	585 (77%)	97 (13%)	653 (87%)	651 (85%)	111 (15%)
Other	162 (18%)	108 (67%)	54 (33%)	15 (10%)	142 (90%)	150 (93%)	12 (7%)
**Journal access**							
Subscription or hybrid	622 (67%)	67 (11%)	555 (89%)	99 (16%)	518 (84%)	515 (83%)	107 (17%)
Open access	302 (33%)	218 (72%)	84 (28%)	13 (5%)	277 (95%)	286 (95%)	16 (5%)
**Journal reporting a COI policy in the instructions to authors**							
Yes	900 (97%)	272 (30%)	628 (70%)	110 (12%)	776 (88%)	783 (87%)	117 (13%)
No	24 (3%)	13 (54%)	11 (46%)	2 (10%)	19 (90%)	18 (75%)	6 (25%)
**Journal reporting the type of COI in the instructions to authors**							
Financial	88 (10%)	25 (28%)	63 (72%)	13 (16%)	71 (84%)	72 (82%)	16 (18%)
Non-financial	2 (0%)	1 (50%)	1 (50%)	0 (0%)	2 (100%)	2 (100%)	0 (0%)
Both	761 (82%)	236 (31%)	525 (69%)	94 (13%)	657 (87%)	666 (88%)	95 (12%)
Only say that authors need to report COI	49 (5%)	10 (20%)	39 (80%)	3 (6%)	46 (94%)	43 (88%)	6 (12%)
No information	24 (3%)	13 (54%)	11 (46%)	2 (8%)	22 (92%)	18 (75%)	6 (25%)
**Journal impact factor**^b,d^	3.4 (2.4-4.3)	3.4 (2.6-4.3)	3.4 (2.4-4.3)	3.6 (2.9-4.0)	3.4 (2.4-4.3)	3.4 (2.4-4.3)	4.3 (2.6-4.6)
**Number of citations**^c,d^	22.0 (7.0-59.0)	10.0 (4.0-26.0)	31.0 (10.0-76.0)	23.0 (6.0-47.0)	22.0 (7.0-62.0)	20.0 (6.0-54.8)	42.0 (14.3-93.8)
**Number of authors**^d^	5.0 (4.0-6.0)	6.0 (4.0-7.0)	5.0 (4.0-6.0)	5.0 (4.0-6.0)	5.0 (4.0-6.0)	5.0 (4.0-6.0)	4.0 (3.0-6.0)

^a^At the homepage level, 17 studies were not applicable and therefore were not included in the analysis

^b^: 75 studies were excluded because of no impact factors in the journals where the studies were published

^c^: 44 studies were excluded because number of citations were not applicable

^d^: median and interquartile range were presented

**Table 3:** Comparison of COI reporting between different sources

**COI reporting**				
	PubMed (*N* = 924)	Website (*N* = 907)	Full-text (*N* = 924)	*P* value
Yes	285 (31%)	112 (12%)	801 (87%)	< 0.01* (overall) PubMed vs. Home page: < 0.01* PubMed vs. Full-text: < 0.01* Home page vs. Full text: < 0.01*
No	639 (69%)	795 (88%)	123 (13%)	
**Sponsorship reporting**				
	PubMed level (*N* = 924)	Website (*N* = 907)	Full-text level (*N* = 923)	*P* value
Yes	13 (1%)	60 (7%)	607 (66%)	< 0.01*(overall) PubMed vs. Home page: 0.05 PubMed vs. Full-text: < 0.01* Home page vs. Full text: < 0.01*
No	911 (99%)	847 (93%)	316 (34%)	
**COI types**				
	PubMed level (*N* = 285)	Website (*N* = 112)	Full-text level (*N* = 801)	*P* value
Financial	0 (0%)	2 (2%)	13 (2%)	NA
Non-financial	2 (1%)	0 (0%)	2 (0%)	
Both	0 (0%)	1 (1%)	6 (1%)	
No COI	283 (99%)	109 (97%)	780 (97%)	
**COI details**				
	PubMed level (*N* = 39)	Website (*N* = 15)	Full-text level (*N* = 134)	*P* value
Only financial	19 (49%)	11 (73%)	73 (55%)	NA
Only non-financial	3 (8%)	0 (0%)	4 (3%)	
Both	17 (44%)	4 (27%)	57 (43%)	

### Regression analyses

Table 4 shows the association between the different characteristics of the reviews and the reporting of COI in the PubMed abstracts. Based on the univariate analysis, the COI reporting in PubMed was significantly associated with the type of primary studies based on the full text (*P* < 0.01), review registration based on full text (*P* < 0.01), continents of origin of the reviews (*P* < 0.01), abstract type (*P* < 0.01), review access based on website (*P* < 0.01), review access based on internet (*P* < 0.01), journal type (*P* < 0.01), journal access (*P* < 0.01), journal reporting a COI policy in the instructions to authors (*P* = 0.02), number of citations (*P* < 0.01) and number of authors (*P* < 0.01). The VIF values of all the significant variables were < 5, and therefore, those variables were all included in the subsequent multivariate analysis. The direction of the association of the significant variables in the univariate analyses are reported in Supplementary files.

**Table 4:** Univariate and multivariate binary logistic regression analysis for the reporting COI at PubMed

	Univariate (No COI reporting as the reference category)			Multivariate (all variables included)			Multivariate (backward selection)		
Variables	B	OR (95%CI)	P	B	OR (95%CI)	P	B	OR (95%CI)	P
**Type of primary studies based on the full-text**			< 0.01*			< 0.01*			< 0.01*
In-vitro or animals	Ref			Ref			Ref		
Humans	-1.25	0.29 (0.18–0.46)	< 0.01*	-1.13	0.32 (0.16–0.67)	< 0.01*	-1.15	0.32 (0.16–0.64)	< 0.01*
Both	-0.35	0.70 (0.31–1.62)	0.41	-0.57	0.57 (0.16–2.00)	0.38	-0.58	0.56 (0.17–1.91)	0.36
**Review type**									
Intervention	Ref								
Non-intervention	0.33	1.39 (0.97–1.97)	0.07						
**Review registration based on full-text**									
Yes	Ref			Ref			Ref		
No	-0.52	0.60 (0.45–0.80)	< 0.01*	-0.85	0.43 (0.27–0.67)	< 0.01*	-0.81	0.44 (0.29–0.67)	< 0.01*
**Continents of origin of the reviews**			< 0.01*			0.61			
North America	Ref			Ref					
South America	0.19	1.21 (0.67–2.18)	0.53	-0.66	0.52 (0.23–1.18)	0.12			
Europe	0.75	2.12 (1.24–3.65)	< 0.01*	-0.21	0.81 (0.39–1.67)	0.57			
Asia	0.91	2.49 (1.44–4.31)	< 0.01*	-0.18	0.84 (0.39–1.78)	0.64			
Africa	0.81	2.25 (0.68–7.45)	0.19	0.02	1.02 (0.19–5.55)	0.98			
Oceania	-0.25	0.78 (0.27–2.28)	0.65	-0.58	0.56 (0.13–2.40)	0.43			
**Countries of origin of the reviews**									
Developing	Ref								
Developed	-0.06	0.94 (0.71–1.25)	0.67						
**Abstract type**									
Structured	Ref			Ref					
Non-structured	0.47	1.60 (1.18–2.16)	< 0.01*	0.14	1.15 (0.72–1.83)	0.56			
**Review access based on homepage**									
Yes	Ref			Ref			Ref		
No	-2.30	0.10 (0.06–0.16)	< 0.01*	-1.05	0.35 (0.18–0.70)	< 0.01*	-1.11	0.33 (0.18–0.62)	< 0.01*
**Review access based on internet**									
Yes	Ref			Ref					
No	-2.65	0.07 (0.02–0.29)	< 0.01*	-0.57	0.57 (0.12–2.70)	0.48			
**Journal type**									
Dentistry	Ref			Ref			Ref		
Other	1.89	6.61 (4.58–9.55)	< 0.01*	0.86	2.36 (1.38–4.04)	< 0.01*	0.90	2.46 (1.48–4.08)	< 0.01*
**Journal access**									
Subscription or hybrid	Ref			Ref			Ref		
Open access	3.07	21.50 (15.04–30.73)	< 0.01*	2.52	12.44 (7.66–20.20)	< 0.01*	2.63	13.87 (8.75–21.97)	< 0.01*
**Journal reporting a COI policy in the instructions to authors**									
Yes	Ref			Ref					
No	1.00	2.73 (1.21–6.17)	0.02*	-0.24	0.79 (0.26–2.43)	0.68			
**Journal reporting the type of COI in the instructions to authors**			0.07						
Financial	Ref								
Non-financial	0.92	2.52 (0.15–41.87)	0.52						
Both	0.13	1.13 (0.70–1.85)	0.62						
Only say that authors need to report COI	-0.44	0.65 (0.28–1.49)	0.31						
No information	1.09	2.98 (1.18–7.53)	0.02*						
Journal impact factor**	-0.07	0.93 (0.85–1.03)	0.17						
Number of citations***	-0.11	0.989 (0.985–0.993)	< 0.01*	-0.003	0.997 (0.993–1.000)	0.09			
**Number of authors**	0.07	1.07 (1.02–1.13)	< 0.01*	0.08	1.08 (1.01–1.16)	0.03*	0.08	1.09 (1.02–1.16)	0.02*
**Nagelkerke R**^**2**^	Not applicable			0.547			0.539		

Based on the multivariate analysis with backward selection, the type of primary studies based on the full text (for humans, OR: 0.32; 95%CI: 0.16, 0.64; *P* < 0.01), review registration based on the full text (OR: 0.44; 95%CI: 0.29, 0.67; *P* < 0.01), review access based on website (OR: 0.33; 95%CI: 0.18, 0.62; *P* < 0.01), journal type (OR: 2.46; 95%CI: 1.48, 4.08; *P* < 0.01), journal access (OR: 13.87; 95%CI: 8.75, 21.97; *P* < 0.01) and number of authors (OR: 1.09; 95%CI: 1.02, 1.16; *P* = 0.02) remained statistically significant. The reviews with the primary studies in humans, no registration based on full-text, and no full access based on homepage have significantly less odds to report the COI at PubMed level than the reviews with the primary studies in-vitro and in animals, presence of registration based on full-text, and presence of full access based on homepage. In addition, the reviews published in non-dental journals, open access journals, and with bigger number of authors have significantly higher odds to report the COI at PubMed level than the reviews in dental journals, subscribed or hybrid journals, and with smaller number of authors.

Table 5 shows the associations between different characteristics of the reviews and the reporting of COI on the journals’ websites. Based on the univariate analysis, the COI reporting in the journals’ websites was significantly associated with abstract type (*P* < 0.01), review access based on website (*P* < 0.01) and journal access (*P* < 0.01). The VIF values of all the significant variables were < 5, and therefore, those variables were all included in the subsequent multivariate analysis. The direction of the association of the significant variables in the univariate analyses are reported in Supplementary files.

**Table 5:** Univariate and multivariate binary logistic regression analysis for the reporting COI at website

	Univariate (No COI reporting as the reference category)			Multivariate (all variables included)			Multivariate (backward selection)		
Variables	B	OR (95%CI)	P	B	OR (95%CI)	P	B	OR (95%CI)	P
**Type of primary studies based on the full-text**			0.61						
In-vitro or animals	Ref								
Humans	-0.04	0.96 (0.48–1.92)	0.91						
Both	-0.77	0.46 (0.10–2.24)	0.34						
**Review type**									
Intervention	Ref								
Non-intervention	0.24	1.27 (0.78–2.06)	0.34						
**Review registration based on full-text**									
Yes	Ref								
No	-0.26	0.77 (0.52–1.16)	0.21						
**Continents of origin of the reviews**			0.48						
North America	Ref								
South America	-0.23	0.80 (0.38–1.67)	0.55						
Europe	-0.27	0.76 (0.38–1.52)	0.44						
Asia	0.21	1.24 (0.63–2.42)	0.53						
Africa	-	-	-						
Oceania	-0.03	0.97 (0.29–3.22)	0.96						
**Countries of origin of the reviews**									
Developing	Ref								
Developed	-0.15	0.86 (0.58–1.29)	0.47						
**Abstract type**									
Structured	Ref			Ref			Ref		
Non-structured	-0.83	0.43 (0.25–0.74)	< 0.01*	-0.75	0.48 (0.28–0.82)	< 0.01*	-0.73	0.48 (0.28–0.83)	< 0.01*
**Review access based on homepage**									
Yes	Ref			Ref					
No	0.63	1.87 (1.25–2.79)	< 0.01*	0.19	1.21 (0.78–1.86)	0.40			
**Review access based on internet**									
Yes	Ref								
No	0.12	1.12 (0.52–2.43)	0.77						
**Journal type**									
Dentistry	Ref								
Other	-0.34	0.71 (0.40–1.26)	0.24						
**Journal access**									
Subscription or hybrid	Ref			Ref			Ref		
Open access	-1.40	0.25 (0.14–0.45)	< 0.01*	-1.25	0.29 (0.15–0.54)	< 0.01*	-1.35	0.26 (0.14–0.47)	< 0.01*
**Journal reporting a COI policy in the instructions to authors**									
Yes	Ref								
No	-0.30	0.74 (0.17–3.23)	0.69						
**Journal reporting the type of COI in the instructions to authors**			0.63						
Financial	Ref								
Non-financial	-	-	-						
Both	-0.25	0.78 (0.42–1.47)	0.44						
Only say that authors need to report COI	-1.03	0.36 (0.10–1.32)	0.12						
No information	-0.55	0.58 (0.12–2.77)	0.49						
Journal impact factor**	0.03	1.03 (0.91–1.17)	0.63						
Number of citations***	-0.002	0.998 (0.995–1.001)	0.21						
**Number of authors**	-0.02	0.98 (0.91–1.06)	0.59						
**Nagelkerke R**^**2**^	Not applicable			0.076			0.075		

Based on the multivariate analysis with backward selection, abstract type (OR: 0.48; 95%CI: 0.28, 0.83; *P* < 0.01) and journal access (OR: 0.26; 95%CI: 0.14, 0.47; *P* < 0.01) remained statistically significant. Reviews with non-structured abstracts and published in open access journals have significantly less odds to report COI at home page level than the reviews with structured abstracts and published in subscribed or hybrid journals.

Table 6 shows the association between the different characteristics of the reviews and the reporting of COI in the full texts. Based on the univariate analysis, the COI reporting in the full texts was significantly associated with review registration based on full text (*P* < 0.01), continents of origin of the reviews (*P* = 0.01), abstract type (*P* = 0.01), review access based on website (*P* < 0.01), journal type (*P* = 0.02), journal access (*P* < 0.01), number of citations (*P* < 0.01), and number of authors (*P* < 0.01). The VIF values of all the significant variables were < 5, and therefore, those variables were all included in the subsequent multivariate analysis. The direction of the association of the significant variables in the univariate analyses are reported in Supplementary files.

**Table 6:** Univariate and multivariate binary logistic regression analysis for the reporting COI at full-text

	Univariate (No COI reporting as the reference category)			Multivariate (all variables included)			Multivariate (backward selection)		
Variables	B	OR (95%CI)	P	B	OR (95%CI)	P	B	OR (95%CI)	P
**Type of primary studies based on the full-text**			0.31						
In-vitro or animals	Ref								
Humans	-0.06	0.94 (0.47–1.88)	0.86						
Both	1.50	4.48 (0.55–36.57)	0.16						
**Review type**									
Intervention	Ref								
Non-intervention	0.14	1.15 (0.69–1.91)	0.60						
**Review registration based on full-text**									
Yes	Ref			Ref			Ref		
No	-0.55	0.58 (0.40–0.85)	< 0.01*	-0.49	0.61 (0.39–0.95)	0.03*	-0.58	0.56 (0.36–0.86)	< 0.01*
**Continents of origin of the reviews**			0.01*			0.02*			0.01*
North America	Ref			Ref			Ref		
South America	-0.41	0.67 (0.33–1.35)	0.26	-0.95	0.39 (0.18–0.85)	0.02*	-0.92	0.40 (0.18–0.86)	0.02*
Europe	-0.19	0.83 (0.42–1.64)	0.59	-0.50	0.61 (0.30–1.25)	0.18	-0.43	0.65 (0.32–1.32)	0.24
Asia	0.41	1.51 (0.72–3.17)	0.28	0.13	1.14 (0.51–2.55)	0.76	0.24	1.27 (0.59–2.76)	0.55
Africa	-0.70	0.49 (0.12–2.03)	0.33	-0.78	0.46 (0.10–2.06)	0.31	-0.67	0.51 (0.12–2.25)	0.37
Oceania	-1.11	0.33 (0.12–0.88)	0.03*	-0.86	0.42 (0.15–1.24)	0.12	-0.80	0.45 (0.16–1.30)	0.14
**Countries of origin of the reviews**									
Developing	Ref								
Developed	-0.22	0.80 (0.55–1.17)	0.25						
**Abstract type**									
Structured	Ref			Ref			Ref		
Non-structured	0.64	1.90 (1.17–3.08)	0.01*	0.72	2.06 (1.22–3.48)	< 0.01*	0.69	2.00 (1.19–3.35)	< 0.01*
**Review access based on homepage**									
Yes	Ref			Ref					
No	-0.65	0.52 (0.36–0.77)	< 0.01*	-0.26	0.77 (0.48–1.23)	0.27			
**Review access based on internet**									
Yes	Ref								
No	0.34	1.41 (0.59–3.35)	0.44						
**Journal type**									
Dentistry	Ref			Ref					
Other	0.76	2.13 (1.15–3.97)	0.02*	-0.13	0.88 (0.42–1.82)	0.73			
**Journal access**									
Subscription or hybrid	Ref			Ref			Ref		
Open access	1.31	3.71 (2.15–6.40)	< 0.01*	0.96	2.62 (1.36–5.07)	< 0.01*	1.13	3.10 (1.76–5.45)	< 0.01*
**Journal reporting a COI policy in the instructions to authors**									
Yes	Ref								
No	-0.80	0.45 (0.17–1.15)	0.10						
**Journal reporting the type of COI in the instructions to authors**			0.29						
Financial	Ref								
Non-financial	-	-	-						
Both	0.44	1.56 (0.87–2.79)	0.14						
Only say that authors need to report COI	0.47	1.59 (0.58–4.38)	0.37						
No information	-0.41	0.67 (0.23–1.95)	0.46						
Journal impact factor**	-0.08	0.93 (0.83–1.04)	0.21						
Number of citations***	-0.003	0.997 (0.995–0.999)	< 0.01*	-0.002	0.998 (0.996–1.000)	0.08			
**Number of authors**	0.21	1.23 (1.11–1.37)	< 0.01*	0.22	1.25 (1.11–1.42)	< 0.01*	0.24	1.27 (1.12–1.43)	< 0.01*
**Nagelkerke R**^**2**^	Not applicable			0.146			0.139		

Based on the multivariate analysis with backward selection, review registration was based on the full text (OR: 0.56; 95%CI: 0.36, 0.86, *P* < 0.01), continents of origin of the reviews (for South America, OR: 0.40; 95%CI: 0.18, 0.86; *P* = 0.02), abstract type (OR: 2.00; 95%CI: 1.19, 3.35; *P* < 0.01), journal access (OR: 3.10; 95%CI: 1.76, 5.45; *P* < 0.01) and number of authors (OR: 1.27; 95%CI: 1.12, 1.43; *P* < 0.01) remained statistically significant. Reviews with no registration based on full-text and performed in South America have significantly less odds to report the COI at full-text level than the reviews with presence of registration based on full-text and performed in North America. In addition, the reviews with non-structured abstracts, published in open access journals, and with bigger number of authors have significantly higher odds to report the COI at full-text level than the reviews with structured abstracts, published in subscribed or hybrid journals, and with smaller number of authors.

## Discussion

### Main findings

The present study found consistent disagreement regarding the reporting of COI in abstracts of dental systematic reviews between different sources (i.e. PubMed, website, and full-text). Furthermore, the information on COI was reported in more detail in the full text of the articles than in the abstracts published on PubMed and on the journals’ websites. More than one-third of the selected articles did not report any statement on sponsorship in their full text. Regression analyses demonstrated that the reporting of COI at PubMed is significantly associated with type of primary studies, review registration, review access, journal type, journal access, and number of authors. The reporting of COI at website is significantly associated with abstract type and journal access. The reporting of COI at full-text is significantly associated with review registration, continents of origin of the reviews, abstract type, journal access, and number of authors. Journal access was significantly associated with COI statement reporting in all three sources. Abstract type, review registration based on the full text and number of authors were significantly associated with COI statement reporting in two sources.

### Interpretation of the findings

It appears that there was underreporting of statements on COI and sponsorship in both sources reporting abstracts: PubMed and the journal websites (Table 3). Furthermore, for systematic reviews that reported COI statements, the overwhelming majority (more than 96%) did not report any potential financial or non-financial COI (Table 3). The present findings are in agreement with a previous study by Faggion et al. [17] that assessed the reporting of COI and sponsorship in 1,000 articles published in dental journals. In a sample of 95 systematic reviews included in the sample of 1,000 articles, 4% reported a COI. However, the present findings are in disagreement with a study by Bou-Karroum et al. that assessed financial and non-financial COI in systematic reviews in health policy and systems research [18]. In that study, 15% of the systematic reviews had at least one author reporting at least one type of COI. However, our study reported more COI statements than it did (87% versus 80%, respectively) (Table 3). Another study by Kee et al. [19], published in the field of psoriasis, concluded that 82% of the systematic reviews assessed had at least one author with a COI. One possibility is that authors of systematic reviews published in dentistry may not report financial and non-financial COIs in detail.

There were also statistically significant differences in sponsorship reporting between the full texts and PubMed/journal websites, with the latter being underreported (Table 3). Research by Lundh et al. suggests that primary research studies on drugs and devices sponsored by their manufacturing companies have more positive results and conclusions than those studies supported by other sources [20]. Similarly, a study by Veroniki et al. suggested that industry-sponsored secondary research in the form of network meta-analyses seems to report more favourable conclusions than non–industry-sponsored network meta-analyses [14]. Nevertheless, even if readers were able to access the full text of the articles in this sample, they would not obtain any information about sponsorship in more than one-third of the articles. Therefore, improvements in reporting information on sponsorship are needed in the three different sources to allow an adequate interpretation of the findings by interested readers.

In the regression analysis, journal access was significantly associated with the COI reporting statement for all three sources. Reviews published in open-access journals were more likely to report COI statements in full texts and PubMed than reviews in subscription or hybrid journals (Tables 4 and 6). However, we also found that the reviews in open-access journals were less likely to report COI on the journals’ websites than reviews in subscription or hybrid journals (Table 5). We do not have any plausible explanations for these findings. In addition, we found that unregistered reviews were less likely to report COI in both the full text and PubMed than registered reviews (Tables 4 and 6). An article published by Stewart et al. suggests that prospective registration of the protocols can ensure transparency, robustness and accountability in the research process [21]. Systematic reviews are often committed to adhering to certain standards and guidelines set by the registration platform, and a statement of the reporting of COI is always required in many registration platforms (e.g. PROSPERO). Guidelines on the reporting of COI in registered protocols may also help/remind authors to include COI information in the abstracts and full texts of the review when they are written. This may be why registered reviews were more likely to report a COI statement. In addition, reviews with a larger number of authors were more likely to report a COI statement in both the full text and the PubMed abstract (Tables 4 and 6). A study by Wiehn et al. suggests that the number of authors was positively associated with the reporting quality of the publications [22]. This may be because a larger number of authors can lead to a more rigorous informal peer-review process prior to a manuscript’s submission, which may help enhance the overall quality of the reporting of a publication, including the reporting of a COI statement. We also found that the reviews with non-structured abstracts were less likely to report a COI statement on journals’ websites (Table 5) but more likely to report a COI statement in full texts (Table 6), compared with reviews with structured abstracts. However, the opposite conclusions are difficult to explain.

### Limitations and strengths

Although the study included a large sample of systematic reviews published in dentistry, we cannot rule out some bias in the selection of systematic reviews [23]. We included only systematic reviews with meta-analyses; therefore, evidence from systematic reviews without meta-analyses was not included. Furthermore, we included only articles indexed in PubMed and some sort of publication bias should be considered. A strength of our study is the innovative approach to investigating COI reporting in different sources.

## Conclusion

There was a lack of reporting of information about COI and sponsorship statements in three different sources (i.e. PubMed, full-texts and the journal’s website). Furthermore, there were statistically significant differences in COI and sponsorship reporting between the various sources. Because readers may not have access to all three sources, it is suggested that all sources report equivalent information on potential COIs and sponsorships in detail.

## Supplementary Information

Supplementary Material 1.

## Data Availability

The datasets used and/or analysed during the current study are available from the corresponding author on reasonable request.

## Abbreviations

COI: Conflicts of interest
RCTs: Randomised clinical trials
SDs: Standard deviations
VIF: Variance inflation factor
