# Supplementary material for: Differences in the reporting of conflicts of interest and sponsorships in systematic reviews with meta-analyses in dentistry: an examination of factors associated with their reporting
Source: Res Integr Peer Rev. 2024 Sep 30;9:10. doi: 10.1186/s41073-024-00150-y (PMC11443767; doi:10.1186/s41073-024-00150-y)
Supplement: Supplementary file 1 — Supplementary Material 1. [file 41073_2024_150_MOESM1_ESM.docx]

**Supplementary files**

*Interpretation of the univariate regression analyses results*

Regarding the association between different characteristics of the reviews (independent variables) and the reporting of COI at PubMed level (dependent variable) (Table 4), the reviews with primary studies in humans, no registration based on full-text, no full access based on homepage and internet, and larger number of citations have significantly less odds to report COI at PubMed level than the reviews with the primary studies in-vitro or animals, presence of registration based on full-text, presence of full access based on homepage and internet, and less number of citations. In addition, the reviews performed in Europe and Asia, with unstructured abstracts, published in non-dental journals, published in open access journals, published in journals reporting no COI policy in the instructions to authors, published in journals with no information on type of COI in the instructions to authors, and with larger number of authors have significantly higher odds to report COI at PubMed than the reviews performed in North America, with structured abstracts, published in dental journals, published in subscription or hybrid journals, published in journals reporting COI policy in the instructions to authors, published in journals reporting financial COI in the instructions to authors, and with less number of authors.

Regarding the association between different characteristics of the reviews (independent variables) and the reporting of COI at homepage level (dependent variable) (Table 5), the reviews with non-structured abstracts and published in open access journals have significantly less odds to report COI at homepage level than the reviews with structured abstracts and published in subscription or hybrid journals. In addition, the reviews with no full access based on homepage have significantly higher odds to report COI at homepage level than reviews with presence of full access based on homepage.

Regarding the association between different characteristics of the reviews (independent variables) and the reporting of COI at full-text level (dependent variable) (Table 6), the reviews with no registration based on full-text, performed in Oceania, with no full access based on homepage and larger number of citations have significantly less odds to report COI at full-text level than the reviews with presence of registration based on full-text, performed in North America, with presence of full access based on homepage and less number of citations. In addition, the reviews with unstructured abstracts, published in non-dental journals, published in open access journals, and with larger number of authors have significantly higher odds to report COI at full-text level than the reviews with structured abstracts, published in dental journals, published in subscription or hybrid journals, and with less number of authors.

**1. List of included articles used in the analysis**

1. de Oliveira Limírio JPJ, Lemos CAA, de Luna Gomes JM, Minatel L, Alves Rezende MCR, Pellizzer EP. A clinical comparison of 1-piece versus 2-piece implants: A systematic review and meta-analysis. J Prosthet Dent. 2020;124:439–45.

2. Handschel J, Simonowska M, Naujoks C, Depprich RA, Ommerborn MA, Meyer U, et al. A histomorphometric meta-analysis of sinus elevation with various grafting materials. Head Face Med. 2009;5:12.

3. Kumar V, Arya G, Singh P, Chauhan P. A meta analysis for evaluation of marginal bone level changes at dental implants. Natl J Maxillofac Surg. 2021;12:13–6.

4. Bain CA, Weng D, Meltzer A, Kohles SS, Stach RM. A meta-analysis evaluating the risk for implant failure in patients who smoke. Compend Contin Educ Dent. 2002;23:695–9, 702, 704 passim; quiz 708.

5. Ramamoorthi M, Narvekar A, Esfandiari S. A meta-analysis of retention systems for implant-supported prostheses in partially edentulous jaws. J Prosthet Dent. 2017;118:587–95.

6. Jadu FM, Jan AM. A meta-analysis of the efficacy and safety of managing parotid and submandibular sialoliths using sialendoscopy assisted surgery. Saudi Med J. 2014;35:1188–94.

7. Luyten J, Vierendeel M, De Roo NMC, Temmerman L, De Pauw GAM. A non-cephalometric three-dimensional appraisal of soft tissue changes by functional appliances in orthodontics: a systematic review and meta-analysis. Eur J Orthod. 2022;44:458–67.

8. Singh Gill A, Morrissey H, Rahman A. A Systematic Review and Meta-Analysis Evaluating Antibiotic Prophylaxis in Dental Implants and Extraction Procedures. Medicina (Kaunas). 2018;54:95.

9. Pjetursson BE, Sailer I, Latyshev A, Rabel K, Kohal R-J, Karasan D. A systematic review and meta-analysis evaluating the survival, the failure, and the complication rates of veneered and monolithic all-ceramic implant-supported single crowns. Clin Oral Implants Res. 2021;32 Suppl 21:254–88.

10. Pol CWP, Raghoebar GM, Kerdijk W, Boven GC, Cune MS, Meijer HJA. A systematic review and meta-analysis of 3-unit fixed dental prostheses: Are the results of 2 abutment implants comparable to the results of 2 abutment teeth? J Oral Rehabil. 2018;45:147–60.

11. Duale JMJ, Patel YA, Wu J, Hyde TP. A Systematic Review and Meta-Analysis of Baseline Ohip-Edent Scores. Eur J Prosthodont Restor Dent. 2018;26:17–23.

12. Starch-Jensen T, Aludden H, Hallman M, Dahlin C, Christensen A-E, Mordenfeld A. A systematic review and meta-analysis of long-term studies (five or more years) assessing maxillary sinus floor augmentation. Int J Oral Maxillofac Surg. 2018;47:103–16.

13. Ghanem A, Abduljabbar T, Akram Z, Vohra F, Kellesarian SV, Javed F. A systematic review and meta-analysis of pre-clinical studies assessing the effect of nicotine on osseointegration. Int J Oral Maxillofac Surg. 2017;46:496–502.

14. Zakir M, Thomas D, Adams R, Farnell D, Claydon N. A Systematic Review and Meta-Analysis of the Clinical Outcomes for Adjunctive Physical, Chemical, and Biological Treatment of Dental Implants With Peri-Implantitis. J Oral Implantol. 2023;49:168–78.

15. Shu X, He S, Lo ECM, Leung KCM. A Systematic Review and Meta-analysis of the Effect of Oral Exercises on Masticatory Function. J Dent Res. 2022;101:515–25.

16. Bagde H, Dhopte A. A Systematic Review and Meta-Analysis of the Effective Contribution of Indian Periodontists to Global Periodontal Research Advancement. Cureus. 2022;14:e33056.

17. Mankelow J, Ryan C, Taylor P, Atkinson G, Martin D. A Systematic Review and Meta-Analysis of the Effects of Biopsychosocial Pain Education upon Health Care Professional Pain Attitudes, Knowledge, Behavior and Patient Outcomes. J Pain. 2022;23:1–24.

18. dos Santos APP, Nadanovsky P, de Oliveira BH. A systematic review and meta-analysis of the effects of fluoride toothpastes on the prevention of dental caries in the primary dentition of preschool children. Community Dent Oral Epidemiol. 2013;41:1–12.

19. Gomes GH, Misawa MYO, Fernandes C, Pannuti CM, Saraiva L, Huynh-Ba G, et al. A systematic review and meta-analysis of the survival rate of implants placed in previously failed sites. Braz Oral Res. 2018;32:e27.

20. Haro Adánez M, Nishihara H, Att W. A systematic review and meta-analysis on the clinical outcome of zirconia implant-restoration complex. J Prosthodont Res. 2018;62:397–406.

21. Kotsovilis S, Fourmousis I, Karoussis IK, Bamia C. A systematic review and meta-analysis on the effect of implant length on the survival of rough-surface dental implants. J Periodontol. 2009;80:1700–18.

22. Nobre CMG, de Barros Pascoal AL, Albuquerque Souza E, Machion Shaddox L, Dos Santos Calderon P, de Aquino Martins ARL, et al. A systematic review and meta-analysis on the effects of crown lengthening on adjacent and non-adjacent sites. Clin Oral Investig. 2017;21:7–16.

23. Wendorff-Tobolla LM, Wolgin M, Wagner G, Klerings I, Dvornyk A, Kielbassa AM. A Systematic Review and Meta-Analysis on the Efficacy of Locally Delivered Adjunctive Curcumin (Curcuma longa L.) in the Treatment of Periodontitis. Biomedicines. 2023;11:481.

24. Alam MK, Kanwal B, Shqaidef A, Alswairki HJ, Alfawzan AA, Alabdullatif AI, et al. A Systematic Review and Network Meta-Analysis on the Impact of Various Aligner Materials and Attachments on Orthodontic Tooth Movement. J Funct Biomater. 2023;14:209.

25. Damerau J-M, Bierbaum S, Wiedemeier D, Korn P, Smeets R, Jenny G, et al. A systematic review on the effect of inorganic surface coatings in large animal models and meta-analysis on tricalcium phosphate and hydroxyapatite on periimplant bone formation. J Biomed Mater Res B Appl Biomater. 2022;110:157–75.

26. Ferreira JB, Christovam IO, Alencar DS, da Motta AFJ, Mattos CT, Cury-Saramago A. Accuracy and reproducibility of dental measurements on tomographic digital models: a systematic review and meta-analysis. Dentomaxillofac Radiol. 2017;46:20160455.

27. Jorba-García A, González-Barnadas A, Camps-Font O, Figueiredo R, Valmaseda-Castellón E. Accuracy assessment of dynamic computer-aided implant placement: a systematic review and meta-analysis. Clin Oral Investig. 2021;25:2479–94.

28. Yu X, Tao B, Wang F, Wu Y. Accuracy assessment of dynamic navigation during implant placement: A systematic review and meta-analysis of clinical studies in the last 10 years. J Dent. 2023;135:104567.

29. Raico Gallardo YN, da Silva-Olivio IRT, Mukai E, Morimoto S, Sesma N, Cordaro L. Accuracy comparison of guided surgery for dental implants according to the tissue of support: a systematic review and meta-analysis. Clin Oral Implants Res. 2017;28:602–12.

30. Evangelista K, de Freitas Silva BS, Yamamoto-Silva FP, Valladares-Neto J, Silva MAG, Cevidanes LHS, et al. Accuracy of artificial intelligence for tooth extraction decision-making in orthodontics: a systematic review and meta-analysis. Clin Oral Investig. 2022;26:6893–905.

31. Abesi F, Jamali AS, Zamani M. Accuracy of artificial intelligence in the detection and segmentation of oral and maxillofacial structures using cone-beam computed tomography images: a systematic review and meta-analysis. Pol J Radiol. 2023;88:e256–63.

32. Mai H-N, Dam VV, Lee D-H. Accuracy of Augmented Reality-Assisted Navigation in Dental Implant Surgery: Systematic Review and Meta-analysis. J Med Internet Res. 2023;25:e42040.

33. Serafin M, Baldini B, Cabitza F, Carrafiello G, Baselli G, Del Fabbro M, et al. Accuracy of automated 3D cephalometric landmarks by deep learning algorithms: systematic review and meta-analysis. Radiol Med. 2023;128:544–55.

34. Schnutenhaus S, Edelmann C, Knipper A, Luthardt RG. Accuracy of Dynamic Computer-Assisted Implant Placement: A Systematic Review and Meta-Analysis of Clinical and In Vitro Studies. J Clin Med. 2021;10:704.

35. Wei S-M, Zhu Y, Wei J-X, Zhang C-N, Shi J-Y, Lai H-C. Accuracy of dynamic navigation in implant surgery: A systematic review and meta-analysis. Clin Oral Implants Res. 2021;32:383–93.

36. Mai H-N, Lee D-H. Accuracy of Mobile Device-Compatible 3D Scanners for Facial Digitization: Systematic Review and Meta-Analysis. J Med Internet Res. 2020;22:e22228.

37. Manisha J, Srivastava G, Das SS, Tabarak N, Choudhury GK. Accuracy of single-unit ceramic crown fabrication after digital versus conventional impressions: A systematic review and meta-analysis. J Indian Prosthodont Soc. 2023;23:105–11.

38. Diaconescu I, Isailă O-M, Hostiuc S. Accuracy of the Chaillet’s Method for Assessing the Age in Subadults. A Meta-Analysis. Curr Health Sci J. 2021;47:196–203.

39. Hostiuc S, Edison S-E, Diaconescu I, Negoi I, Isaila O-M. Accuracy of the Demirjian’s method for assessing the age in children, from 1973 to 2020. A meta-analysis. Leg Med (Tokyo). 2021;52:101901.

40. Santiago BM, Almeida L, Cavalcanti YW, Magno MB, Maia LC. Accuracy of the third molar maturity index in assessing the legal age of 18 years: a systematic review and meta-analysis. Int J Legal Med. 2018;132:1167–84.

41. Miroshnychenko A, Ibrahim S, Azab M, Roldan Y, Martinez JPD, Tamilselvan D, et al. Acute Postoperative Pain Due to Dental Extraction in the Adult Population: A Systematic Review and Network Meta-analysis. J Dent Res. 2023;102:391–401.

42. Özcan M, Bernasconi M. Adhesion to zirconia used for dental restorations: a systematic review and meta-analysis. J Adhes Dent. 2015;17:7–26.

43. Dreweck FDS, Burey A, de Oliveira Dreweck M, Loguercio AD, Reis A. Adhesive strategies in cervical lesions: systematic review and a network meta-analysis of randomized controlled trials. Clin Oral Investig. 2021;25:2495–510.

44. Wang C-Y, Yang Y-H, Li H, Lin P-Y, Su Y-T, Kuo MY-P, et al. Adjunctive local treatments for patients with residual pockets during supportive periodontal care: A systematic review and network meta-analysis. J Clin Periodontol. 2020;47:1496–510.

45. Souza EQM, da Rocha TE, Toro LF, Guiati IZ, Freire J de OA, Ervolino E, et al. Adjuvant effects of curcumin as a photoantimicrobial or irrigant in the non-surgical treatment of periodontitis: Systematic review and meta-analysis. Photodiagnosis Photodyn Ther. 2021;34:102265.

46. Ata-Ali F, Ata-Ali J, Ferrer-Molina M, Cobo T, De Carlos F, Cobo J. Adverse effects of lingual and buccal orthodontic techniques: A systematic review and meta-analysis. Am J Orthod Dentofacial Orthop. 2016;149:820–9.

47. Atieh MA, Almatrooshi A, Shah M, Hannawi H, Tawse-Smith A, Alsabeeha NHM. Airflow for initial nonsurgical treatment of peri-implantitis: A systematic review and meta-analysis. Clin Implant Dent Relat Res. 2022;24:196–210.

48. Prince J, Goertzen C, Zanjir M, Wong M, Azarpazhooh A. Airway Complications in Intubated Versus Laryngeal Mask Airway-Managed Dentistry: A Meta-Analysis. Anesth Prog. 2021;68:193–205.

49. Monje A, Chan H-L, Galindo-Moreno P, Elnayef B, Suarez-Lopez del Amo F, Wang F, et al. Alveolar Bone Architecture: A Systematic Review and Meta-Analysis. J Periodontol. 2015;86:1231–48.

50. Guo R, Zhang L, Hu M, Huang Y, Li W. Alveolar bone changes in maxillary and mandibular anterior teeth during orthodontic treatment: A systematic review and meta-analysis. Orthod Craniofac Res. 2021;24:165–79.

51. Toledano-Osorio M, Toledano M, Manzano-Moreno FJ, Vallecillo C, Vallecillo-Rivas M, Rodriguez-Archilla A, et al. Alveolar Bone Ridge Augmentation Using Polymeric Membranes: A Systematic Review and Meta-Analysis. Polymers (Basel). 2021;13:1172.

52. Iocca O, Farcomeni A, Pardiñas Lopez S, Talib HS. Alveolar ridge preservation after tooth extraction: a Bayesian Network meta-analysis of grafting materials efficacy on prevention of bone height and width reduction. J Clin Periodontol. 2017;44:104–14.

53. Moraschini V, Fai CK, Alto RM, Dos Santos GO. Amalgam and resin composite longevity of posterior restorations: A systematic review and meta-analysis. J Dent. 2015;43:1043–50.

54. Asghar A, Priya A, Ravi KS, Iwanaga J, Tubbs RS, Naaz S, et al. An evaluation of mandibular canal variations: a systematic review and meta-analysis. Anat Sci Int. 2023;98:176–84.

55. Makowiecki A, Hadzik J, Błaszczyszyn A, Gedrange T, Dominiak M. An evaluation of superhydrophilic surfaces of dental implants - a systematic review and meta-analysis. BMC Oral Health. 2019;19:79.

56. Pourhajibagher M, Sodagar A, Bahador A. An in vitro evaluation of the effects of nanoparticles on shear bond strength and antimicrobial properties of orthodontic adhesives: A systematic review and meta-analysis study. Int Orthod. 2020;18:203–13.

57. Tong HJ, Alzahrani FS, Sim YF, Tahmassebi JF, Duggal M. Anaesthetic efficacy of articaine versus lidocaine in children’s dentistry: a systematic review and meta-analysis. Int J Paediatr Dent. 2018;28:347–60.

58. Iranmanesh P, Khazaei S, Nili M, Saatchi M, Aggarwal V, Kolahi J, et al. Anaesthetic efficacy of incorporating different additives into lidocaine for the inferior alveolar nerve block: A systematic review with meta-analysis and trial sequential analysis. Int Endod J. 2022;55:732–47.

59. Bronzato JD, Bomfim RA, Hayasida GZP, Cúri M, Estrela C, Paster BJ, et al. Analysis of microorganisms in periapical lesions: A systematic review and meta-analysis. Arch Oral Biol. 2021;124:105055.

60. Alharbi F, Almuzian M, Bearn D. Anchorage effectiveness of orthodontic miniscrews compared to headgear and transpalatal arches: a systematic review and meta-analysis. Acta Odontol Scand. 2019;77:88–98.

61. Taneja S, Singh A, Jain A. Anesthetic Effectiveness of Articaine and Lidocaine in Pediatric Patients During Dental Procedures: A Systematic Review and Meta-Analysis. Pediatr Dent. 2020;42:273–81.

62. Engelhardt S, Papacosta P, Rathe F, Özen J, Jansen JA, Junker R. Annual failure rates and marginal bone-level changes of immediate compared to conventional loading of dental implants. A systematic review of the literature and meta-analysis. Clin Oral Implants Res. 2015;26:671–87.

63. Khouly I, Braun RS, Chambrone L. Antibiotic prophylaxis may not be indicated for prevention of dental implant infections in healthy patients. A systematic review and meta-analysis. Clin Oral Investig. 2019;23:1525–53.

64. Torof E, Morrissey H, Ball PA. Antibiotic Use in Dental Implant Procedures: A Systematic Review and Meta-Analysis. Medicina (Kaunas). 2023;59:713.

65. Tampi MP, Pilcher L, Urquhart O, Kennedy E, O’Brien KK, Lockhart PB, et al. Antibiotics for the urgent management of symptomatic irreversible pulpitis, symptomatic apical periodontitis, and localized acute apical abscess: Systematic review and meta-analysis-a report of the American Dental Association. J Am Dent Assoc. 2019;150:e179–216.

66. Abe FC, Kodaira K, Motta C de CB, Barberato-Filho S, Silva MT, Guimarães CC, et al. Antimicrobial resistance of microorganisms present in periodontal diseases: A systematic review and meta-analysis. Front Microbiol. 2022;13:961986.

67. Basudan AM, Shaheen MY, de Vries RB, van den Beucken JJJP, Jansen JA, Alghamdi HS. Antiosteoporotic Drugs to Promote Bone Regeneration Related to Titanium Implants: A Systematic Review and Meta-Analysis. Tissue Eng Part B Rev. 2019;25:89–99.

68. Villanueva J, Salazar J, Alarcón A, Araya I, Yanine N, Domancic S, et al. Antiplatelet therapy in patients undergoing oral surgery: A systematic review and meta-analysis. Med Oral Patol Oral Cir Bucal. 2019;24:e103–13.

69. Ahmad MZ, Sadaf D, MacBain MM, Mohamed AN. Apical extrusion of debris with different rotary and reciprocating single-file endodontic instrumentation systems: a systematic review and meta-analysis protocol. BMJ Open. 2020;10:e038502.

70. Georgiou AC, Crielaard W, Armenis I, de Vries R, van der Waal SV. Apical Periodontitis Is Associated with Elevated Concentrations of Inflammatory Mediators in Peripheral Blood: A Systematic Review and Meta-analysis. J Endod. 2019;45:1279-1295.e3.

71. Zhang X, Xiao T, Yang L, Ning C, Guan S, Li X. Application of a vascularized bone free flap and survival rate of dental implants after transplantation: A systematic review and meta-analysis. J Stomatol Oral Maxillofac Surg. 2023;124:101401.

72. Iezzi G, Perrotti V, Felice P, Barausse C, Piattelli A, Del Fabbro M. Are <7-mm long implants in native bone as effective as longer implants in augmented bone for the rehabilitation of posterior atrophic jaws? A systematic review and meta-analysis. Clin Implant Dent Relat Res. 2020;22:552–66.

73. Lemos CAA, Nunes RG, Santiago-Júnior JF, Marcela de Luna Gomes J, Oliveira Limirio JPJ, Rosa CDDRD, et al. Are implant-supported removable partial dentures a suitable treatment for partially edentulous patients? A systematic review and meta-analysis. J Prosthet Dent. 2023;129:538–46.

74. Tang Q, Jin H, Lin S, Ma L, Tian T, Qin X. Are platelet concentrate scaffolds superior to traditional blood clot scaffolds in regeneration therapy of necrotic immature permanent teeth? A systematic review and meta-analysis. BMC Oral Health. 2022;22:589.

75. Ebrahimi M, Janani A, Majidinia S, Sadeghi R, Shirazi AS. Are self-etch adhesives reliable for primary tooth dentin? A systematic review and meta-analysis. J Conserv Dent. 2018;21:243–50.

76. Monje A, Chan H-L, Fu J-H, Suarez F, Galindo-Moreno P, Wang H-L. Are short dental implants (<10 mm) effective? a meta-analysis on prospective clinical trials. J Periodontol. 2013;84:895–904.

77. Krüsi M, Eliades T, Papageorgiou SN. Are there benefits from using bone-borne maxillary expansion instead of tooth-borne maxillary expansion? A systematic review with meta-analysis. Prog Orthod. 2019;20:9.

78. Ata-Ali F, Cobo T, De Carlos F, Cobo J, Ata-Ali J. Are there differences in treatment effects between labial and lingual fixed orthodontic appliances? A systematic review and meta-analysis. BMC Oral Health. 2017;17:133.

79. Vechiato-Filho AJ, Pesqueira AA, De Souza GM, dos Santos DM, Pellizzer EP, Goiato MC. Are Zirconia Implant Abutments Safe and Predictable in Posterior Regions? A Systematic Review and Meta-Analysis. Int J Prosthodont. 2016;29:233–44.

80. Martin E, Nimmo A, Lee A, Jennings E. Articaine in dentistry: an overview of the evidence and meta-analysis of the latest randomised controlled trials on articaine safety and efficacy compared to lidocaine for routine dental treatment. BDJ Open. 2021;7:27.

81. de Queiroz Tavares Borges Mesquita G, Vieira WA, Vidigal MTC, Travençolo BAN, Beaini TL, Spin-Neto R, et al. Artificial Intelligence for Detecting Cephalometric Landmarks: A Systematic Review and Meta-analysis. J Digit Imaging. 2023;36:1158–79.

82. Franco A, de Oliveira MN, Campos Vidigal MT, Blumenberg C, Pinheiro AA, Paranhos LR. Assessment of dental age estimation methods applied to Brazilian children: a systematic review and meta-analysis. Dentomaxillofac Radiol. 2021;50:20200128.

83. Jawdekar AM, Kamath S, Kale S, Mistry L. Assessment of oral health-related quality of life (OHRQoL) in children with molar incisor hypomineralization (MIH) - A systematic review and meta-analysis of observational studies. J Indian Soc Pedod Prev Dent. 2022;40:368–76.

84. Smolana A, Loster Z, Loster J. Assessment of stress burden among dental students: A systematic literature review and meta-analysis of data. Dent Med Probl. 2022;59:301–7.

85. Gouvêa GR, Vieira W de A, Paranhos LR, Bernardino Í de M, Bulgareli JV, Pereira AC. Assessment of the ergonomic risk from saddle and conventional seats in dentistry: A systematic review and meta-analysis. PLoS One. 2018;13:e0208900.

86. Pertek Hatipoğlu F, Mağat G, Hatipoğlu Ö, Taha N, Alfirjani S, Abidin IZ, et al. Assessment of the Prevalence of Middle Mesial Canal in Mandibular First Molar: A Multinational Cross-sectional Study with Meta-analysis. J Endod. 2023;49:549–58.

87. Hatipoğlu FP, Mağat G, Hatipoğlu Ö, Al-Khatib H, Elatrash AS, Abidin IZ, et al. Assessment of the Prevalence of Radix Entomolaris and Distolingual Canal in Mandibular First Molars in 15 Countries: A Multinational Cross-sectional Study with Meta-analysis. J Endod. 2023;49:1308–18.

88. Yang M, Peng R, Li X, Peng J, Liu L, Chen L. Association between chronic obstructive pulmonary disease and periodontal disease: a systematic review and meta-analysis. BMJ Open. 2023;13:e067432.

89. Chaffee BW, Weston SJ. Association between chronic periodontal disease and obesity: a systematic review and meta-analysis. J Periodontol. 2010;81:1708–24.

90. Castañeda-Sarmiento S, Uchima Koecklin KH, Barahona Hernandez MB, Santos GP, Bruno Luyo JC, Sánchez Sotomayor JC, et al. Association between developmental defects of enamel and early childhood caries in children under 6 years old: A systematic review and meta-analysis. Heliyon. 2022;8:e10479.

91. Jiang X, Zhu Y, Liu Z, Tian Z, Zhu S. Association between diabetes and dental implant complications: a systematic review and meta-analysis. Acta Odontol Scand. 2021;79:9–18.

92. Cabanillas-Balsera D, Martín-González J, Montero-Miralles P, Sánchez-Domínguez B, Jiménez-Sánchez MC, Segura-Egea JJ. Association between diabetes and nonretention of root filled teeth: a systematic review and meta-analysis. Int Endod J. 2019;52:297–306.

93. Segura-Egea JJ, Martín-González J, Cabanillas-Balsera D, Fouad AF, Velasco-Ortega E, López-López J. Association between diabetes and the prevalence of radiolucent periapical lesions in root-filled teeth: systematic review and meta-analysis. Clin Oral Investig. 2016;20:1133–41.

94. Monje A, Catena A, Borgnakke WS. Association between diabetes mellitus/hyperglycaemia and peri-implant diseases: Systematic review and meta-analysis. J Clin Periodontol. 2017;44:636–48.

95. Li Y, Wang Z, Fang M, Tay FR, Chen X. Association between gastro-oesophageal reflux disease and dental erosion in children: A systematic review and meta-analysis. J Dent. 2022;125:104247.

96. Nowrin SA, Jaafar S, Ab Rahman N, Basri R, Alam MK, Shahid F. Association between genetic polymorphisms and external apical root resorption: A systematic review and meta-analysis. Korean J Orthod. 2018;48:395–404.

97. Coronel-Zubiate F-T, Marroquín-Soto C, Geraldo-Campos L-A, Aguirre-Ipenza R, Urbano-Rosales L-M, Luján-Valencia S-A, et al. Association between orthodontic treatment and the occurrence of temporomandibular disorders: A systematic review and meta-analysis. J Clin Exp Dent. 2022;14:e1032–43.

98. Qi J, Liu E, Guo Y-F, Hu J-M, Liu Y-T, Chen G, et al. Association between periodontal disease and osteoporosis in postmenopausal women: a protocol for systematic review and meta-analysis. BMJ Open. 2021;11:e049277.

99. Kisely S, Baghaie H, Lalloo R, Johnson NW. Association between poor oral health and eating disorders: systematic review and meta-analysis. Br J Psychiatry. 2015;207:299–305.

100. Polmann H, Réus JC, Massignan C, Serra-Negra JM, Dick BD, Flores-Mir C, et al. Association between sleep bruxism and stress symptoms in adults: A systematic review and meta-analysis. J Oral Rehabil. 2021;48:621–31.

101. Agrawal KK, Anwar M, Gupta C, Chand P, Singh SV. Association of interleukin-1 gene polymorphism and early crestal bone loss around submerged dental implants: A systematic review and meta-analysis. J Indian Prosthodont Soc. 2021;21:116–24.

102. Firmino RT, Martins CC, Faria LDS, Martins Paiva S, Granville-Garcia AF, Fraiz FC, et al. Association of oral health literacy with oral health behaviors, perception, knowledge, and dental treatment related outcomes: a systematic review and meta-analysis. J Public Health Dent. 2018;78:231–45.

103. de Geus JL, Wambier LM, Kossatz S, Loguercio AD, Reis A. At-home vs In-office Bleaching: A Systematic Review and Meta-analysis. Oper Dent. 2016;41:341–56.

104. Prasad S, Faverani LP, Santiago Junior JF, Sukotjo C, Yuan JC-C. Attachment systems for mandibular implant-supported overdentures: A systematic review and meta-analysis of randomized controlled trials. J Prosthet Dent. 2022;S0022-3913(22)00485-1.

105. Andonovski M-E, Antonarakis GS. Autism spectrum disorder and dentoalveolar trauma: A systematic review and meta-analysis. J Stomatol Oral Maxillofac Surg. 2022;123:e858–64.

106. Meursinge Reynders R, Ronchi L, Ladu L, Di Girolamo N, de Lange J, Roberts N, et al. Barriers and facilitators to the implementation of orthodontic mini-implants in clinical practice: a protocol for a systematic review and meta-analysis. Syst Rev. 2016;5:22.

107. Gizani S, Seremidi K, Katsouli K, Markouli A, Kloukos D. Basic behavioral management techniques in pediatric dentistry: A systematic review and meta-analysis. J Dent. 2022;126:104303.

108. Parker W, Estrich CG, Abt E, Carrasco-Labra A, Waugh JB, Conway A, et al. Benefits and harms of capnography during procedures involving moderate sedation: A rapid review and meta-analysis. J Am Dent Assoc. 2018;149:38-50.e2.

109. Ślebioda Z, Woźniak T, Dorocka-Bobkowska B, Woźniewicz M, Kowalska A. Beta-defensin 1 gene polymorphisms in the pathologies of the oral cavity-Data from meta-analysis: Association only with rs1047031 not with rs1800972, rs1799946, and rs11362. J Oral Pathol Med. 2021;50:22–31.

110. da Rosa WL de O, da Silva TM, da Silva AF, Piva E. Bioactive treatments in bone grafts for implant-based rehabilitation: Systematic review and meta-analysis. Clin Implant Dent Relat Res. 2018;20:251–60.

111. Omori Y, Lang NP, Botticelli D, Papageorgiou SN, Baba S. Biological and mechanical complications of angulated abutments connected to fixed dental prostheses: A systematic review with meta-analysis. J Oral Rehabil. 2020;47:101–11.

112. Apaza Alccayhuaman KA, Soto-Peñaloza D, Nakajima Y, Papageorgiou SN, Botticelli D, Lang NP. Biological and technical complications of tilted implants in comparison with straight implants supporting fixed dental prostheses. A systematic review and meta-analysis. Clin Oral Implants Res. 2018;29 Suppl 18:295–308.

113. Sanz-Sánchez I, Sanz-Martín I, Carrillo de Albornoz A, Figuero E, Sanz M. Biological effect of the abutment material on the stability of peri-implant marginal bone levels: A systematic review and meta-analysis. Clin Oral Implants Res. 2018;29 Suppl 18:124–44.

114. Gaikwad AM, Joshi AA, Nadgere JB. Biomechanical and histomorphometric analysis of endosteal implants placed by using the osseodensification technique in animal models: A systematic review and meta-analysis. J Prosthet Dent. 2022;127:61–70.

115. Mendoza LJN, Montoya AC, Peñaloza TYM, Guerreroc CG. Biomechanical Effect of Irrigants in Noninstrumented Dentin: Systematic Review and Meta-Analysis. Crit Rev Biomed Eng. 2021;49:53–64.

116. Chrcanovic BR, Albrektsson T, Wennerberg A. Bisphosphonates and dental implants: A meta-analysis. Quintessence Int. 2016;47:329–42.

117. Hua W, Huang Z, Huang Z. Bleeding Outcomes After Dental Extraction in Patients Under Direct-Acting Oral Anticoagulants vs. Vitamin K Antagonists: A Systematic Review and Meta-Analysis. Front Pharmacol. 2021;12:702057.

118. de Andrade NK, Motta RHL, Bergamaschi C de C, Oliveira LB, Guimarães CC, Araújo J de O, et al. Bleeding Risk in Patients Using Oral Anticoagulants Undergoing Surgical Procedures in Dentistry: A Systematic Review and Meta-Analysis. Front Pharmacol. 2019;10:866.

119. Iaculli F, Salucci A, Di Giorgio G, Luzzi V, Ierardo G, Polimeni A, et al. Bond Strength of Self-Adhesive Flowable Composites and Glass Ionomer Cements to Primary Teeth: A Systematic Review and Meta-Analysis of In Vitro Studies. Materials (Basel). 2021;14:6694.

120. Dallora AL, Anderberg P, Kvist O, Mendes E, Diaz Ruiz S, Sanmartin Berglund J. Bone age assessment with various machine learning techniques: A systematic literature review and meta-analysis. PLoS One. 2019;14:e0220242.

121. de Sousa CA, Lemos CAA, Santiago-Júnior JF, Faverani LP, Pellizzer EP. Bone augmentation using autogenous bone versus biomaterial in the posterior region of atrophic mandibles: A systematic review and meta-analysis. J Dent. 2018;76:1–8.

122. DI Girolamo M, Calcaterra R, DI Gianfilippo R, Arcuri C, Baggi L. Bone level changes around platform switching and platform matching implants: a systematic review with meta-analysis. Oral Implantol (Rome). 2016;9:1–10.

123. Alghamdi HS, Junker R, Bronkhorst EM, Jansen JA. Bone regeneration related to calcium phosphate-coated implants in osteoporotic animal models: a meta-analysis. Tissue Eng Part B Rev. 2012;18:383–95.

124. Sumangali A, Naik AC, Mohan N, Gautam N, Abrol S, Mustafa M, et al. Bone Regenerative Biomaterials in Periapical Surgery: A Systemic Review and Meta-Analysis. J Pharm Bioallied Sci. 2021;13:S933–7.

125. Shanbhag S, Pandis N, Mustafa K, Nyengaard JR, Stavropoulos A. Bone tissue engineering in oral peri-implant defects in preclinical in vivo research: A systematic review and meta-analysis. J Tissue Eng Regen Med. 2018;12:e336–49.

126. Chrcanovic BR, Albrektsson T, Wennerberg A. Bruxism and Dental Implants: A Meta-Analysis. Implant Dent. 2015;24:505–16.

127. Sanz-Martín I, Regidor E, Cosyn J, Wiedemeier DB, Thoma DS. Buccal soft tissue dehiscence defects at dental implants-associated factors and frequency of occurrence: A systematic review and meta-analysis. Clin Oral Implants Res. 2022;33 Suppl 23:109–24.

128. Srinivasan M, Kamnoedboon P, McKenna G, Angst L, Schimmel M, Özcan M, et al. CAD-CAM removable complete dentures: A systematic review and meta-analysis of trueness of fit, biocompatibility, mechanical properties, surface characteristics, color stability, time-cost analysis, clinical and patient-reported outcomes. J Dent. 2021;113:103777.

129. Lops D, Romeo E, Mensi M, Troiano G, Zhurakivska K, Del Fabbro M, et al. CAD/CAM Abutments in the Esthetic Zone: A Systematic Review and Meta-Analysis of Soft Tissue Stability. J Clin Med. 2023;12:3847.

130. Rodrigues SB, Franken P, Celeste RK, Leitune VCB, Collares FM. CAD/CAM or conventional ceramic materials restorations longevity: a systematic review and meta-analysis. J Prosthodont Res. 2019;63:389–95.

131. Santiago Junior JF, Biguetti CC, Matsumoto MA, Abu Halawa Kudo G, Parra da Silva RB, Pinto Saraiva P, et al. Can Genetic Factors Compromise the Success of Dental Implants? A Systematic Review and Meta-Analysis. Genes (Basel). 2018;9:444.

132. Miguita L, Mantesso A, Pannuti CM, Deboni MCZ. Can stem cells enhance bone formation in the human edentulous alveolar ridge? A systematic review and meta-analysis. Cell Tissue Bank. 2017;18:217–28.

133. Xiao J, Huang X, Alkhers N, Alzamil H, Alzoubi S, Wu TT, et al. Candida albicans and Early Childhood Caries: A Systematic Review and Meta-Analysis. Caries Res. 2018;52:102–12.

134. Askar H, Misch J, Chen Z, Chadha S, Wang H-L. Capnography monitoring in procedural intravenous sedation: a systematic review and meta-analysis. Clin Oral Investig. 2020;24:3761–70.

135. Schwendicke F, Göstemeyer G, Gluud C. Cavity lining after excavating caries lesions: meta-analysis and trial sequential analysis of randomized clinical trials. J Dent. 2015;43:1291–7.

136. de Toledo Telles-Araújo G, Peralta-Mamani M, Caminha RDG, de Fatima Moraes-da-Silva A, Rubira CMF, Honório HM, et al. CBCT does not reduce neurosensory disturbances after third molar removal compared to panoramic radiography: a systematic review and meta-analysis. Clin Oral Investig. 2020;24:1137–49.

137. Merdietio Boedi R, Shepherd S, Mânica S, Franco A. CBCT in dental age estimation: A systematic review and meta analysis. Dentomaxillofac Radiol. 2022;51:20210335.

138. Janson G, Aliaga-Del Castillo A, Niederberger A. Changes in apical base sagittal relationship in Class II malocclusion treatment with and without premolar extractions: A systematic review and meta-analysis. Angle Orthod. 2017;87:338–55.

139. Domingo-Clérigues M, Montiel-Company J-M, Almerich-Silla J-M, García-Sanz V, Paredes-Gallardo V, Bellot-Arcís C. Changes in the alveolar bone thickness of maxillary incisors after orthodontic treatment involving extractions - A systematic review and meta-analysis. J Clin Exp Dent. 2019;11:e76–84.

140. Elkady DM, Khater AGA, Schwendicke F. Chlorhexidine to improve the survival of ART restorations: A systematic review and meta-analysis. J Dent. 2020;103:103491.

141. Cabanillas-Balsera D, Segura-Egea JJ, Jiménez-Sánchez MC, Areal-Quecuty V, Sánchez-Domínguez B, Montero-Miralles P, et al. Cigarette Smoking and Root Filled Teeth Extraction: Systematic Review and Meta-Analysis. J Clin Med. 2020;9:3179.

142. Németh A, Vitai V, Czumbel ML, Szabó B, Varga G, Kerémi B, et al. Clear guidance to select the most accurate technologies for 3D printing dental models - A network meta-analysis✰. J Dent. 2023;134:104532.

143. Mareque S, Castelo-Baz P, López-Malla J, Blanco J, Nart J, Vallés C. Clinical and esthetic outcomes of immediate implant placement compared to alveolar ridge preservation: a systematic review and meta-analysis. Clin Oral Investig. 2021;25:4735–48.

144. Firoozi P, Salman BN, Aslaminabadi N. Clinical and radiographic comparison of Biodentine and Formocresol: an updated meta-analysis with trial sequential analysis. Eur Arch Paediatr Dent. 2022;23:855–67.

145. Marghalani AA, Omar S, Chen J-W. Clinical and radiographic success of mineral trioxide aggregate compared with formocresol as a pulpotomy treatment in primary molars: a systematic review and meta-analysis. J Am Dent Assoc. 2014;145:714–21.

146. Chen M-H, Shi J-Y. Clinical and Radiological Outcomes of Implants in Osteotome Sinus Floor Elevation with and without Grafting: A Systematic Review and a Meta-Analysis. J Prosthodont. 2018;27:394–401.

147. Guan S, Xiao T, Bai J, Ning C, Zhang X, Yang L, et al. Clinical application of platelet-rich fibrin to enhance dental implant stability: A systematic review and meta-analysis. Heliyon. 2023;9:e13196.

148. Sánchez-Labrador L, Molinero-Mourelle P, Cortés-Bretón Brinkmann J, Prados-Frutos JC, Gómez-Polo M, Martínez-González JM. Clinical Behavior and Complications of Mandibular Full-Arch Fixed Dental Prostheses Supported by Three Dental Implants. A Systematic Review and Meta-Analysis. Biology (Basel). 2021;10:308.

149. Torres-Alemany A, Fernández-Estevan L, Agustín-Panadero R, Montiel-Company JM, Labaig-Rueda C, Mañes-Ferrer JF. Clinical Behavior of Short Dental Implants: Systematic Review and Meta-Analysis. J Clin Med. 2020;9:3271.

150. Cruz RS, Lemos CAA, de Luna Gomes JM, Fernandes E Oliveira HF, Pellizzer EP, Verri FR. Clinical comparison between crestal and subcrestal dental implants: A systematic review and meta-analysis. J Prosthet Dent. 2022;127:408–17.

151. Chugh VK, Patnana AK, Chugh A, Kumar P, Wadhwa P, Singh S. Clinical differences of hand and rotary instrumentations during biomechanical preparation in primary teeth-A systematic review and meta-analysis. Int J Paediatr Dent. 2021;31:131–42.

152. Lemos CAA, Verri FR, de Oliveira Neto OB, Cruz RS, Luna Gomes JM, da Silva Casado BG, et al. Clinical effect of the high insertion torque on dental implants: A systematic review and meta-analysis. J Prosthet Dent. 2021;126:490–6.

153. Wang J, Yang Y, Wang Y, Zhang L, Ji W, Hong Z, et al. Clinical effectiveness of different types of bone-anchored maxillary protraction devices for skeletal Class III malocclusion: Systematic review and network meta-analysis. Korean J Orthod. 2022;52:313–23.

154. Huang S, Chen Y, Chen T, Mallineni SK, McGrath C, Hagg U. Clinical effectiveness of the Eruption Guidance Appliances in treating malocclusion in the mixed dentition: A systematic review and meta-analysis. Int J Paediatr Dent. 2022;32:843–55.

155. Patini R, Spagnuolo G, Guglielmi F, Staderini E, Simeone M, Camodeca A, et al. Clinical Effects of Mercury in Conservative Dentistry: A Systematic Review, Meta-Analysis, and Trial Sequential Analysis of Randomized Controlled Trials. Int J Dent. 2020;2020:8857238.

156. Guo Y, Su L, Chen C, Liu Y, Li J. Clinical Efficacy and Safety of Different Dental Prosthetic Membranes in Guided Bone Regeneration during Dental Implants: A Meta-Analysis. Comput Intell Neurosci. 2022;2022:3245014.

157. Nagay BE, Dini C, Borges GA, Mesquita MF, Cavalcanti YW, Magno MB, et al. Clinical efficacy of anodized dental implants for implant-supported prostheses after different loading protocols: A systematic review and meta-analysis. Clin Oral Implants Res. 2021;32:1021–40.

158. Zhang W, Huang S, Ye Q, Wei D, Zhou X. Clinical efficacy of early and delayed loading implants: A systematic review and meta-analysis. J Prosthet Dent. 2022;S0022-3913(22)00423-1.

159. Egido-Moreno S, Valls-Roca-Umbert J, Céspedes-Sánchez JM, López-López J, Velasco-Ortega E. Clinical Efficacy of Mesenchymal Stem Cells in Bone Regeneration in Oral Implantology. Systematic Review and Meta-Analysis. Int J Environ Res Public Health. 2021;18:894.

160. Tan OL, Safii SH, Razali M. Clinical Efficacy of Single Application Local Drug Delivery and Adjunctive Agents in Nonsurgical Periodontal Therapy: A Systematic Review and Network Meta-Analysis. Pharmaceutics. 2020;12:1086.

161. Moraschini V, Guimarães HB, Cavalcante IC, Calasans-Maia MD. Clinical efficacy of xenogeneic collagen matrix in augmenting keratinized mucosa round dental implants: a systematic review and meta-analysis. Clin Oral Investig. 2020;24:2163–74.

162. Oliveira HFE, Verri F, Lemos CA, Cruz R, Batista VE de S, Pellizzer E, et al. Clinical Evidence for Treatment of Class II Periodontal Furcation Defects. Systematic Review and Meta-analysis. J Int Acad Periodontol. 2020;22:117–28.

163. Altuna P, Lucas-Taulé E, Gargallo-Albiol J, Figueras-Álvarez O, Hernández-Alfaro F, Nart J. Clinical evidence on titanium-zirconium dental implants: a systematic review and meta-analysis. Int J Oral Maxillofac Surg. 2016;45:842–50.

164. Zhou W, Liu Z, Song L, Kuo C-L, Shafer DM. Clinical Factors Affecting the Accuracy of Guided Implant Surgery-A Systematic Review and Meta-analysis. J Evid Based Dent Pract. 2018;18:28–40.

165. Pieralli S, Kohal R-J, Rabel K, von Stein-Lausnitz M, Vach K, Spies BC. Clinical outcomes of partial and full-arch all-ceramic implant-supported fixed dental prostheses. A systematic review and meta-analysis. Clin Oral Implants Res. 2018;29 Suppl 18:224–36.

166. Araujo RZ, Santiago Júnior JF, Cardoso CL, Benites Condezo AF, Moreira Júnior R, Curi MM. Clinical outcomes of pterygoid implants: Systematic review and meta-analysis. J Craniomaxillofac Surg. 2019;47:651–60.

167. Ravidà A, Galli M, Bianchi M, Parisi E, Saleh MHA, Stacchi C, et al. Clinical outcomes of short implants (≤ 6 mm) placed between two adjacent teeth/implants or in the most distal position: A systematic review and meta-analysis. Int J Oral Implantol (Berl). 2021;14:241–57.

168. Mailoa J, Lin G-H, Chan H-L, MacEachern M, Wang H-L. Clinical outcomes of using lasers for peri-implantitis surface detoxification: a systematic review and meta-analysis. J Periodontol. 2014;85:1194–202.

169. Antonoglou GN, Stavropoulos A, Samara MD, Ioannidis A, Benic GI, Papageorgiou SN, et al. Clinical Performance of Dental Implants Following Sinus Floor Augmentation: A Systematic Review and Meta-Analysis of Clinical Trials with at Least 3 Years of Follow-up. Int J Oral Maxillofac Implants. 2018;33:e45–65.

170. Karl M, Albrektsson T. Clinical Performance of Dental Implants with a Moderately Rough (TiUnite) Surface: A Meta-Analysis of Prospective Clinical Studies. Int J Oral Maxillofac Implants. 2017;32:717–34.

171. Vargas-Moreno VF, Ribeiro MC de O, Gomes RS, Faot F, Del Bel Cury AA, Marcello-Machado RM. Clinical performance of short and extrashort dental implants with wide diameter: A systematic review with meta-analysis. J Prosthet Dent. 2023;S0022-3913(23)00010-0.

172. Duan C, Ye L, Zhang M, Yang L, Li C, Pan J, et al. Clinical performance of zirconium implants compared to titanium implants: a systematic review and meta-analysis of randomized controlled trials. PeerJ. 2023;11:e15010.

173. Wong AW-Y, Zhang S, Li SK-Y, Zhang C, Chu C-H. Clinical studies on core-carrier obturation: a systematic review and meta-analysis. BMC Oral Health. 2017;17:167.

174. Vatankhah M, Najary S, Dianat O. Clinical, Radiographic, and Histologic Outcomes of Regenerative Endodontic Treatment in Human Immature Teeth Using Different Biological Scaffolds: A Systematic Review and Meta-analysis. Curr Stem Cell Res Ther. 2022;

175. Murugaiyan S, Srinivasan B, Kailasam V. Collum angle of the anterior teeth: A systematic review and meta-analysis. J Esthet Restor Dent. 2022;34:604–19.

176. Fernandes G, Costa B, Trindade HF, Castilho RM, Fernandes J. Comparative analysis between extra-short implants (≤6 mm) and 6 mm-longer implants: a meta-analysis of randomized controlled trial. Aust Dent J. 2022;67:194–211.

177. Oza RR, Sharma V, Suryawanshi T, Lulla S, Bajaj P, Dhadse P. Comparative Analysis of Sedative Efficacy of Dexmedetomidine and Midazolam in Pediatric Dental Practice: A Systematic Review and Meta-Analysis. Cureus. 2022;14:e28452.

178. Sağirkaya E, Kucukekenci AS, Karasoy D, Akça K, Eckert SE, Çehreli MC. Comparative assessments, meta-analysis, and recommended guidelines for reporting studies on histomorphometric bone-implant contact in humans. Int J Oral Maxillofac Implants. 2013;28:1243–53.

179. Papageorgiou SN, Papageorgiou PN, Deschner J, Götz W. Comparative effectiveness of natural and synthetic bone grafts in oral and maxillofacial surgery prior to insertion of dental implants: Systematic review and network meta-analysis of parallel and cluster randomized controlled trials. J Dent. 2016;48:1–8.

180. Hegde VR, Jain A, Patekar SB. Comparative evaluation of calcium hydroxide and other intracanal medicaments on postoperative pain in patients undergoing endodontic treatment: A systematic review and meta-analysis. J Conserv Dent. 2023;26:134–42.

181. Juan-Montesinos A, Agustín-Panadero R, Solá-Ruiz MF, Marco-Pitarch R, Montiel-Company JM, Fons-Badal C. Comparative Study by Systematic Review and Meta-Analysis of the Peri-Implant Effect of Two Types of Platforms: Platform-Switching versus Conventional Platforms. J Clin Med. 2022;11:1743.

182. Akbarzadeh Baghban A, Dehghani A, Ghanavati F, Zayeri F, Ghanavati F. Comparing alveolar bone regeneration using Bio-Oss and autogenous bone grafts in humans: a systematic review and meta-analysis. Iran Endod J. 2009;4:125–30.

183. Thomas RM, Kelly A, Tagiyeva N, Kanagasingam S. Comparing endocrown restorations on permanent molars and premolars: a systematic review and meta-analysis. Br Dent J. 2020;

184. Shaikh MS, Zafar MS, Alnazzawi A. Comparing Nanohydroxyapatite Graft and Other Bone Grafts in the Repair of Periodontal Infrabony Lesions: A Systematic Review and Meta-Analysis. Int J Mol Sci. 2021;22:12021.

185. Bitaraf T, Keshtkar A, Rokn AR, Monzavi A, Geramy A, Hashemi K. Comparing short dental implant and standard dental implant in terms of marginal bone level changes: A systematic review and meta-analysis of randomized controlled trials. Clin Implant Dent Relat Res. 2019;21:796–812.

186. Eini E, Yousefimanesh H, Ashtiani AH, Saki-Malehi A, Olapour A, Rahim F. Comparing success of immediate versus delay loading of implants in fresh sockets: a systematic review and meta-analysis. Oral Maxillofac Surg. 2022;26:185–94.

187. Yogui FC, Verri FR, de Luna Gomes JM, Lemos C a. A, Cruz RS, Pellizzer EP. Comparison between computer-guided and freehand dental implant placement surgery: A systematic review and meta-analysis. Int J Oral Maxillofac Surg. 2021;50:242–50.

188. Afzal E, Fida M, Malik DS, Irfan S, Gul M. Comparison between conventional and piezocision-assisted orthodontics in relieving anterior crowding: a systematic review and meta-analysis. Eur J Orthod. 2021;43:360–6.

189. Lemos C a. A, Verri FR, Cruz RS, Gomes JML, Dos Santos DM, Goiato MC, et al. Comparison between flapless and open-flap implant placement: a systematic review and meta-analysis. Int J Oral Maxillofac Surg. 2020;49:1220–31.

190. Dos Reis-Prado AH, Abreu LG, Tavares WLF, Peixoto IF da C, Viana ACD, de Oliveira EMC, et al. Comparison between immediate and delayed post space preparations: a systematic review and meta-analysis. Clin Oral Investig. 2021;25:417–40.

191. Liu Y, Yang Z-J, Zhou J, Xiong P, Wang Q, Yang Y, et al. Comparison of Anchorage Efficiency of Orthodontic Mini-implant and Conventional Anchorage Reinforcement in Patients Requiring Maximum Orthodontic Anchorage: A Systematic Review and Meta-analysis. J Evid Based Dent Pract. 2020;20:101401.

192. Malik DES, Fida M, Afzal E, Irfan S. Comparison of anchorage loss between conventional and self-ligating brackets during canine retraction - A systematic review and meta-analysis. Int Orthod. 2020;18:41–53.

193. Garcia-Sanchez R, Dopico J, Kalemaj Z, Buti J, Pardo Zamora G, Mardas N. Comparison of clinical outcomes of immediate versus delayed placement of dental implants: A systematic review and meta-analysis. Clin Oral Implants Res. 2022;33:231–77.

194. Luque-Martinez I, Reis A, Schroeder M, Muñoz MA, Loguercio AD, Masterson D, et al. Comparison of efficacy of tray-delivered carbamide and hydrogen peroxide for at-home bleaching: a systematic review and meta-analysis. Clin Oral Investig. 2016;20:1419–33.

195. Gao X, Qin S, Cai H, Wan Q. Comparison of general and aesthetic effects between flapless and flap techniques in dental implantation: a meta-analysis of randomized controlled trials. Int J Implant Dent. 2021;7:100.

196. Sivaramakrishnan G, Sridharan K. Comparison of implant supported mandibular overdentures and conventional dentures on quality of life: a systematic review and meta-analysis of randomized controlled studies. Aust Dent J. 2016;61:482–8.

197. Raee A, Alikhasi M, Nowzari H, Djalalinia S, Khoshkam V, Moslemi N. Comparison of peri-implant clinical outcomes of digitally customized and prefabricated abutments: A systematic review and meta-analysis. Clin Implant Dent Relat Res. 2021;23:216–27.

198. Theodoridis C, Doulkeridou C, Menexes G, Vouros I. Comparison of RANKL and OPG levels in peri-implant crevicular fluid between healthy and diseased peri-implant tissues. A systematic review and meta-analysis. Clin Oral Investig. 2022;26:823–36.

199. Kerayechian N, Bardideh E, Bayani S. Comparison of self-etch primers with conventional acid-etch technique for bonding brackets in orthodontics: a systematic review and meta-analysis. Eur J Orthod. 2022;44:385–95.

200. Rocha RS, Vianna CP, Trojan LC, Padovan LEM, Dos Santos MCGL. Comparison of sinusitis rate after sinus lift procedure and zygomatic implant surgery: a meta-analysis. Oral Maxillofac Surg. 2023;

201. Alrabiah M. Comparison of survival rate and crestal bone loss of narrow diameter dental implants versus regular dental implants: A systematic review and meta-analysis. J Investig Clin Dent. 2019;10:e12367.

202. Cruz RS, Lemos CAA, Oliveira HFF, de Souza Batista VE, Pellizzer EP, Verri FR. Comparison of the Use of Titanium-Zirconium Alloy and Titanium Alloy in Dental Implants: A Systematic Review and Meta-Analysis. J Oral Implantol. 2018;44:305–12.

203. Rezende Oliveira S, Fernandes Araújo Almeida T, Aparecida da Silva T, Alves Mesquita R, Guimarães Abreu L. Comparison of tissue artifacts in punch and scalpel biopsies of oral and maxillofacial lesions: A systematic review and meta-analysis. J Stomatol Oral Maxillofac Surg. 2020;121:704–12.

204. Troiano G, Laino L, Cicciù M, Cervino G, Fiorillo L, D’amico C, et al. Comparison of Two Routes of Administration of Dexamethasone to Reduce the Postoperative Sequelae After Third Molar Surgery: A Systematic Review and Meta-Analysis. Open Dent J. 2018;12:181–8.

205. Pickert FN, Spalthoff S, Gellrich N-C, Blaya Tárraga JA. Cone-beam computed tomographic evaluation of dimensional hard tissue changes following alveolar ridge preservation techniques of different bone substitutes: a systematic review and meta-analysis. J Periodontal Implant Sci. 2022;52:3–27.

206. Dubey P, Narasimhan M, Sehgal NK, Yanni P, Kim JW, Kapila YL, et al. Connective Tissue Graft with or without Enamel Matrix Derivative for Treating Gingival Recession Defects: A Systematic Review and Meta-Analysis. J Evid Based Dent Pract. 2021;21:101635.

207. Khajezadeh M, Mohseni F, Khaledi A, Firoozeh A. Contamination of dental unit water lines (DUWL) with Legionella pneumophila and Pseudomonas aeruginosa; A Middle East systematic review and meta-analysis. Eur J Microbiol Immunol (Bp). 2023;12:93–9.

208. Stratigaki E, Tong HJ, Seremidi K, Kloukos D, Duggal M, Gizani S. Contemporary management of deep caries in primary teeth: a systematic review and meta-analysis. Eur Arch Paediatr Dent. 2022;23:695–725.

209. Oliveira BH, Cunha-Cruz J, Rajendra A, Niederman R. Controlling caries in exposed root surfaces with silver diamine fluoride: A systematic review with meta-analysis. J Am Dent Assoc. 2018;149:671-679.e1.

210. Sendyk DI, de Oliveira NK, Pannuti CM, da Graça Naclério-Homem M, Wennerberg A, Deboni MCZ. Conventional Drilling Versus Piezosurgery for Implant Site Preparation: A Meta-Analysis. J Oral Implantol. 2018;44:400–5.

211. Alqaderi H, Lee C-T, Borzangy S, Pagonis TC. Coronal pulpotomy for cariously exposed permanent posterior teeth with closed apices: A systematic review and meta-analysis. J Dent. 2016;44:1–7.

212. Dan AEB, Thygesen TH, Pinholt EM. Corticosteroid administration in oral and orthognathic surgery: a systematic review of the literature and meta-analysis. J Oral Maxillofac Surg. 2010;68:2207–20.

213. Zimmo N, Sal MHA, Sinjab K, Wang C-W, Mandelaris G, Wang H-L. Corticotomy-Assisted Orthodontics for Canine Distalization: A Systematic Review and Meta-Analysis of Clinical Controlled Trials. J Int Acad Periodontol. 2018;20:153–62.

214. Messaoudi Y, Kiliaridis S, Antonarakis GS. Craniofacial Cephalometric Characteristics and Open Bite Deformity in Individuals with Amelogenesis Imperfecta-A Systematic Review and Meta-Analysis. J Clin Med. 2023;12:3826.

215. van Eekeren PJ, Tahmaseb A, Wismeijer D. Crestal Bone Changes Around Implants with Implant-Abutment Connections at Epicrestal Level or Above: Systematic Review and Meta-Analysis. Int J Oral Maxillofac Implants. 2016;31:119–24.

216. do Nascimento-Júnior EM, Dos Santos GMS, Tavares Mendes ML, Cenci M, Correa MB, Pereira-Cenci T, et al. Cryotherapy in reducing pain, trismus, and facial swelling after third-molar surgery: Systematic review and meta-analysis of randomized clinical trials. J Am Dent Assoc. 2019;150:269-277.e1.

217. Fleming PS, Eliades T, Katsaros C, Pandis N. Curing lights for orthodontic bonding: a systematic review and meta-analysis. Am J Orthod Dentofacial Orthop. 2013;143:S92-103.

218. Alfadda SA. Current Evidence on Dental Implants Outcomes in Smokers and Nonsmokers: A Systematic Review and Meta-Analysis. J Oral Implantol. 2018;44:390–9.

219. Pedano MS, Li X, Yoshihara K, Landuyt KV, Van Meerbeek B. Cytotoxicity and Bioactivity of Dental Pulp-Capping Agents towards Human Tooth-Pulp Cells: A Systematic Review of In-Vitro Studies and Meta-Analysis of Randomized and Controlled Clinical Trials. Materials (Basel). 2020;13:2670.

220. Schwendicke F, Chaurasia A, Arsiwala L, Lee J-H, Elhennawy K, Jost-Brinkmann P-G, et al. Deep learning for cephalometric landmark detection: systematic review and meta-analysis. Clin Oral Investig. 2021;25:4299–309.

221. Chaurasia A, Namachivayam A, Koca-Ünsal RB, Lee J-H. Deep-learning performance in identifying and classifying dental implant systems from dental imaging: a systematic review and meta-analysis. J Periodontal Implant Sci. 2023;

222. Pinto PHV, Fares LC, Silva RHA da. Dental age estimation by cementum incremental lines counting: A systematic review and meta-analysis. Forensic Sci Int. 2022;341:111492.

223. Tiwari S, Kulkarni P, Agrawal N, Mali S, Kale S, Jaiswal N. Dental Anxiety Scales Used in Pediatric Dentistry: A Systematic Review and Meta-analysis. J Contemp Dent Pract. 2021;22:1338–45.

224. Khan SQ, Khan NB, Arrejaie AS. Dental caries. A meta analysis on a Saudi population. Saudi Med J. 2013;34:744–9.

225. Rabiei M, Homaie Rad E, Khosousi Sani M, Khosousi Sani S. Dental Environment Stress and The Related Factors in the Iranian Dental Students: A Systematic Review and Meta-Analysis. Med J Islam Repub Iran. 2021;35:190.

226. Sendyk DI, Rovai ES, Pannuti CM, Deboni MCZ, Sendyk WR, Wennerberg A. Dental implant loss in older versus younger patients: a systematic review and meta-analysis of prospective studies. J Oral Rehabil. 2017;44:229–36.

227. Ramanauskaite A, Borges T, Almeida BL, Correia A. Dental Implant Outcomes in Grafted Sockets: a Systematic Review and Meta-Analysis. J Oral Maxillofac Res. 2019;10:e8.

228. Panchal H, Shamsunder MG, Petrovic I, Rosen EB, Allen RJ, Hernandez M, et al. Dental Implant Survival in Vascularized Bone Flaps: A Systematic Review and Meta-Analysis. Plast Reconstr Surg. 2020;146:637–48.

229. Gupta S, Mortellaro C, Panda S, Rovati M, Giacomello MS, Colletti L, et al. Dental implant survival rate in irradiated and non-radiated patients: a systematic review and meta-analysis. J Biol Regul Homeost Agents. 2021;35:53–65.

230. Dawoud BES, Kent S, Tabbenor O, George P, Dhanda J. Dental implants and risk of bleeding in patients on oral anticoagulants: a systematic review and meta-analysis. Int J Implant Dent. 2021;7:82.

231. Elagib MFA, Alqaysi MAH, Almushayt MOS, Nagate RR, Gokhale ST, Chaturvedi S. Dental implants in growing patients: A systematic review and meta-analysis. Technol Health Care. 2023;31:1051–64.

232. Duttenhoefer F, Fuessinger MA, Beckmann Y, Schmelzeisen R, Groetz KA, Boeker M. Dental implants in immunocompromised patients: a systematic review and meta-analysis. Int J Implant Dent. 2019;5:43.

233. Chrcanovic BR, Albrektsson T, Wennerberg A. Dental implants in irradiated versus nonirradiated patients: A meta-analysis. Head Neck. 2016;38:448–81.

234. Chrcanovic BR, Albrektsson T, Wennerberg A. Dental Implants in Patients Receiving Chemotherapy: A Meta-Analysis. Implant Dent. 2016;25:261–71.

235. Schiegnitz E, Reinicke K, Sagheb K, König J, Al-Nawas B, Grötz KA. Dental implants in patients with head and neck cancer-A systematic review and meta-analysis of the influence of radiotherapy on implant survival. Clin Oral Implants Res. 2022;33:967–99.

236. de Medeiros FCFL, Kudo G a. H, Leme BG, Saraiva PP, Verri FR, Honório HM, et al. Dental implants in patients with osteoporosis: a systematic review with meta-analysis. Int J Oral Maxillofac Surg. 2018;47:480–91.

237. Srinivasan M, Meyer S, Mombelli A, Müller F. Dental implants in the elderly population: a systematic review and meta-analysis. Clin Oral Implants Res. 2017;28:920–30.

238. Chrcanovic BR, Albrektsson T, Wennerberg A. Dental implants inserted in fresh extraction sockets versus healed sites: a systematic review and meta-analysis. J Dent. 2015;43:16–41.

239. Ibrahim A, Chrcanovic BR. Dental Implants Inserted in Fresh Extraction Sockets versus Healed Sites: A Systematic Review and Meta-Analysis. Materials (Basel). 2021;14:7903.

240. Chrcanovic BR, Albrektsson T, Wennerberg A. Dental implants inserted in male versus female patients: a systematic review and meta-analysis. J Oral Rehabil. 2015;42:709–22.

241. Martins CC, Firmino RT, Riva JJ, Ge L, Carrasco-Labra A, Brignardello-Petersen R, et al. Desensitizing Toothpastes for Dentin Hypersensitivity: A Network Meta-analysis. J Dent Res. 2020;99:514–22.

242. Brouwer F, Askar H, Paris S, Schwendicke F. Detecting Secondary Caries Lesions: A Systematic Review and Meta-analysis. J Dent Res. 2016;95:143–51.

243. Dalessandri D, Salgarello S, Dalessandri M, Lazzaroni E, Piancino M, Paganelli C, et al. Determinants for success rates of temporary anchorage devices in orthodontics: a meta-analysis (n > 50). Eur J Orthod. 2014;36:303–13.

244. Reale MC, Riche DM, Witt BA, Baker WL, Peeters MJ. Development of critical thinking in health professions education: A meta-analysis of longitudinal studies. Curr Pharm Teach Learn. 2018;10:826–33.

245. Costa FS, Silveira ER, Pinto GS, Nascimento GG, Thomson WM, Demarco FF. Developmental defects of enamel and dental caries in the primary dentition: A systematic review and meta-analysis. J Dent. 2017;60:1–7.

246. Al Ansari Y, Shahwan H, Chrcanovic BR. Diabetes Mellitus and Dental Implants: A Systematic Review and Meta-Analysis. Materials (Basel). 2022;15:3227.

247. Chagas MM, Kobayashi-Velasco S, Gimenez T, Cavalcanti MGP. Diagnostic accuracy of imaging examinations for peri-implant bone defects around titanium and zirconium dioxide implants: A systematic review and meta-analysis. Imaging Sci Dent. 2021;51:363–72.

248. Patro S, Meto A, Mohanty A, Chopra V, Miglani S, Das A, et al. Diagnostic Accuracy of Pulp Vitality Tests and Pulp Sensibility Tests for Assessing Pulpal Health in Permanent Teeth: A Systematic Review and Meta-Analysis. Int J Environ Res Public Health. 2022;19:9599.

249. Thapar PR, Nadgere JB, Iyer J, Salvi NA. Diagnostic accuracy of ultrasonography compared with magnetic resonance imaging in diagnosing disc displacement of the temporomandibular joint: A systematic review and meta-analysis. J Prosthet Dent. 2023;S0022-3913(23)00177-4.

250. Gadicherla S, Pentapati K-C, Rustaqi N, Singh A, Smriti K. Diagnostic Accuracy of Ultrasonography for the Assessment of Maxillofacial Fractures: A Meta-analysis. J Int Soc Prev Community Dent. 2021;11:503–9.

251. De Luca Canto G, Singh V, Major MP, Witmans M, El-Hakim H, Major PW, et al. Diagnostic capability of questionnaires and clinical examinations to assess sleep-disordered breathing in children: a systematic review and meta-analysis. J Am Dent Assoc. 2014;145:165–78.

252. Abesi F, Maleki M, Zamani M. Diagnostic performance of artificial intelligence using cone-beam computed tomography imaging of the oral and maxillofacial region: A scoping review and meta-analysis. Imaging Sci Dent. 2023;53:101–8.

253. Larocca de Geus J, Nogueira da Costa JK, Wambier LM, Maran BM, Loguercio AD, Reis A. Different anesthetics on the efficacy of inferior alveolar nerve block in patients with irreversible pulpitis: A network systematic review and meta-analysis. J Am Dent Assoc. 2020;151:87-97.e4.

254. Schwendicke F, Brouwer F, Schwendicke A, Paris S. Different materials for direct pulp capping: systematic review and meta-analysis and trial sequential analysis. Clin Oral Investig. 2016;20:1121–32.

255. Chochlidakis KM, Papaspyridakos P, Geminiani A, Chen C-J, Feng IJ, Ercoli C. Digital versus conventional impressions for fixed prosthodontics: A systematic review and meta-analysis. J Prosthet Dent. 2016;116:184-190.e12.

256. Atieh MA, Fadhul I, Shah M, Hannawi H, Alsabeeha NHM. Diode Laser as an Adjunctive Treatment for Peri-implant Mucositis: A Systematic Review and Meta-analysis. Int Dent J. 2022;72:735–45.

257. Pilcher L, Pahlke S, Urquhart O, O’Brien KK, Dhar V, Fontana M, et al. Direct materials for restoring caries lesions: Systematic review and meta-analysis-a report of the American Dental Association Council on Scientific Affairs. J Am Dent Assoc. 2023;154:e1–98.

258. Schwendicke F, Göstemeyer G, Blunck U, Paris S, Hsu L-Y, Tu Y-K. Directly Placed Restorative Materials: Review and Network Meta-analysis. J Dent Res. 2016;95:613–22.

259. Li J, Zhang Z, Han N. Diverse therapies for disc displacement of temporomandibular joint: a systematic review and network meta-analysis. Br J Oral Maxillofac Surg. 2022;60:1012–22.

260. Garcia PP, Wambier LM, de Geus JL, da Cunha LF, Correr GM, Gonzaga CC. Do anterior and posterior teeth treated with post-and-core restorations have similar failure rates? A systematic review and meta-analysis. J Prosthet Dent. 2019;121:887-894.e4.

261. Ata-Ali J, Ata-Ali F, Ata-Ali F. Do antibiotics decrease implant failure and postoperative infections? A systematic review and meta-analysis. Int J Oral Maxillofac Surg. 2014;43:68–74.

262. Falci SGM, Galvão EL, de Souza GM, Fernandes IA, Souza MRF, Al-Moraissi EA. Do antibiotics prevent infection after third molar surgery? A network meta-analysis. Int J Oral Maxillofac Surg. 2022;51:1226–36.

263. Bellinaso MD, Soares FZM, Rocha R de O. Do bulk-fill resins decrease the restorative time in posterior teeth? A systematic review and meta-analysis of in vitro studies. J Investig Clin Dent. 2019;10:e12463.

264. Lemos CAA, de Oliveira AS, Faé DS, Oliveira HFFE, Del Rei Daltro Rosa CD, Bento VAA, et al. Do dental implants placed in patients with osteoporosis have higher risks of failure and marginal bone loss compared to those in healthy patients? A systematic review with meta-analysis. Clin Oral Investig. 2023;27:2483–93.

265. Monje A, Fu J-H, Chan H-L, Suarez F, Galindo-Moreno P, Catena A, et al. Do implant length and width matter for short dental implants (<10 mm)? A meta-analysis of prospective studies. J Periodontol. 2013;84:1783–91.

266. Al-Moraissi EA, Perez D, Ellis E. Do patients with malocclusion have a higher prevalence of temporomandibular disorders than controls both before and after orthognathic surgery? A systematic review and meta-analysis. J Craniomaxillofac Surg. 2017;45:1716–23.

267. Sayardoust S, Johansson A, Jönsson D. Do Probiotics Cause a Shift in the Microbiota of Dental Implants-A Systematic Review and Meta-Analysis. Front Cell Infect Microbiol. 2022;12:823985.

268. Higaki N, Goto T, Ishida Y, Watanabe M, Tomotake Y, Ichikawa T. Do sensation differences exist between dental implants and natural teeth?: a meta-analysis. Clin Oral Implants Res. 2014;25:1307–10.

269. Tolentino da Rosa de Souza P, Binhame Albini Martini M, Reis Azevedo-Alanis L. Do short implants have similar survival rates compared to standard implants in posterior single crown?: A systematic review and meta-analysis. Clin Implant Dent Relat Res. 2018;20:890–901.

270. Correia A, Bresciani E, Borges AB, Pereira DM, Maia LC, Caneppele T. Do Tooth- and Cavity-related Aspects of Noncarious Cervical Lesions Affect the Retention of Resin Composite Restorations in Adults? A Systematic Review and Meta-analysis. Oper Dent. 2020;45:E124–40.

271. Turchiello RZ, Pedrotti D, Braga MM, Rocha RO, Rodrigues JA, Lenzi TL. Do undergraduate dental students perform well detecting and staging caries and assessing activity by visual examination? A systematic review and meta-analysis. Int J Paediatr Dent. 2019;29:281–93.

272. Sales PH da H, Barros AWP, Oliveira-Neto OB de, de Lima FJC, Carvalho A de AT, Leão JC. Do zirconia dental implants present better clinical results than titanium dental implants? A systematic review and meta-analysis. J Stomatol Oral Maxillofac Surg. 2023;124:101324.

273. Al-Bermani ASA, Quigley NP, Ha WN. Do zirconia single-retainer resin-bonded fixed dental prostheses present a viable treatment option for the replacement of missing anterior teeth? A systematic review and meta-analysis. J Prosthet Dent. 2023;130:533–42.

274. Shi Q, Xu J, Huo N, Cai C, Liu H. Does a higher glycemic level lead to a higher rate of dental implant failure?: A meta-analysis. J Am Dent Assoc. 2016;147:875–81.

275. Romualdo PC, de Oliveira KMH, Nemezio MA, Küchler EC, Silva RAB, Nelson-Filho P, et al. Does apical negative pressure prevent the apical extrusion of debris and irrigant compared with conventional irrigation? A systematic review and meta-analysis. Aust Endod J. 2017;43:129–37.

276. Simon AK, Bhumika TV, Nair NS. Does atraumatic restorative treatment reduce dental anxiety in children? A systematic review and meta-analysis. Eur J Dent. 2015;9:304–9.

277. Canullo L, Laino L, Longo F, Filetici P, D’Onofrio I, Troiano G. Does Chlorhexidine Prevent Complications in Extractive, Periodontal, and Implant Surgery? A Systematic Review and Meta-analysis with Trial Sequential Analysis. Int J Oral Maxillofac Implants. 2020;35:1149–58.

278. Tan SJ, Baharin B, Nabil S, Mohd N, Zhu Y. DOES GLYCEMIC CONTROL HAVE A DOSE-RESPONSE RELATIONSHIP WITH IMPLANT OUTCOMES? A COMPREHENSIVE SYSTEMATIC REVIEW AND META-ANALYSIS. J Evid Based Dent Pract. 2021;21:101543.

279. Sivakumar I, Arunachalam S, Choudhary S, Buzayan MM. Does HIV infection affect the survival of dental implants? A systematic review and meta-analysis. J Prosthet Dent. 2021;125:862–9.

280. Firoozi P, Souza MRF, de Souza GM, Fernandes IA, Galvão EL, Falci SGM. Does kinesio taping reduce pain, swelling, and trismus after mandibular third molar surgery? A systematic review and meta-analysis. Oral Maxillofac Surg. 2022;26:535–53.

281. Ye X, Gu Y, Bai Y, Xia S, Zhang Y, Lou Y, et al. Does Low-Magnitude High-Frequency Vibration (LMHFV) Worth for Clinical Trial on Dental Implant? A Systematic Review and Meta-Analysis on Animal Studies. Front Bioeng Biotechnol. 2021;9:626892.

282. Shamszadeh S, Shirvani A, Asgary S. Does occlusal reduction reduce post-endodontic pain? A systematic review and meta-analysis. J Oral Rehabil. 2020;47:528–35.

283. Nunes GP, Pirovani BO, Nunes LP, Silva ANA, Morábito MJSD, Nunes-Júnior NA, et al. Does oral lichen planus aggravate the state of periodontal disease? A systematic review and meta-analysis. Clin Oral Investig. 2022;26:3357–71.

284. Javidi H, Vettore M, Benson PE. Does orthodontic treatment before the age of 18 years improve oral health-related quality of life? A systematic review and meta-analysis. Am J Orthod Dentofacial Orthop. 2017;151:644–55.

285. Kamal AT, Malik DES, Fida M, Sukhia RH. Does periodontally accelerated osteogenic orthodontics improve orthodontic treatment outcome? A systematic review and meta-analysis. Int Orthod. 2019;17:193–201.

286. Sendyk DI, Chrcanovic BR, Albrektsson T, Wennerberg A, Zindel Deboni MC. Does Surgical Experience Influence Implant Survival Rate? A Systematic Review and Meta-Analysis. Int J Prosthodont. 2017;30:341–7.

287. Abdalla RIB, Alqutaibi AY, Kaddah A. Does the adjunctive use of platelet-rich plasma to bone graft during sinus augmentation reduce implant failure and complication? Systematic review and meta-analysis. Quintessence Int. 2018;49:139–46.

288. Yan M-D, Ou Y-J, Lin Y-J, Liu R-M, Fang Y, Wu W-L, et al. Does the incorporation of strontium into calcium phosphate improve bone repair? A meta-analysis. BMC Oral Health. 2022;22:62.

289. Silva Lima Mendes DT, Leite Matos GR, Stwart de Araújo Souza SA, Souza Silva Macedo MC, Tavares DDS, Resende CX. Does the incorporation of zinc into TiO2 on titanium surfaces increase bactericidal activity? A systematic review and meta-analysis. J Prosthet Dent. 2022;S0022-3913(22)00328-6.

290. Freitas da Silva EV, Dos Santos DM, Sonego MV, de Luna Gomes JM, Pellizzer EP, Goiato MC. Does the Presence of a Cantilever Influence the Survival and Success of Partial Implant-Supported Dental Prostheses? Systematic Review and Meta-Analysis. Int J Oral Maxillofac Implants. 2018;33:815–23.

291. Sivakumar I, Arunachalam S, Mahmoud Buzayan M, Sharan J. Does the use of platelet-rich plasma in sinus augmentation improve the survival of dental implants? A systematic review and meta-analysis. J Oral Biol Craniofac Res. 2023;13:57–66.

292. Pinto KP, Ferreira CM, Maia LC, Sassone LM, Fidalgo TKS, Silva EJNL. Does tobacco smoking predispose to apical periodontitis and endodontic treatment need? A systematic review and meta-analysis. Int Endod J. 2020;53:1068–83.

293. Wazwaz F, Seehra J, Carpenter GH, Papageorgiou SN, Cobourne MT. Duration of canine retraction with fixed appliances: A systematic review and meta-analysis. Am J Orthod Dentofacial Orthop. 2023;163:154–72.

294. Abbing A, Koretsi V, Eliades T, Papageorgiou SN. Duration of orthodontic treatment with fixed appliances in adolescents and adults: a systematic review with meta-analysis. Prog Orthod. 2020;21:37.

295. Wazwaz F, Seehra J, Carpenter GH, Ireland AJ, Papageorgiou SN, Cobourne MT. Duration of tooth alignment with fixed appliances: A systematic review and meta-analysis. Am J Orthod Dentofacial Orthop. 2022;161:20–36.

296. Pellegrino G, Ferri A, Del Fabbro M, Prati C, Gandolfi MG, Marchetti C. Dynamic Navigation in Implant Dentistry: A Systematic Review and Meta-analysis. Int J Oral Maxillofac Implants. 2021;36:e121–40.

297. Troiano G, Lo Russo L, Canullo L, Ciavarella D, Lo Muzio L, Laino L. Early and late implant failure of submerged versus non-submerged implant healing: A systematic review, meta-analysis and trial sequential analysis. J Clin Periodontol. 2018;45:613–23.

298. Caillet J, Pereira B, Courtot R, Barthélémy I, Devoize L, Depeyre A. Ectopic third mandibular molar: evaluation of surgical practices and meta-analysis. Clin Oral Investig. 2021;25:4781–99.

299. Aldhohrah T, Mashrah MA, Wang Y. Effect of 2-implant mandibular overdenture with different attachments and loading protocols on peri-implant health and prosthetic complications: A systematic review and network meta-analysis. J Prosthet Dent. 2022;127:832–44.

300. Schimmel M, Srinivasan M, McKenna G, Müller F. Effect of advanced age and/or systemic medical conditions on dental implant survival: A systematic review and meta-analysis. Clin Oral Implants Res. 2018;29 Suppl 16:311–30.

301. Avila-Ortiz G, Elangovan S, Kramer KWO, Blanchette D, Dawson DV. Effect of alveolar ridge preservation after tooth extraction: a systematic review and meta-analysis. J Dent Res. 2014;93:950–8.

302. Avila-Ortiz G, Chambrone L, Vignoletti F. Effect of alveolar ridge preservation interventions following tooth extraction: A systematic review and meta-analysis. J Clin Periodontol. 2019;46 Suppl 21:195–223.

303. McKenna GJ, Gjengedal H, Harkin J, Holland N, Moore C, Srinivasan M. EFFECT OF AUTOGENOUS BONE GRAFT SITE ON DENTAL IMPLANT SURVIVAL AND DONOR SITE COMPLICATIONS: A SYSTEMATIC REVIEW AND META-ANALYSIS. J Evid Based Dent Pract. 2022;22:101731.

304. Wehner C, Lettner S, Moritz A, Andrukhov O, Rausch-Fan X. Effect of bisphosphonate treatment of titanium surfaces on alkaline phosphatase activity in osteoblasts: a systematic review and meta-analysis. BMC Oral Health. 2020;20:125.

305. Savitha S, Sharma S, Kumar V, Chawla A, Vanamail P, Logani A. Effect of body temperature on the cyclic fatigue resistance of the nickel-titanium endodontic instruments: A systematic review and meta-analysis of in vitro studies. J Conserv Dent. 2022;25:338–46.

306. Sivaramakrishnan G, Sridharan K. Effect of clonidine on the efficacy of lignocaine local anesthesia in dentistry: A systematic review and meta-analysis of randomized, controlled trials. J Investig Clin Dent. 2018;9:e12296.

307. Zarow M, Dominiak M, Szczeklik K, Hardan L, Bourgi R, Cuevas-Suárez CE, et al. Effect of Composite Core Materials on Fracture Resistance of Endodontically Treated Teeth: A Systematic Review and Meta-Analysis of In Vitro Studies. Polymers (Basel). 2021;13:2251.

308. Zubizarreta-Macho Á, Valle Castaño S, Montiel-Company JM, Mena-Álvarez J. Effect of Computer-Aided Navigation Techniques on the Accuracy of Endodontic Access Cavities: A Systematic Review and Meta-Analysis. Biology (Basel). 2021;10:212.

309. Pourabbas R, Ghahramani N, Sadighi M, Pournaghi Azar F, Ghojazadeh M. Effect of conscious sedation use on anxiety reduction, and patient and surgeon satisfaction in dental implant surgeries: A systematic review and meta-analysis. Dent Med Probl. 2022;59:143–9.

310. Alam MK, Abutayyem H, Kanwal B, Alswairki HJ. Effect of COVID-19 on orthodontic treatment/practice- A systematic review and meta-analysis. J Orthod Sci. 2023;12:26.

311. Dank A, Aartman IHA, Wismeijer D, Tahmaseb A. Effect of dental implant surface roughness in patients with a history of periodontal disease: a systematic review and meta-analysis. Int J Implant Dent. 2019;5:12.

312. Tavares S, Pintor A, Mourão CF de AB, Magno M, Montemezzi P, Sacco R, et al. Effect of Different Root Canal Irrigant Solutions on the Release of Dentin-Growth Factors: A Systematic Review and Meta-Analysis. Materials (Basel). 2021;14:5829.

313. Ahmed A, Fida M, Habib S, Javed F, Ali US. Effect of Direct versus Indirect Bonding Technique on the Failure Rate of Mandibular Fixed Retainer-A Systematic Review and Meta-Analysis. Int Orthod. 2021;19:539–47.

314. Chen Y-W, Lee C-T, Hum L, Chuang S-K. Effect of flap design on periodontal healing after impacted third molar extraction: a systematic review and meta-analysis. Int J Oral Maxillofac Surg. 2017;46:363–72.

315. Helmy MHE-D, Alqutaibi AY, El-Ella AA, Shawky AF. Effect of implant loading protocols on failure and marginal bone loss with unsplinted two-implant-supported mandibular overdentures: systematic review and meta-analysis. Int J Oral Maxillofac Surg. 2018;47:642–50.

316. Rahul M, Lokade A, Tewari N, Mathur V, Agarwal D, Goel S, et al. Effect of Intracanal Scaffolds on the Success Outcomes of Regenerative Endodontic Therapy - A Systematic Review and Network Meta-analysis. J Endod. 2023;49:110–28.

317. Koodaryan R, Hafezeqoran A. Effect of laser-microtexturing on bone and soft tissue attachments to dental implants: A systematic review and meta-analysis. J Dent Res Dent Clin Dent Prospects. 2021;15:290–6.

318. Lyris V, Millen C, Besi E, Pace-Balzan A. Effect of leukocyte and platelet rich fibrin (L-PRF) on stability of dental implants. A systematic review and meta-analysis. Br J Oral Maxillofac Surg. 2021;59:1130–9.

319. Kc K, Bhattarai BP, Shrestha S, Shrestha B, Shrestha M. EFFECT OF LOCALLY DELIVERED BISPHOSPHONATES ON ALVEOLAR BONE: A SYSTEMATIC REVIEW AND META-ANALYSIS. J Evid Based Dent Pract. 2021;21:101580.

320. Garcia J, Dodge A, Luepke P, Wang H-L, Kapila Y, Lin G-H. Effect of membrane exposure on guided bone regeneration: A systematic review and meta-analysis. Clin Oral Implants Res. 2018;29:328–38.

321. Nguyen D, Nagendrababu V, Pulikkotil SJ, Rossi-Fedele G. Effect of occlusal reduction on postendodontic pain: A systematic review and meta-analysis of randomised clinical trials. Aust Endod J. 2020;46:282–94.

322. Pulikkotil SJ, Nagendrababu V, Veettil SK, Jinatongthai P, Setzer FC. Effect of oral premedication on the anaesthetic efficacy of inferior alveolar nerve block in patients with irreversible pulpitis - A systematic review and network meta-analysis of randomized controlled trials. Int Endod J. 2018;51:989–1004.

323. Papageorgiou SN, Xavier GM, Cobourne MT, Eliades T. Effect of orthodontic treatment on the subgingival microbiota: A systematic review and meta-analysis. Orthod Craniofac Res. 2018;21:175–85.

324. Bucci R, Rongo R, Zunino B, Michelotti A, Bucci P, Alessandri-Bonetti G, et al. Effect of orthopedic and functional orthodontic treatment in children with obstructive sleep apnea: A systematic review and meta-analysis. Sleep Med Rev. 2023;67:101730.

325. Havakeshian G, Koretsi V, Eliades T, Papageorgiou SN. Effect of Orthopedic Treatment for Class III Malocclusion on Upper Airways: A Systematic Review and Meta-Analysis. J Clin Med. 2020;9:3015.

326. Ebrahimi P, Hadilou M, Naserneysari F, Dolatabadi A, Tarzemany R, Vahed N, et al. Effect of photobiomodulation in secondary intention gingival wound healing-a systematic review and meta-analysis. BMC Oral Health. 2021;21:258.

327. Tabassum S, Raj SC, Rath H, Mishra AK, Mohapatra A, Patnaik K. Effect of platelet rich fibrin on stability of dental implants: A systematic review and meta-analysis. Int J Health Sci (Qassim). 2022;16:58–68.

328. Suneelkumar C, Subha A, Gogala D. Effect of Preoperative Corticosteroids in Patients with Symptomatic Pulpitis on Postoperative Pain after Single-visit Root Canal Treatment: A Systematic Review and Meta-analysis. J Endod. 2018;44:1347–54.

329. Vinnakota DN, Kamatham R. Effect of proton pump inhibitors on dental implants: A systematic review and meta-analysis. J Indian Prosthodont Soc. 2020;20:228–36.

330. da Silveira MT, Batista SM, Mamede Veloso SR, de Oliveira NG, de Vasconcelos Carvalho M, de Melo Monteiro GQ. Effect of Reciprocating and Rotary Systems on Postoperative Pain: A Systematic Review and Meta-Analysis. Iran Endod J. 2021;16:1–16.

331. Santamaría-Villegas A, Manrique-Hernandez R, Alvarez-Varela E, Restrepo-Serna C. Effect of removable functional appliances on mandibular length in patients with class II with retrognathism: systematic review and meta-analysis. BMC Oral Health. 2017;17:52.

332. Koide Y, Kataoka Y, Hasegawa T, Ota E, Noma H. Effect of systemic bisphosphonate administration on patients with periodontal disease: a systematic review and meta-analysis protocol. BMJ Open. 2022;12:e057768.

333. Olmedo-Hernández OL, Mota-Rodríguez AN, Torres-Rosas R, Argueta-Figueroa L. Effect of the photobiomodulation for acceleration of the orthodontic tooth movement: a systematic review and meta-analysis. Lasers Med Sci. 2022;37:2323–41.

334. Vazouras K, de Souza AB, Gholami H, Papaspyridakos P, Pagni S, Weber H-P. Effect of time in function on the predictability of short dental implants (≤6 mm): A meta-analysis. J Oral Rehabil. 2020;47:403–15.

335. Kumar S, Tadakamadla J, Johnson NW. Effect of Toothbrushing Frequency on Incidence and Increment of Dental Caries: A Systematic Review and Meta-Analysis. J Dent Res. 2016;95:1230–6.

336. Akram Z, Javed F, Vohra F. Effect of waterpipe smoking on peri-implant health: A systematic review and meta-analysis. J Investig Clin Dent. 2019;10:e12403.

337. Hafezeqoran A, Koodaryan R. Effect of Zirconia Dental Implant Surfaces on Bone Integration: A Systematic Review and Meta-Analysis. Biomed Res Int. 2017;2017:9246721.

338. Fu T, Liu S, Zhao H, Cao M, Zhang R. Effectiveness and Safety of Minimally Invasive Orthodontic Tooth Movement Acceleration: A Systematic Review and Meta-analysis. J Dent Res. 2019;98:1469–79.

339. Santos GM, Pacheco RL, Bussadori SK, Santos EM, Riera R, de Oliveira Cruz Latorraca C, et al. Effectiveness and Safety of Ozone Therapy in Dental Caries Treatment: Systematic Review and Meta-analysis. J Evid Based Dent Pract. 2020;20:101472.

340. Moraschini V, da Costa LS, Dos Santos GO. Effectiveness for dentin hypersensitivity treatment of non-carious cervical lesions: a meta-analysis. Clin Oral Investig. 2018;22:617–31.

341. Rossi MT, de Oliveira MN, Vidigal MTC, de Andrade Vieira W, Figueiredo CE, Blumenberg C, et al. Effectiveness of anesthetic solutions for pain control in lower third molar extraction surgeries: a systematic review of randomized clinical trials with network meta-analysis. Clin Oral Investig. 2021;25:1–22.

342. Panda S, Mishra L, Arbildo-Vega HI, Lapinska B, Lukomska-Szymanska M, Khijmatgar S, et al. Effectiveness of Autologous Platelet Concentrates in Management of Young Immature Necrotic Permanent Teeth-A Systematic Review and Meta-Analysis. Cells. 2020;9:2241.

343. López-Valverde N, Aragoneses J, López-Valverde A, Quispe-López N, Rodríguez C, Aragoneses JM. Effectiveness of biomolecule-based bioactive surfaces, on os-seointegration of titanium dental implants: A systematic review and meta-analysis of in vivo studies. Front Bioeng Biotechnol. 2022;10:986112.

344. Dong H, Guo C, Zhou L, Zhao J, Wu X, Zhang X, et al. Effectiveness of case-based learning in Chinese dental education: a systematic review and meta-analysis. BMJ Open. 2022;12:e048497.

345. Chao Y-C, Chen P-H, Su W-S, Yeh H-W, Su C-C, Wu Y-C, et al. Effectiveness of different root-end filling materials in modern surgical endodontic treatment: A systematic review and network meta-analysis. J Dent Sci. 2022;17:1731–43.

346. de la Puente Dongo JL, Grillo R, Bueno BU, Teixeira RG. Effectiveness of Honey in the Treatment and Prevention of Alveolar Osteitis: Systematic Review and Meta-analysis. J Maxillofac Oral Surg. 2022;21:1007–14.

347. Shah DN, Chauhan CJ, Solanki JS. Effectiveness of hyperbaric oxygen therapy in irradiated maxillofacial dental implant patients: A systematic review with meta-analysis. J Indian Prosthodont Soc. 2017;17:109–19.

348. Carra MC, Rangé H, Swerts P-J, Tuand K, Vandamme K, Bouchard P. Effectiveness of implant-supported fixed partial denture in patients with history of periodontitis: A systematic review and meta-analysis. J Clin Periodontol. 2022;49 Suppl 24:208–23.

349. Behdin S, Monje A, Lin G-H, Edwards B, Othman A, Wang H-L. Effectiveness of Laser Application for Periodontal Surgical Therapy: Systematic Review and Meta-Analysis. J Periodontol. 2015;86:1352–63.

350. Moreno-Drada JA, Abreu LG, Lino PA, Parreiras Martins MA, Pordeus IA, Nogueira Guimarães de Abreu MH. Effectiveness of local hemostatic to prevent bleeding in dental patients on anticoagulation: A systematic review and network meta-analysis. J Craniomaxillofac Surg. 2021;49:570–83.

351. Bakdach WMM, Hadad R. Effectiveness of low-level laser therapy in accelerating the orthodontic tooth movement: A systematic review and meta-analysis. Dent Med Probl. 2020;57:73–94.

352. Alfawal AMH, Hajeer MY, Ajaj MA, Hamadah O, Brad B. Effectiveness of minimally invasive surgical procedures in the acceleration of tooth movement: a systematic review and meta-analysis. Prog Orthod. 2016;17:33.

353. Siddhisaributr P, Khlongwanitchakul K, Anuwongnukroh N, Manopatanakul S, Viwattanatipa N. Effectiveness of miniscrew assisted rapid palatal expansion using cone beam computed tomography: A systematic review and meta-analysis. Korean J Orthod. 2022;52:182–200.

354. Gkantidis N, Mistakidis I, Kouskoura T, Pandis N. Effectiveness of non-conventional methods for accelerated orthodontic tooth movement: a systematic review and meta-analysis. J Dent. 2014;42:1300–19.

355. Zhang M, Liu Y, Liu Y, Yu F, Yan S, Chen L, et al. Effectiveness of oral appliances versus continuous positive airway pressure in treatment of OSA patients: An updated meta-analysis. Cranio. 2019;37:347–64.

356. Mheissen S, Daraqel B, Alzoubi EE, Khan H. Effectiveness of platelet-rich concentrates on the rate of orthodontic tooth movement: a systematic review and meta-analysis. Eur J Orthod. 2023;45:196–207.

357. Pocaterra A, Caruso S, Bernardi S, Scagnoli L, Continenza MA, Gatto R. Effectiveness of platelet-rich plasma as an adjunctive material to bone graft: a systematic review and meta-analysis of randomized controlled clinical trials. Int J Oral Maxillofac Surg. 2016;45:1027–34.

358. Sardana D, Zhang J, Ekambaram M, Yang Y, McGrath CP, Yiu CKY. Effectiveness of professional fluorides against enamel white spot lesions during fixed orthodontic treatment: A systematic review and meta-analysis. J Dent. 2019;82:1–10.

359. Tomson PL, Vilela Bastos J, Jacimovic J, Jakovljevic A, Pulikkotil SJ, Nagendrababu V. Effectiveness of pulpotomy compared with root canal treatment in managing non-traumatic pulpitis associated with spontaneous pain: A systematic review and meta-analysis. Int Endod J. 2023;56 Suppl 3:355–69.

360. Hu H, Feng C, Jiang Z, Wang L, Shrestha S, Yan J, et al. Effectiveness of remineralizing agents in the prevention and reversal of orthodontically induced white spot lesions: a systematic review and network meta-analysis. Clin Oral Investig. 2020;24:4153–67.

361. Calheiros-Lobo MJ, Vieira T, Carbas R, da Silva LFM, Pinho T. Effectiveness of Self-Adhesive Resin Luting Cement in CAD-CAM Blocks-A Systematic Review and Meta-Analysis. Materials (Basel). 2023;16:2996.

362. Antezack A, Ohanessian R, Sadowski C, Faure-Brac M, Brincat A, Etchecopar-Etchart D, et al. Effectiveness of surgical root coverage on dentin hypersensitivity: A systematic review and meta-analysis. J Clin Periodontol. 2022;49:840–51.

363. Nagendrababu V, Pulikkotil SJ, Sultan OS, Jayaraman J, Soh JA, Dummer PMH. Effectiveness of technology-enhanced learning in Endodontic education: a systematic review and meta-analysis. Int Endod J. 2019;52:181–92.

364. Vande A, Sanyal PK, Nilesh K. Effectiveness of the photobiomodulation therapy using low-level laser around dental implants: A systematic review and meta-analysis. Dent Med Probl. 2022;59:281–9.

365. Elmotaleb MAA, Elnamrawy MM, Sharaby F, Elbeialy AR, ElDakroury A. Effectiveness of using a Vibrating Device in Accelerating Orthodontic Tooth Movement: A Systematic Review and Meta-Analysis. J Int Soc Prev Community Dent. 2019;9:5–12.

366. Urban IA, Montero E, Monje A, Sanz-Sánchez I. Effectiveness of vertical ridge augmentation interventions: A systematic review and meta-analysis. J Clin Periodontol. 2019;46 Suppl 21:319–39.

367. Yan X, Yan Y, Cao M, Xie W, O’Connor S, Lee JJ, et al. Effectiveness of virtual reality distraction interventions to reduce dental anxiety in paediatric patients: A systematic review and meta-analysis. J Dent. 2023;132:104455.

368. da Rosa G, Maran BM, Schmitt VL, Loguercio AD, Reis A, Naufel FS. Effectiveness of Whitening Strips Use Compared With Supervised Dental Bleaching: A Systematic Review and Meta-analysis. Oper Dent. 2020;45:E289–307.

369. Zhou J, Liu T, Guo L. Effectiveness of XP-Endo Finisher and passive ultrasonic irrigation on intracanal medicament removal from root canals: a systematic review and meta-analysis. BMC Oral Health. 2021;21:294.

370. Pesce P, Del Fabbro M, Menini M, De Giovanni E, Annunziata M, Khijmatgar S, et al. Effects of abutment materials on peri-implant soft tissue health and stability: A network meta-analysis. J Prosthodont Res. 2023;67:506–17.

371. Shamszadeh S, Asgary S, Shirvani A, Eghbal MJ. Effects of antibiotic administration on post-operative endodontic symptoms in patients with pulpal necrosis: A systematic review and meta-analysis. J Oral Rehabil. 2021;48:332–42.

372. Bi Y, Aldhohrah T, Mashrah MA, Su Y, Yang Z, Guo X, et al. Effects of attachment type and number of dental implants supporting mandibular overdenture on peri-implant health: A systematic review and network meta-analysis. J Prosthodont Res. 2022;66:357–73.

373. Lv X, Zou L, Zhang X, Zhang X, Lai H, Shi J. Effects of diabetes/hyperglycemia on peri-implant biomarkers and clinical and radiographic outcomes in patients with dental implant restorations: A systematic review and meta-analysis. Clin Oral Implants Res. 2022;33:1183–98.

374. Yu C, Li Q-Q, Zhang R-Z, Chen S-L, Xia F-F, Zhou Z. Effects of different surgical treatments on children with ankyloglossia: protocol for a systematic review and meta-analysis. BMJ Open. 2022;12:e065653.

375. Shamszadeh S, Shirvani A, Torabzadeh H, Asgary S. Effects of Growth Factors on Cellular Activities of Dental Stem Cells: A Systematic Review and Meta-analysis (Part I). Curr Stem Cell Res Ther. 2022;

376. Dioguardi M, Sovereto D, Aiuto R, Laino L, Illuzzi G, Laneve E, et al. Effects of Hot Sterilization on Torsional Properties of Endodontic Instruments: Systematic Review with Meta-Analysis. Materials (Basel). 2019;12:2190.

377. Alenezi A, Chrcanovic B, Wennerberg A. Effects of Local Drug and Chemical Compound Delivery on Bone Regeneration Around Dental Implants in Animal Models: A Systematic Review and Meta-Analysis. Int J Oral Maxillofac Implants. 2018;33:e1–18.

378. Dos Santos CCO, Mecenas P, de Castro Aragón MLS, Normando D. Effects of micro-osteoperforations performed with Propel system on tooth movement, pain/quality of life, anchorage loss, and root resorption: a systematic review and meta-analysis. Prog Orthod. 2020;21:27.

379. Sanz-Martín I, Sanz-Sánchez I, Carrillo de Albornoz A, Figuero E, Sanz M. Effects of modified abutment characteristics on peri-implant soft tissue health: A systematic review and meta-analysis. Clin Oral Implants Res. 2018;29:118–29.

380. Lemos C a. A, Mello CC, dos Santos DM, Verri FR, Goiato MC, Pellizzer EP. Effects of platelet-rich plasma in association with bone grafts in maxillary sinus augmentation: a systematic review and meta-analysis. Int J Oral Maxillofac Surg. 2016;45:517–25.

381. Yu X, Teng F, Zhao A, Wu Y, Yu D. EFFECTS OF POST-EXTRACTION ALVEOLAR RIDGE PRESERVATION VERSUS IMMEDIATE IMPLANT PLACEMENT: A SYSTEMATIC REVIEW AND META-ANALYSIS. J Evid Based Dent Pract. 2022;22:101734.

382. Alenezi A, Chrcanovic B. Effects of the local administration of antibiotics on bone formation on implant surface in animal models: A systematic review and meta-analysis. Jpn Dent Sci Rev. 2020;56:177–83.

383. Su N, Wang H, Zhang S, Liao S, Yang S, Huang Y. Efficacy and safety of bupivacaine versus lidocaine in dental treatments: a meta-analysis of randomised controlled trials. Int Dent J. 2014;64:34–45.

384. MacDonald L, Zanjir M, Laghapour Lighvan N, da Costa BR, Suri S, Azarpazhooh A. Efficacy and safety of different interventions to accelerate maxillary canine retraction following premolar extraction: A systematic review and network meta-analysis. Orthod Craniofac Res. 2021;24:17–38.

385. Su N, Liu Y, Yang X, Shi Z, Huang Y. Efficacy and safety of mepivacaine compared with lidocaine in local anaesthesia in dentistry: a meta-analysis of randomised controlled trials. Int Dent J. 2014;64:96–107.

386. Karlsson K, Trullenque-Eriksson A, Tomasi C, Derks J. Efficacy of access flap and pocket elimination procedures in the management of peri-implantitis: A systematic review and meta-analysis. J Clin Periodontol. 2023;50 Suppl 26:244–84.

387. Yaghini J, Mogharehabed A, Feizi A, Afshari Z. Efficacy of Acellular Dermal Matrix in Soft Tissue Augmentation Around Dental Implants: A Systematic Review and Meta-Analysis. J Oral Implantol. 2023;49:197–205.

388. Ma KS-K, Wang L-T, Blatz MB. Efficacy of adhesive strategies for restorative dentistry: A systematic review and network meta-analysis of double-blind randomized controlled trials over 12 months of follow-up. J Prosthodont Res. 2023;67:35–44.

389. Ye M, Liu W, Cheng S, Yan L. Efficacy of Adjunctive Chlorhexidine in non-surgical treatment of Peri-Implantitis/Peri-Implant Mucositis: An updated systematic review and meta-analysis. Pak J Med Sci. 2023;39:595–604.

390. López-Valverde N, López-Valverde A, Blanco-Rueda JA. Efficacy of adjuvant metronidazole therapy on peri-implantitis: a systematic review and meta-analysis of randomized clinical studies. Front Cell Infect Microbiol. 2023;13:1149055.

391. Yun K-I, Choi H, Wright RF, Ahn HS, Chang BM, Kim HJ. Efficacy of Alveolar Vertical Distraction Osteogenesis and Autogenous Bone Grafting for Dental Implants: Systematic Review and Meta-Analysis. Int J Oral Maxillofac Implants. 2016;31:26–36.

392. Shamszadeh S, Shirvani A, Eghbal MJ, Asgary S. Efficacy of Corticosteroids on Postoperative Endodontic Pain: A Systematic Review and Meta-analysis. J Endod. 2018;44:1057–65.

393. Khater AGA, Al-Hamed FS, Safwat EM, Hamouda MMA, Shehata MSA, Scarano A. EFFICACY OF HEMOSTATIC AGENTS IN ENDODONTIC SURGERY: A SYSTEMATIC REVIEW AND NETWORK META-ANALYSIS. J Evid Based Dent Pract. 2021;21:101540.

394. Kommuri K, Michelogiannakis D, Barmak BA, Rossouw PE, Javed F. Efficacy of herbal- versus chlorhexidine-based mouthwashes towards oral hygiene maintenance in patients undergoing fixed orthodontic therapy: A systematic review and meta-analysis. Int J Dent Hyg. 2022;20:100–11.

395. Wolf TG, Schläppi S, Benz CI, Campus G. Efficacy of Hypnosis on Dental Anxiety and Phobia: A Systematic Review and Meta-Analysis. Brain Sci. 2022;12:521.

396. Virdee SS, Seymour DW, Farnell D, Bhamra G, Bhakta S. Efficacy of irrigant activation techniques in removing intracanal smear layer and debris from mature permanent teeth: a systematic review and meta-analysis. Int Endod J. 2018;51:605–21.

397. Singh A, Gadicherla S, Smriti K, Pentapati KC. Efficacy of Lignocaine with Buprenorphine versus Lignocaine in the Management of Postoperative Pain after Minor Oral Surgical Procedures: A Systematic Review and Meta-analysis. J Int Soc Prev Community Dent. 2020;10:686–91.

398. Toledano M, Osorio MT, Vallecillo-Rivas M, Toledano-Osorio M, Rodríguez-Archilla A, Toledano R, et al. Efficacy of local antibiotic therapy in the treatment of peri-implantitis: A systematic review and meta-analysis. J Dent. 2021;113:103790.

399. Teoh L, McCullough M, Taing M-W. Efficacy of oxycodone for postoperative dental pain: A systematic review and meta-analysis. J Dent. 2022;125:104254.

400. Al-Hamed FS, Tawfik MA-M, Abdelfadil E, Al-Saleh MAQ. Efficacy of Platelet-Rich Fibrin After Mandibular Third Molar Extraction: A Systematic Review and Meta-Analysis. J Oral Maxillofac Surg. 2017;75:1124–35.

401. Jain A, Rai A, Singh A, Taneja S. Efficacy of preoperative antibiotics in prevention of dental implant failure: a Meta-analysis of randomized controlled trials. Oral Maxillofac Surg. 2020;24:469–75.

402. Rahate PS, Kolte RA, Kolte AP, Bodhare GH, Lathiya VN. Efficacy of simultaneous placement of dental implants in osteotome-mediated sinus floor elevation with and without bone augmentation: A systematic review and meta-analysis. J Indian Soc Periodontol. 2023;27:31–9.

403. Valles C, Vilarrasa J, Barallat L, Pascual A, Nart J. Efficacy of soft tissue augmentation procedures on tissue thickening around dental implants: A systematic review and meta-analysis. Clin Oral Implants Res. 2022;33 Suppl 23:72–99.

404. Paixão S, Rodrigues C, Grenho L, Fernandes MH. Efficacy of sonic and ultrasonic activation during endodontic treatment: a Meta-analysis of in vitro studies. Acta Odontol Scand. 2022;80:588–95.

405. Cheng J, Chen L, Tao X, Qiang X, Li R, Ma J, et al. Efficacy of surgical methods for peri-implantitis: a systematic review and network meta-analysis. BMC Oral Health. 2023;23:227.

406. Mahardawi B, Jiaranuchart S, Tompkins KA, Pimkhaokham A. Efficacy of the autogenous dentin graft for implant placement: a systematic review and meta-analysis of randomized controlled trials. Int J Oral Maxillofac Surg. 2023;52:604–12.

407. Tavelli L, Barootchi S, Nguyen TVN, Tattan M, Ravidà A, Wang H-L. Efficacy of tunnel technique in the treatment of localized and multiple gingival recessions: A systematic review and meta-analysis. J Periodontol. 2018;89:1075–90.

408. de Almeida VL, de Andrade Gois VL, Andrade RNM, Cesar CPHAR, de Albuquerque-Junior RLC, de Mello Rode S, et al. Efficiency of low-level laser therapy within induced dental movement: A systematic review and meta-analysis. J Photochem Photobiol B. 2016;158:258–66.

409. Roca-Millan E, Domínguez-Mínger J, Schemel-Suárez M, Estrugo-Devesa A, Marí-Roig A, López-López J. Epstein-Barr Virus and Peri-Implantitis: A Systematic Review and Meta-Analysis. Viruses. 2021;13:250.

410. Fang J, Li Y, Zhang K, Zhao Z, Mei L. Escaping the Adverse Impacts of NSAIDs on Tooth Movement During Orthodontics: Current Evidence Based on a Meta-Analysis. Medicine (Baltimore). 2016;95:e3256.

411. Doğramacı EJ, Rossi-Fedele G. Establishing the association between nonnutritive sucking behavior and malocclusions: A systematic review and meta-analysis. J Am Dent Assoc. 2016;147:926-934.e6.

412. Huynh R, Peters CI, Zafar S, Peters OA. Evaluating the stress of root canal treatment in patients and dentists compared to other dental treatments: A systematic review and meta-analysis. Eur J Oral Sci. 2023;131:e12941.

413. Lagunov VL, Sun J, George R. Evaluation of biologic implant success parameters in type 2 diabetic glycemic control patients versus health patients: A meta-analysis. J Investig Clin Dent. 2019;10:e12478.

414. Lemos CAA, de Souza Batista VE, Almeida DA de F, Santiago Júnior JF, Verri FR, Pellizzer EP. Evaluation of cement-retained versus screw-retained implant-supported restorations for marginal bone loss: A systematic review and meta-analysis. J Prosthet Dent. 2016;115:419–27.

415. Javaheripour A, Saatloo MV, Vahed N, Gavgani LF, Kouhsoltani M. Evaluation of HER2/neu expression in different types of salivary gland tumors: a systematic review and meta-analysis. J Med Life. 2022;15:595–600.

416. Koodaryan R, Hafezeqoran A. Evaluation of Implant Collar Surfaces for Marginal Bone Loss: A Systematic Review and Meta-Analysis. Biomed Res Int. 2016;2016:4987526.

417. Azizi Mazreah S, Shirvani A, Azizi Mazreah H, Dianat O. Evaluation of irrigant extrusion following the use of different root canal irrigation techniques: A systematic review and meta-analysis. Aust Endod J. 2023;49:396–417.

418. Mitsea A, Palikaraki G, Karamesinis K, Vastardis H, Gizani S, Sifakakis I. Evaluation of Lateral Incisor Resorption Caused by Impacted Maxillary Canines Based on CBCT: A Systematic Review and Meta-Analysis. Children (Basel). 2022;9:1006.

419. Zhuang J, Zhao D, Wu Y, Xu C. Evaluation of Outcomes of Dental Implants Inserted by Flapless or Flapped Procedure: A Meta-Analysis. Implant Dent. 2018;27:588–98.

420. Deng Y, Sun Y, Xu T. Evaluation of root resorption after comprehensive orthodontic treatment using cone beam computed tomography (CBCT): a meta-analysis. BMC Oral Health. 2018;18:116.

421. Londono J, Ghasemi S, Lawand G, Dashti M. Evaluation of the golden proportion in the natural dentition: A systematic review and meta-analysis. J Prosthet Dent. 2023;129:696–702.

422. Kulkarni V, Uttamani JR, Asar NV, Nares S, Tözüm TF. Evidence-Based Clinical Outcomes of Immediate and Early Loading of Short Endosseous Dental Implants: A Meta-analysis. Int J Oral Maxillofac Implants. 2021;36:59–67.

423. Antonacci D, Caponio VCA, Troiano G, Pompeo MG, Gianfreda F, Canullo L. Facial scanning technologies in the era of digital workflow: A systematic review and network meta-analysis. J Prosthodont Res. 2023;67:321–36.

424. Jiang M, Fan Y, Li KY, Lo ECM, Chu CH, Wong MCM. Factors affecting success rate of atraumatic restorative treatment (ART) restorations in children: A systematic review and meta-analysis. J Dent. 2021;104:103526.

425. Borges GA, Codello DJ, Del Rio Silva L, Dini C, Barão VAR, Mesquita MF. Factors and clinical outcomes for standard and mini-implants retaining mandibular overdentures: A systematic review and meta-analysis. J Prosthet Dent. 2023;130:677–89.

426. Nahajowski M, Hnitecka S, Antoszewska-Smith J, Rumin K, Dubowik M, Sarul M. Factors influencing an eruption of teeth associated with a dentigerous cyst: a systematic review and meta-analysis. BMC Oral Health. 2021;21:180.

427. Wu H, Shi Q, Huang Y, Chang P, Huo N, Jiang Y, et al. Failure Risk of Short Dental Implants Under Immediate Loading: A Meta-Analysis. J Prosthodont. 2021;30:569–80.

428. Badaró MM, Mendoza Marin DO, Pauletto P, Simek Vega Gonçalves TM, Porporatti AL, De Luca Canto G. Failures in Single Extra-Short Implants (≤ 6 mm): A Systematic Review and Meta-analysis. Int J Oral Maxillofac Implants. 2021;36:669–89.

429. Pauletto P, Ruales-Carrera E, Gonçalves TMSV, Philippi AG, Donos N, Mezzomo LA. Fixed and Removable Full-Arch Restorations Supported by Short (≤ 8-mm) Dental Implants In the Mandible: A Systematic Review and Meta-Analysis. Int J Oral Maxillofac Implants. 2019;34:873–85.

430. Chrcanovic BR, Albrektsson T, Wennerberg A. Flapless versus conventional flapped dental implant surgery: a meta-analysis. PLoS One. 2014;9:e100624.

431. Alhajj MN, Qi CH, Sayed ME, Johari Y, Ariffin Z. Fracture Resistance of Titanium and Fiber Dental Posts: A Systematic Review and Meta-Analysis. J Prosthodont. 2022;31:374–84.

432. Coray R, Zeltner M, Özcan M. Fracture strength of implant abutments after fatigue testing: A systematic review and a meta-analysis. J Mech Behav Biomed Mater. 2016;62:333–46.

433. Papageorgiou SN, Eliades T, Hämmerle CHF. Frequency of infraposition and missing contact points in implant-supported restorations within natural dentitions over time: A systematic review with meta-analysis. Clin Oral Implants Res. 2018;29 Suppl 18:309–25.

434. Dioguardi M, Cantore S, Scacco S, Quarta C, Sovereto D, Spirito F, et al. From Bench to Bedside in Precision Medicine: Diabetes Mellitus and Peri-Implantitis Clinical Indices with a Short-Term Follow-Up: A Systematic Review and Meta-Analysis. J Pers Med. 2022;12:235.

435. Alam MK, Abutayyem H, Kanwal B, A L Shayeb M. Future of Orthodontics-A Systematic Review and Meta-Analysis on the Emerging Trends in This Field. J Clin Med. 2023;12:532.

436. Monje A, Alcoforado G, Padial-Molina M, Suarez F, Lin G-H, Wang H-L. Generalized aggressive periodontitis as a risk factor for dental implant failure: a systematic review and meta-analysis. J Periodontol. 2014;85:1398–407.

437. Barootchi S, Tavelli L, Zucchelli G, Giannobile WV, Wang H-L. Gingival phenotype modification therapies on natural teeth: A network meta-analysis. J Periodontol. 2020;91:1386–99.

438. Bezerra IM, Brito ACM, de Sousa SA, Santiago BM, Cavalcanti YW, de Almeida L de FD. Glass ionomer cements compared with composite resin in restoration of noncarious cervical lesions: A systematic review and meta-analysis. Heliyon. 2020;6:e03969.

439. Duan D-H, Fu J-H, Qi W, Du Y, Pan J, Wang H-L. Graft-Free Maxillary Sinus Floor Elevation: A Systematic Review and Meta-Analysis. J Periodontol. 2017;88:550–64.

440. Wessing B, Lettner S, Zechner W. Guided Bone Regeneration with Collagen Membranes and Particulate Graft Materials: A Systematic Review and Meta-Analysis. Int J Oral Maxillofac Implants. 2018;33:87–100.

441. Baniulyte G, Ali K, Burns L. Guided tissue regeneration techniques involving blood-derived products in periradicular surgery: a systematic review and meta-analysis protocol. JBI Evid Synth. 2021;19:3378–83.

442. Hu S, BaniHani A, Nevitt S, Maden M, Santamaria RM, Albadri S. Hall technique for primary teeth: A systematic review and meta-analysis. Jpn Dent Sci Rev. 2022;58:286–97.

443. Starch-Jensen T, Deluiz D, Deb S, Bruun NH, Tinoco EMB. Harvesting of Autogenous Bone Graft from the Ascending Mandibular Ramus Compared with the Chin Region: a Systematic Review and Meta-Analysis Focusing on Complications and Donor Site Morbidity. J Oral Maxillofac Res. 2020;11:e1.

444. Tsai C, Raphael S, Agnew C, McDonald G, Irving M. Health promotion interventions to improve oral health of adolescents: A systematic review and meta-analysis. Community Dent Oral Epidemiol. 2020;48:549–60.

445. Wu J, Li B, Lin X. Histological outcomes of sinus augmentation for dental implants with calcium phosphate or deproteinized bovine bone: a systematic review and meta-analysis. Int J Oral Maxillofac Surg. 2016;45:1471–7.

446. Canellas JVDS, Ritto FG, Figueredo CMDS, Fischer RG, de Oliveira GP, Thole AA, et al. Histomorphometric evaluation of different grafting materials used for alveolar ridge preservation: a systematic review and network meta-analysis. Int J Oral Maxillofac Surg. 2020;49:797–810.

447. Wen X, Liu R, Li G, Deng M, Liu L, Zeng X-T, et al. History of periodontitis as a risk factor for long-term survival of dental implants: a meta-analysis. Int J Oral Maxillofac Implants. 2014;29:1271–80.

448. Rakic M, Galindo-Moreno P, Monje A, Radovanovic S, Wang H-L, Cochran D, et al. How frequent does peri-implantitis occur? A systematic review and meta-analysis. Clin Oral Investig. 2018;22:1805–16.

449. de la Cour CD, Sperling CD, Belmonte F, Syrjänen S, Kjaer SK. Human papillomavirus prevalence in oral potentially malignant disorders: Systematic review and meta-analysis. Oral Dis. 2021;27:431–8.

450. Benites Condezo AF, Araujo RZ, Koga DH, Curi MM, Cardoso CL. Hyperbaric oxygen therapy for the placement of dental implants in irradiated patients: systematic review and meta-analysis. Br J Oral Maxillofac Surg. 2021;59:625–32.

451. Eslamipour F, Motamedian SR, Bagheri F. Ibuprofen and Low-level Laser Therapy for Pain Control during Fixed Orthodontic Therapy: A Systematic Review of Randomized Controlled Trials and Meta-analysis. J Contemp Dent Pract. 2017;18:527–33.

452. In ’t Veld M, Schulten EAJM, Leusink FKJ. Immediate dental implant placement and restoration in the edentulous mandible in head and neck cancer patients: a systematic review and meta-analysis. Curr Opin Otolaryngol Head Neck Surg. 2021;29:126–37.

453. de Oliveira-Neto O-B, Lemos C -a.-A, Barbosa F-T, de Sousa-Rodrigues C-F, Camello de Lima F-J. Immediate dental implants placed into infected sites present a higher risk of failure than immediate dental implants placed into non-infected sites: Systematic review and meta-analysis. Med Oral Patol Oral Cir Bucal. 2019;24:e518–28.

454. Ragucci GM, Elnayef B, Criado-Cámara E, Del Amo FS-L, Hernández-Alfaro F. Immediate implant placement in molar extraction sockets: a systematic review and meta-analysis. Int J Implant Dent. 2020;6:40.

455. Saijeva A, Juodzbalys G. Immediate Implant Placement in Non-Infected Sockets versus Infected Sockets: a Systematic Review and Meta-Analysis. J Oral Maxillofac Res. 2020;11:e1.

456. Mello CC, Lemos C a. A, Verri FR, Dos Santos DM, Goiato MC, Pellizzer EP. Immediate implant placement into fresh extraction sockets versus delayed implants into healed sockets: A systematic review and meta-analysis. Int J Oral Maxillofac Surg. 2017;46:1162–77.

457. Chrcanovic BR, Albrektsson T, Wennerberg A. Immediate nonfunctional versus immediate functional loading and dental implant failure rates: a systematic review and meta-analysis. J Dent. 2014;42:1052–9.

458. Chen H, Zhang G, Weigl P, Gu X. Immediate placement of dental implants into infected versus noninfected sites in the esthetic zone: A systematic review and meta-analysis. J Prosthet Dent. 2018;120:658–67.

459. Atieh MA, Payne AGT, Duncan WJ, de Silva RK, Cullinan MP. Immediate placement or immediate restoration/loading of single implants for molar tooth replacement: a systematic review and meta-analysis. Int J Oral Maxillofac Implants. 2010;25:401–15.

460. Moraschini V, Porto Barboza E. Immediate versus conventional loaded single implants in the posterior mandible: a meta-analysis of randomized controlled trials. Int J Oral Maxillofac Surg. 2016;45:85–92.

461. Pigozzo MN, Rebelo da Costa T, Sesma N, Laganá DC. Immediate versus early loading of single dental implants: A systematic review and meta-analysis. J Prosthet Dent. 2018;120:25–34.

462. Chen J, Cai M, Yang J, Aldhohrah T, Wang Y. Immediate versus early or conventional loading dental implants with fixed prostheses: A systematic review and meta-analysis of randomized controlled clinical trials. J Prosthet Dent. 2019;122:516–36.

463. Ye M, Liu W, Cheng S, Yan L. Immediate vs Conventional Loading of Mandibular Overdentures: A Comprehensive Systematic Review and Meta-Analysis of Randomized Controlled Trials. J Oral Implantol. 2022;48:64–73.

464. Chrcanovic BR, Albrektsson T, Wennerberg A. Immediately loaded non-submerged versus delayed loaded submerged dental implants: a meta-analysis. Int J Oral Maxillofac Surg. 2015;44:493–506.

465. Pommer B, Frantal S, Willer J, Posch M, Watzek G, Tepper G. Impact of dental implant length on early failure rates: a meta-analysis of observational studies. J Clin Periodontol. 2011;38:856–63.

466. Li Y, Lu Z, Sun H. Impact of diabetes mellitus on the poor prognosis in patients with osseointegrated dental implants: a meta-analysis of observational studies. Biotechnol Genet Eng Rev. 2023;1–19.

467. Mazur M, Ndokaj A, Jedlinski M, Ardan R, Bietolini S, Ottolenghi L. Impact of Green Tea (Camellia Sinensis) on periodontitis and caries. Systematic review and meta-analysis. Jpn Dent Sci Rev. 2021;57:1–11.

468. Zhang J, Liu Z, Qiu J, Yang J. Impact of locally delivered diphosphonates on dental implants: A systematic review and meta-analysis. J Stomatol Oral Maxillofac Surg. 2023;124:101521.

469. Monje A, Aranda L, Diaz KT, Alarcón MA, Bagramian RA, Wang HL, et al. Impact of Maintenance Therapy for the Prevention of Peri-implant Diseases: A Systematic Review and Meta-analysis. J Dent Res. 2016;95:372–9.

470. Meger MN, Fatturi AL, Gerber JT, Weiss SG, Rocha JS, Scariot R, et al. Impact of orthognathic surgery on quality of life of patients with dentofacial deformity: a systematic review and meta-analysis. Br J Oral Maxillofac Surg. 2021;59:265–71.

471. Lee CT, Huang HY, Sun TC, Karimbux N. Impact of Patient Compliance on Tooth Loss during Supportive Periodontal Therapy: A Systematic Review and Meta-analysis. J Dent Res. 2015;94:777–86.

472. Abou-Ayash S, Strasding M, Rücker G, Att W. Impact of prosthetic material on mid- and long-term outcome of dental implants supporting single crowns and fixed partial dentures: A systematic review and meta-analysis. Eur J Oral Implantol. 2017;10 Suppl 1:47–65.

473. Lin C-Y, Chen Z, Pan W-L, Wang H-L. Impact of timing on soft tissue augmentation during implant treatment: A systematic review and meta-analysis. Clin Oral Implants Res. 2018;29:508–21.

474. Vidigal MTC, Mesquita CM, de Oliveira MN, de Andrade Vieira W, Blumenberg C, Nascimento GG, et al. Impacts of using orthodontic appliances on the quality of life of children and adolescents: systematic review and meta-analysis. Eur J Orthod. 2022;44:359–68.

475. Roca-Millan E, González-Navarro B, Domínguez-Mínger J, Marí-Roig A, Jané-Salas E, López-López J. Implant insertion torque and marginal bone loss: A systematic review and meta-analysis. Int J Oral Implantol (Berl). 2020;13:345–53.

476. Papaspyridakos P, Chen C-J, Chuang S-K, Weber H-P. Implant loading protocols for edentulous patients with fixed prostheses: a systematic review and meta-analysis. Int J Oral Maxillofac Implants. 2014;29 Suppl:256–70.

477. Pardal-Peláez B, Flores-Fraile J, Pardal-Refoyo J-L, Montero J. Implant loss and crestal bone loss in early loading versus delayed and immediate loading in edentulous mandibles. A systematic review and meta-analysis. J Clin Exp Dent. 2021;13:e397–405.

478. Pardal-Peláez B, Flores-Fraile J, Pardal-Refoyo JL, Montero J. Implant loss and crestal bone loss in immediate versus delayed load in edentulous mandibles: A systematic review and meta-analysis. J Prosthet Dent. 2021;125:437–44.

479. Alqutaibi AY, Elawady DMA. Implant splinting in mandibular overdentures: a systematic review with meta-analysis of randomized clinical trials. Quintessence Int. 2020;51:294–302.

480. Almassri HNS, Ma Y, Dan Z, Ting Z, Cheng Y, Wu X. Implant stability and survival rates of a hydrophilic versus a conventional sandblasted, acid-etched implant surface: Systematic review and meta-analysis. J Am Dent Assoc. 2020;151:444–53.

481. García-Moreno S, González-Serrano J, López-Pintor RM, Pardal-Peláez B, Hernández G, Martínez-González JM. Implant stability using piezoelectric bone surgery compared with conventional drilling: a systematic review and meta-analysis. Int J Oral Maxillofac Surg. 2018;47:1453–64.

482. Srinivasan M, Makarov NA, Herrmann FR, Müller F. Implant survival in 1- versus 2-implant mandibular overdentures: a systematic review and meta-analysis. Clin Oral Implants Res. 2016;27:63–72.

483. Moraschini V, Velloso G, Luz D, Barboza EP. Implant survival rates, marginal bone level changes, and complications in full-mouth rehabilitation with flapless computer-guided surgery: a systematic review and meta-analysis. Int J Oral Maxillofac Surg. 2015;44:892–901.

484. Casaña-Ruiz MD, Català-Pizarro M, Borrás-Aviñó C, Estrela-Sanchís MF, Bellot-Arcís C, Montiel-Company JM. Implants as a treatment alternative in children with multiple agnesia: Systematic review and meta-analysis. J Clin Exp Dent. 2023;15:e324–37.

485. Sourvanos D, Poon J, Lander B, Sarmiento H, Carroll J, Zhu TC, et al. Improving Titanium Implant Stability with Photobiomodulation: A Review and Meta-Analysis of Irradiation Parameters. Photobiomodul Photomed Laser Surg. 2023;41:93–103.

486. Mocharko V, Mascarenhas P, Azul AM, Delgado AHS. In Search of Novel Degradation-Resistant Monomers for Adhesive Dentistry: A Systematic Review and Meta-Analysis. Biomedicines. 2022;10:3104.

487. Ockerman A, Bornstein MM, Leung YY, Li SKY, Politis C, Jacobs R. Incidence of bleeding after minor oral surgery in patients on dual antiplatelet therapy: a systematic review and meta-analysis. Int J Oral Maxillofac Surg. 2020;49:90–8.

488. Khazaei S, Shirani AM, Khazaei M, Najafi F. Incidence of cleft lip and palate in Iran. A meta-analysis. Saudi Med J. 2011;32:390–3.

489. Pesce P, Del Fabbro M, Modenese L, Sandron S, Francetti L, Isola G, et al. Influence of implant diameter on implant survival rate and clinical outcomes in the posterior area: a systematic review and meta-analysis. BMC Oral Health. 2023;23:235.

490. Di Gianfilippo R, Valente NA, Toti P, Wang HL, Barone A. Influence of implant mucosal thickness on early bone loss: a systematic review with meta-analysis. J Periodontal Implant Sci. 2020;50:209–25.

491. Atieh MA, Shah M, Ameen M, Tawse-Smith A, Alsabeeha NHM. Influence of implant restorative emergence angle and contour on peri-implant marginal bone loss: A systematic review and meta-analysis. Clin Implant Dent Relat Res. 2023;25:840–52.

492. Garaicoa-Pazmino C, Lin G-H, Alkandery A, Parra-Carrasquer C, Suárez-López Del Amo F. Influence of implant surface characteristics on the initiation, progression and treatment outcomes of peri-implantitis: A systematic review and meta-analysis based on animal model studies. Int J Oral Implantol (Berl). 2021;14:367–82.

493. Al Amri MD. Influence of interimplant distance on the crestal bone height around dental implants: A systematic review and meta-analysis. J Prosthet Dent. 2016;115:278-282.e1.

494. Javed F, Kellesarian SV, Abduljabbar T, Abduljabbar AT, Akram Z, Vohra F, et al. Influence of involuntary cigarette smoke inhalation on osseointegration: a systematic review and meta-analysis of preclinical studies. Int J Oral Maxillofac Surg. 2018;47:764–72.

495. Chen Z, Zhang Y, Li J, Wang H-L, Yu H. Influence of Laser-Microtextured Surface Collar on Marginal Bone Loss and Peri-Implant Soft Tissue Response: A Systematic Review and Meta-Analysis. J Periodontol. 2017;88:651–62.

496. Darriba I, Seidel A, Moreno F, Botelho J, Machado V, Mendes JJ, et al. Influence of low insertion torque values on survival rate of immediately loaded dental implants: A systematic review and meta-analysis. J Clin Periodontol. 2023;50:158–69.

497. Rahbani Nobar B, Dianat O, Rahbani Nobar B, Kazem M, Hicks ML. Influence of minimally invasive access cavities on load capacity of root-canal-treated teeth: A systematic review and meta-analysis. Aust Endod J. 2023;49:213–36.

498. Lima IFP, de Andrade Vieira W, de Macedo Bernardino Í, Costa PA, Lima APB, Pithon MM, et al. Influence of reminder therapy for controlling bacterial plaque in patients undergoing orthodontic treatment: A systematic review and meta-analysis. Angle Orthod. 2018;88:483–93.

499. Bressan E, Guazzo R, Tomasi C, Peña TG, Galindo-Moreno P, Caponio VCA, et al. Influence of soft tissue thickness on marginal bone level around dental implants: A systematic review with meta-analysis and trial-sequential analysis. Clin Oral Implants Res. 2023;34:405–15.

500. Suárez-López Del Amo F, Lin G-H, Monje A, Galindo-Moreno P, Wang H-L. Influence of Soft Tissue Thickness on Peri-Implant Marginal Bone Loss: A Systematic Review and Meta-Analysis. J Periodontol. 2016;87:690–9.

501. Dioguardi M, Arena C, Sovereto D, Aiuto R, Laino L, Illuzzi G, et al. Influence of sterilization procedures on the physical and mechanical properties of rotating endodontic instruments: a systematic review and network meta-analysis. Front Biosci (Landmark Ed). 2021;26:1697–713.

502. Valles C, Rodríguez-Ciurana X, Clementini M, Baglivo M, Paniagua B, Nart J. Influence of subcrestal implant placement compared with equicrestal position on the peri-implant hard and soft tissues around platform-switched implants: a systematic review and meta-analysis. Clin Oral Investig. 2018;22:555–70.

503. Díaz-Sánchez M, Soto-Peñaloza D, Peñarrocha-Oltra D, Peñarrocha-Diago M. Influence of supracrestal tissue attachment thickness on radiographic bone level around dental implants: A systematic review and meta-analysis. J Periodontal Res. 2019;54:573–88.

504. Muñoz M, Vilarrasa J, Ruíz-Magaz V, Albertini M, Nart J. Influence of the abutment height on marginal bone level changes around two-piece dental implants: Meta-analysis and trial sequential analysis of randomized clinical trials. Clin Oral Implants Res. 2023;34:81–94.

505. Rossoni NB, Cavalheiro CP, Casagrande L, Lenzi TL. Influence of the chemomechanical and mechanical carious tissue removal on the risk of restorative failure: a systematic review and meta-analysis. Clin Oral Investig. 2022;26:6457–67.

506. Armond ACV, Martins CC, Glória JCR, Galvão EL, Dos Santos CRR, Falci SGM. Influence of third molars in mandibular fractures. Part 1: mandibular angle-a meta-analysis. Int J Oral Maxillofac Surg. 2017;46:716–29.

507. Armond ACV, Martins CC, Glória JCR, Galvão EL, Dos Santos CRR, Falci SGM. Influence of third molars in mandibular fractures. Part 2: mandibular condyle-a meta-analysis. Int J Oral Maxillofac Surg. 2017;46:730–9.

508. Liu J, Hua F, Zhang H, Hu J. Influence of using collagen on the soft and hard tissue outcomes of immediate dental implant placement: A systematic review and meta-analysis. J Stomatol Oral Maxillofac Surg. 2023;124:101385.

509. Camps-Font O, Burgueño-Barris G, Figueiredo R, Jung RE, Gay-Escoda C, Valmaseda-Castellón E. Interventions for Dental Implant Placement in Atrophic Edentulous Mandibles: Vertical Bone Augmentation and Alternative Treatments. A Meta-Analysis of Randomized Clinical Trials. J Periodontol. 2016;87:1444–57.

510. Koletsi D, Belibasakis GN, Eliades T. Interventions to Reduce Aerosolized Microbes in Dental Practice: A Systematic Review with Network Meta-analysis of Randomized Controlled Trials. J Dent Res. 2020;99:1228–38.

511. Wambier LM, de Geus JL, Chibinski ACR, Wambier DS, Rego RO, Loguercio AD, et al. Intra-pocket anaesthesia and pain during probing, scaling and root planing: a systematic review and meta-analysis. J Clin Periodontol. 2016;43:754–66.

512. Pozos-Guillen A, Garcia-Flores A, Esparza-Villalpando V, Garrocho-Rangel A. Intracanal irrigants for pulpectomy in primary teeth: a systematic review and meta-analysis. Int J Paediatr Dent. 2016;26:412–25.

513. Fernandes IA, de Souza GM, Pinheiro MLP, Falci SGM. Intramuscular injection of dexamethasone for the control of pain, swelling, and trismus after third molar surgery: a systematic review and meta-analysis. Int J Oral Maxillofac Surg. 2019;48:659–68.

514. Canellas JVDS, Fraga SRG, Santoro MF, Netto J de NS, Tinoco EMB. Intrasocket interventions to prevent alveolar osteitis after mandibular third molar surgery: A systematic review and network meta-analysis. J Craniomaxillofac Surg. 2020;48:902–13.

515. Pereira LJ, Andrade EF, Barroso LC, Lima RR de, Macari S, Paiva SM, et al. Irisin effects on bone: systematic review with meta-analysis of preclinical studies and prospects for oral health. Braz Oral Res. 2022;36:e055.

516. Smith Nobrega A, Santiago JF, de Faria Almeida DA, Dos Santos DM, Pellizzer EP, Goiato MC. Irradiated patients and survival rate of dental implants: A systematic review and meta-analysis. J Prosthet Dent. 2016;116:858–66.

517. Calcia TBB, Oballe HJR, de Oliveira Silva AM, Friedrich SA, Muniz FWMG. Is alteration in single drug anticoagulant/antiplatelet regimen necessary in patients who need minor oral surgery? A systematic review with meta-analysis. Clin Oral Investig. 2021;25:3369–81.

518. Fraga RS, Antunes LAA, Fontes KBF da C, Küchler EC, Iorio NLPP, Antunes LS. Is Antimicrobial Photodynamic Therapy Effective for Microbial Load Reduction in Peri-implantitis Treatment? A Systematic Review and Meta-Analysis. Photochem Photobiol. 2018;94:752–9.

519. Apparaju V, Vaddamanu SK, Vyas R, Vishwanath S, Gurumurthy V, Kanji MA. Is balloon-assisted maxillary sinus floor augmentation before dental implant safe and promising? A systematic review and meta-analysis. Niger J Clin Pract. 2020;23:275–83.

520. Arteagoitia I, Sánchez FR, Figueras A, Arroyo-Lamas N. Is clindamycin effective in preventing infectious complications after oral surgery? Systematic review and meta-analysis of randomized controlled trials. Clin Oral Investig. 2022;26:4467–78.

521. Vieira WA, Paranhos LR, Cericato GO, Franco A, Ribeiro M a. G. Is mepivacaine as effective as lidocaine during inferior alveolar nerve blocks in patients with symptomatic irreversible pulpitis? A systematic review and meta-analysis. Int Endod J. 2018;51:1104–17.

522. Ammari MM, Soviero VM, da Silva Fidalgo TK, Lenzi M, Ferreira DMTP, Mattos CT, et al. Is non-cavitated proximal lesion sealing an effective method for caries control in primary and permanent teeth? A systematic review and meta-analysis. J Dent. 2014;42:1217–27.

523. Xiong X, Xu T, Wang X, Qin W, Yu T, Luo G. Is oral lichen planus a risk factor for peri-implant diseases? A systematic review and meta-analysis. BMC Oral Health. 2020;20:150.

524. Sunnak R, Johal A, Fleming PS. Is orthodontics prior to 11 years of age evidence-based? A systematic review and meta-analysis. J Dent. 2015;43:477–86.

525. Mheissen S, Khan H, Samawi S. Is Piezocision effective in accelerating orthodontic tooth movement: A systematic review and meta-analysis. PLoS One. 2020;15:e0231492.

526. Swaikat M, Faus-Matoses I, Zubizarreta-Macho Á, Ashkar I, Faus-Matoses V, Bellot-Arcís C, et al. Is Revascularization the Treatment of Choice for Traumatized Necrotic Immature Teeth? A Systematic Review and Meta-Analysis. J Clin Med. 2023;12:2656.

527. Traboulsi-Garet B, Jorba-García A, Camps-Font O, Alves FA, Figueiredo R, Valmaseda-Castellón E. Is serum C-terminal telopeptide cross-link of type 1 collagen a reliable parameter for predicting the risk of medication-related osteonecrosis of the jaws? A systematic review and meta-analysis of diagnostic test accuracy. Clin Oral Investig. 2022;26:2371–82.

528. Rodrigues VVM, Faé DS, Rosa CDDRD, Bento VAA, Lacerda MFLS, Pellizzer EP, et al. Is the clinical performance of internal conical connection better than internal non-conical connection for implant-supported restorations? A systematic review with meta-analysis of randomized controlled trials. J Prosthodont. 2023;32:382–91.

529. Alves Dos Santos GN, Faria-E-Silva AL, Ribeiro VL, Pelozo LL, Candemil AP, Oliveira ML, et al. Is the quality of root canal filling obtained by cone-beam computed tomography associated with periapical lesions? A systematic review and meta-analysis. Clin Oral Investig. 2022;26:5105–16.

530. Souto-Souza D, Mourão PS, Barroso HH, Douglas-de-Oliveira DW, Ramos-Jorge ML, Falci SGM, et al. Is there an association between attention deficit hyperactivity disorder in children and adolescents and the occurrence of bruxism? A systematic review and meta-analysis. Sleep Med Rev. 2020;53:101330.

531. Dos Anjos AMC, Moura de Lima M de D, Muniz FWMG, Lima CCB, Moura L de FA de D, Rösing CK, et al. Is there an association between dental caries and genetics? Systematic review and meta-analysis of studies with twins. J Dent. 2023;135:104586.

532. Monteiro JLGC, Pellizzer EP, Araújo Lemos CA, de Moraes SLD, do Egito Vasconcelos BC. Is there an association between overweight/obesity and dental implant complications? A systematic review and meta-analysis. Int J Oral Maxillofac Surg. 2019;48:1241–9.

533. Silva CCG, Dos Santos MS, Monteiro JLGC, de Aguiar Soares Carneiro SC, do Egito Vasconcelos BC. Is there an association between the use of antidepressants and complications involving dental implants? A systematic review and meta-analysis. Int J Oral Maxillofac Surg. 2021;50:96–103.

534. Moraschini V, Kischinhevsky ICC, Sartoretto SC, Shibli JA, Dias AT, Sacco R, et al. Is there any biomaterial substitute for peri-implant soft tissue phenotype modification? A network meta-analysis of the appraisal literature. Int J Oral Maxillofac Surg. 2022;51:526–34.

535. Al-Akhali MS, Al-Moraissi EA. Khat chewing habit produces a significant adverse effect on periodontal, oral health: A systematic review and meta-analysis. J Periodontal Res. 2017;52:937–45.

536. de Moura RC, Santos PS, Matias PMDS, Vitali FC, Hilgert LA, Cardoso M, et al. Knowledge, attitudes, and practice of dentists on Minimal Intervention Dentistry: A systematic review and meta-analysis. J Dent. 2023;132:104484.

537. Protásio ACR, Galvão EL, Falci SGM. Laser Techniques or Scalpel Incision for Labial Frenectomy: A Meta-analysis. J Maxillofac Oral Surg. 2019;18:490–9.

538. Deng Y, Zhu X, Zheng D, Yan P, Jiang H. Laser use in direct pulp capping: A meta-analysis. J Am Dent Assoc. 2016;147:935–42.

539. Gorgis R, Qazo L, Bruun NH, Starch-Jensen T. Lateral Alveolar Ridge Augmentation with an Autogenous Bone Block Graft Alone with or without Barrier Membrane Coverage: a Systematic Review and Meta-Analysis. J Oral Maxillofac Res. 2021;12:e1.

540. Dongo V, von Krockow N, Martins-Filho PRS, Weigl P. Lateral sinus floor elevation without grafting materials. Individual- and aggregate-data meta-analysis. J Craniomaxillofac Surg. 2018;46:1616–24.

541. Naseri R, Yaghini J, Feizi A. Levels of smoking and dental implants failure: A systematic review and meta-analysis. J Clin Periodontol. 2020;47:518–28.

542. Afshari Z, Yaghini J, Naseri R. LEVELS OF SMOKING AND PERI‑IMPLANT MARGINAL BONE LOSS: A SYSTEMATIC REVIEW AND META-ANALYSIS. J Evid Based Dent Pract. 2022;22:101721.

543. Bakdach WMM, Hadad R. Linear and angular transfer accuracy of labial brackets using three dimensional-printed indirect bonding trays: A systematic review and meta-analysis. Int Orthod. 2022;20:100612.

544. Raghoebar GM, Korfage A, Meijer HJA, Gareb B, Vissink A, Delli K. Linear and profilometric changes of the mucosa following soft tissue augmentation in the zone of aesthetic priority: A systematic review and meta-analysis. Clin Oral Implants Res. 2021;32 Suppl 21:138–56.

545. Papageorgiou SN, Gölz L, Jäger A, Eliades T, Bourauel C. Lingual vs. labial fixed orthodontic appliances: systematic review and meta-analysis of treatment effects. Eur J Oral Sci. 2016;124:105–18.

546. Yang F, Gao Y, Zhang L, Zheng B, Wang L, Sun H, et al. Local anaesthesia for surgical extraction of mandibular third molars: a systematic review and network meta-analysis. Clin Oral Investig. 2020;24:3781–800.

547. Guimaraes CC, Lopes LC, Bergamaschi C de C, Ramacciato JC, Silva MT, Araújo J de O, et al. Local anaesthetics combined with vasoconstrictors in patients with cardiovascular disease undergoing dental procedures: systematic review and meta-analysis. BMJ Open. 2021;11:e044357.

548. Guimaraes CC, Lopes Motta RH, Bergamaschi C de C, Araújo J de O, Andrade NK de, Figueiró MF, et al. Local anaesthetics combined with vasoconstrictors in patients with cardiovascular disease undergoing dental procedures: systematic review and meta-analysis protocol. BMJ Open. 2017;7:e014611.

549. Dioguardi M, Spirito F, Alovisi M, Aiuto R, Garcovich D, Crincoli V, et al. Location and Gender Differences in Osteonecrosis of the Jaws in Patients Treated with Antiresorptive and Antineoplastic Drugs Undergoing Dentoalveolar Surgical, Systematic Review with Meta-Analysis and Trial Sequential Analysis. J Clin Med. 2023;12:3299.

550. Howe M-S, Keys W, Richards D. Long-term (10-year) dental implant survival: A systematic review and sensitivity meta-analysis. J Dent. 2019;84:9–21.

551. Salvi GE, Monje A, Tomasi C. Long-term biological complications of dental implants placed either in pristine or in augmented sites: A systematic review and meta-analysis. Clin Oral Implants Res. 2018;29 Suppl 16:294–310.

552. Cortés-Bretón Brinkmann J, García-Gil I, Pedregal P, Peláez J, Prados-Frutos JC, Suárez MJ. Long-Term Clinical Behavior and Complications of Intentionally Tilted Dental Implants Compared with Straight Implants Supporting Fixed Restorations: A Systematic Review and Meta-Analysis. Biology (Basel). 2021;10:509.

553. Zhao G, Zhou Y, Shi S, Liu X, Zhang S, Song Y. Long-term clinical outcomes of immediate loading versus non-immediate loading in single-implant restorations: a systematic review and meta-analysis. Int J Oral Maxillofac Surg. 2022;51:1345–54.

554. Heydari M, Ataei A, Riahi SM. Long-Term Effect of Keratinized Tissue Width on Peri-implant Health Status Indices: An Updated Systematic Review and Meta-analysis. Int J Oral Maxillofac Implants. 2021;36:1065–75.

555. Ndjidda Bakari W, Diallo AM, Danwang C, Nzalie RNT, Benoist HM. Long-term effect of non-surgical periodontal treatment on glycaemic control in patients with diabetes with periodontitis: a systematic review and meta-analysis protocol. BMJ Open. 2021;11:e043250.

556. Doornewaard R, Christiaens V, De Bruyn H, Jacobsson M, Cosyn J, Vervaeke S, et al. Long-Term Effect of Surface Roughness and Patients’ Factors on Crestal Bone Loss at Dental Implants. A Systematic Review and Meta-Analysis. Clin Implant Dent Relat Res. 2017;19:372–99.

557. Raghoebar GM, Onclin P, Boven GC, Vissink A, Meijer HJA. Long-term effectiveness of maxillary sinus floor augmentation: A systematic review and meta-analysis. J Clin Periodontol. 2019;46 Suppl 21:307–18.

558. Chambrone L, Tatakis DN. Long-Term Outcomes of Untreated Buccal Gingival Recessions: A Systematic Review and Meta-Analysis. J Periodontol. 2016;87:796–808.

559. Gonçalves TMSV, Bortolini S, Martinolli M, Alfenas BFM, Peruzzo DC, Natali A, et al. Long-term Short Implants Performance: Systematic Review and Meta-Analysis of the Essential Assessment Parameters. Braz Dent J. 2015;26:325–36.

560. Cao Y, Yu C, Wu Y, Li L, Li C. Long-Term Survival and Peri-Implant Health of Titanium Implants with Zirconia Abutments: A Systematic Review and Meta-Analysis. J Prosthodont. 2019;28:883–92.

561. Duttenhoefer F, Souren C, Menne D, Emmerich D, Schön R, Sauerbier S. Long-term survival of dental implants placed in the grafted maxillary sinus: systematic review and meta-analysis of treatment modalities. PLoS One. 2013;8:e75357.

562. Taylor M, Masood M, Mnatzaganian G. Longevity of complete dentures: A systematic review and meta-analysis. J Prosthet Dent. 2021;125:611–9.

563. da Veiga AMA, Cunha AC, Ferreira DMTP, da Silva Fidalgo TK, Chianca TK, Reis KR, et al. Longevity of direct and indirect resin composite restorations in permanent posterior teeth: A systematic review and meta-analysis. J Dent. 2016;54:1–12.

564. Opdam NJM, van de Sande FH, Bronkhorst E, Cenci MS, Bottenberg P, Pallesen U, et al. Longevity of posterior composite restorations: a systematic review and meta-analysis. J Dent Res. 2014;93:943–9.

565. Akram Z, Vohra F, Javed F. Low-level laser therapy as an adjunct to connective tissue graft procedure in the treatment of gingival recession defects: A systematic review and meta-analysis. J Esthet Restor Dent. 2018;30:299–306.

566. Doğramacı EJ, Rossi-Fedele G, Dreyer CW. Malocclusions in young children: Does breast-feeding really reduce the risk? A systematic review and meta-analysis. J Am Dent Assoc. 2017;148:566-574.e6.

567. Dioguardi M, Sovereto D, Illuzzi G, Laneve E, Raddato B, Arena C, et al. Management of Instrument Sterilization Workflow in Endodontics: A Systematic Review and Meta-Analysis. Int J Dent. 2020;2020:5824369.

568. Liu C, Xiong Y-T, Zhu T, Liu W, Tang W, Zeng W. Management of Tooth Extraction in Patients Taking Antiresorptive Drugs: An Evidence Mapping Review and Meta-Analysis. J Clin Med. 2022;12:239.

569. Troiano G, Laino L, Dioguardi M, Giannatempo G, Lo Muzio L, Lo Russo L. Mandibular Class II Furcation Defect Treatment: Effects of the Addition of Platelet Concentrates to Open Flap: A Systematic Review and Meta-Analysis of Randomized Clinical Trials. J Periodontol. 2016;87:1030–8.

570. Fuda S, Martins BGDS, Castro FC de, Heboyan A, Gehrke SA, Fernandes JCH, et al. Marginal Bone Level and Clinical Parameter Analysis Comparing External Hexagon and Morse Taper Implants: A Systematic Review and Meta-Analysis. Diagnostics (Basel). 2023;13:1587.

571. Laurell L, Lundgren D. Marginal bone level changes at dental implants after 5 years in function: a meta-analysis. Clin Implant Dent Relat Res. 2011;13:19–28.

572. Taheri M, Akbari S, Shamshiri AR, Shayesteh YS. Marginal bone loss around bone-level and tissue-level implants: A systematic review and meta-analysis. Ann Anat. 2020;231:151525.

573. Carbajal Mejía JB, Wakabayashi K, Nakano T, Yatani H. Marginal Bone Loss Around Dental Implants Inserted with Static Computer Assistance in Healed Sites: A Systematic Review and Meta-analysis. Int J Oral Maxillofac Implants. 2016;31:761–75.

574. Niu W, Wang P, Zhu S, Liu Z, Ji P. Marginal bone loss around dental implants with and without microthreads in the neck: A systematic review and meta-analysis. J Prosthet Dent. 2017;117:34–40.

575. Starch-Jensen T, Deluiz D, Bruun NH, Tinoco EMB. Maxillary Sinus Floor Augmentation with Autogenous Bone Graft Alone Compared with Alternate Grafting Materials: a Systematic Review and Meta-Analysis Focusing on Histomorphometric Outcome. J Oral Maxillofac Res. 2020;11:e2.

576. Starch-Jensen T, Deluiz D, Vitenson J, Bruun NH, Tinoco EMB. Maxillary Sinus Floor Augmentation with Autogenous Bone Graft Compared with a Composite Grafting Material or Bone Substitute Alone: a Systematic Review and Meta-Analysis Assessing Volumetric Stability of the Grafting Material. J Oral Maxillofac Res. 2021;12:e1.

577. Starch-Jensen T, Deluiz D, Duch K, Tinoco EMB. Maxillary Sinus Floor Augmentation With or Without Barrier Membrane Coverage of the Lateral Window: a Systematic Review and Meta-Analysis. J Oral Maxillofac Res. 2019;10:e1.

578. Valenti C, Isabella Federici M, Masciotti F, Marinucci L, Xhimitiku I, Cianetti S, et al. Mechanical properties of 3D-printed prosthetic materials compared with milled and conventional processing: A systematic review and meta-analysis of in vitro studies. J Prosthet Dent. 2022;S0022-3913(22)00415-2.

579. Lourinho C, Salgado H, Correia A, Fonseca P. Mechanical Properties of Polymethyl Methacrylate as Denture Base Material: Heat-Polymerized vs. 3D-Printed-Systematic Review and Meta-Analysis of In Vitro Studies. Biomedicines. 2022;10:2565.

580. Li G-F, Yang Z-J, Wang T-C, Zhang C-X, Zhang Ji-Y, Chen J-D, et al. Meta-analysis dataset comparing orthodontic mini-implants and conventional anchorage reinforcement for maximum orthodontic anchorage. Data Brief. 2020;32:106010.

581. Rakhshan V, Rakhshan H. Meta-analysis of congenitally missing teeth in the permanent dentition: Prevalence, variations across ethnicities, regions and time. Int Orthod. 2015;13:261–73.

582. Li H, Liang Y, Zheng Q. Meta-Analysis of Correlations Between Marginal Bone Resorption and High Insertion Torque of Dental Implants. Int J Oral Maxillofac Implants. 2015;30:767–72.

583. Muddugangadhar BC, Amarnath GS, Sonika R, Chheda PS, Garg A. Meta-analysis of Failure and Survival Rate of Implant-supported Single Crowns, Fixed Partial Denture, and Implant Tooth-supported Prostheses. J Int Oral Health. 2015;7:11–7.

584. Ravidà A, Wang I-C, Barootchi S, Askar H, Tavelli L, Gargallo-Albiol J, et al. Meta-analysis of randomized clinical trials comparing clinical and patient-reported outcomes between extra-short (≤6 mm) and longer (≥10 mm) implants. J Clin Periodontol. 2019;46:118–42.

585. Báez V, Corcos L, Morgillo F, Imperatrice L, Gualtieri AF. Meta-analysis of regenerative endodontics outcomes with antibiotics pastes and calcium hydroxide. The apex of the iceberg. J Oral Biol Craniofac Res. 2022;12:90–8.

586. Yang S, Gu B, Zhao L, Shi Q, Xu J, Wen N. Meta-analysis of the association between serum and gingival crevicular fluid matrix metalloproteinase-9 and periodontitis. J Am Dent Assoc. 2019;150:34–41.

587. Biddle C. Meta-analysis of the effectiveness of nonsteroidal anti-inflammatory drugs in a standardized pain model. AANA J. 2002;70:111–4.

588. Corica A, Caprioglio A. Meta-analysis of the prevalence of tooth wear in primary dentition. Eur J Paediatr Dent. 2014;15:385–8.

589. Liang A, Huang L, Li B, Huang Y, Zhou X, Zhang X, et al. Micro-CT Evaluation of Different Root Canal Irrigation Protocols on the Removal of Accumulated Hard Tissue Debris: A Systematic Review and Meta-Analysis. J Clin Med. 2022;11:6053.

590. Zotti F, Falavigna E, Capocasale G, De Santis D, Albanese M. Microleakage of Direct Restorations-Comparisonbetween Bulk-Fill and Traditional Composite Resins:Systematic Review and Meta-Analysis. Eur J Dent. 2021;15:755–67.

591. Vi S, Pham D, Du YYM, Arora H, Tadakamadla SK. Mini-Implant-Retained Overdentures for the Rehabilitation of Completely Edentulous Maxillae: A Systematic Review and Meta-Analysis. Int J Environ Res Public Health. 2021;18:4377.

592. Marcello-Machado RM, Faot F, Schuster AJ, Nascimento GG, Del Bel Cury AA. Mini-implants and narrow diameter implants as mandibular overdenture retainers: A systematic review and meta-analysis of clinical and radiographic outcomes. J Oral Rehabil. 2018;45:161–83.

593. Thoma DS, Strauss FJ, Mancini L, Gasser TJW, Jung RE. Minimal invasiveness in soft tissue augmentation at dental implants: A systematic review and meta-analysis of patient-reported outcome measures. Periodontol 2000. 2023;91:182–98.

594. Liu S, Hu B, Zhang Y, Li W, Song J. Minimally Invasive Surgery Combined with Regenerative Biomaterials in Treating Intra-Bony Defects: A Meta-Analysis. PLoS One. 2016;11:e0147001.

595. Alharbi F, Almuzian M, Bearn D. Miniscrews failure rate in orthodontics: systematic review and meta-analysis. Eur J Orthod. 2018;40:519–30.

596. Paul S, Petsch M, Held U. Modeling of Crestal Bone After Submerged vs Transmucosal Implant Placement: A Systematic Review with Meta-Analysis. Int J Oral Maxillofac Implants. 2017;32:1039–50.

597. Carter K, Worthington S. Morphologic and Demographic Predictors of Third Molar Agenesis: A Systematic Review and Meta-analysis. J Dent Res. 2015;94:886–94.

598. Choubey S, Patil A, Talekar AL, Kalra D. Nanosilver fluoride as a caries arresting agent in children: A systematic review and meta- analysis. J Indian Soc Pedod Prev Dent. 2022;40:230–8.

599. González-Valls G, Roca-Millan E, Céspedes-Sánchez JM, González-Navarro B, Torrejon-Moya A, López-López J. Narrow Diameter Dental Implants as an Alternative Treatment for Atrophic Alveolar Ridges. Systematic Review and Meta-Analysis. Materials (Basel). 2021;14:3234.

600. Cruz RS, Lemos C a. A, de Batista VES, Yogui FC, Oliveira HFF, Verri FR. Narrow-diameter implants versus regular-diameter implants for rehabilitation of the anterior region: a systematic review and meta-analysis. Int J Oral Maxillofac Surg. 2021;50:674–82.

601. Schiegnitz E, Al-Nawas B. Narrow-diameter implants: A systematic review and meta-analysis. Clin Oral Implants Res. 2018;29 Suppl 16:21–40.

602. Park J-H, Shin S-W, Lee J-Y. Narrow-diameter versus regular-diameter dental implants for mandibular overdentures: A systematic review and meta-analysis. J Prosthodont. 2023;32:669–78.

603. Nardello LCL, Pinheiro ET, Gavini G, Prado LC, Romero RX, Gomes BPFA, et al. Nature and Prevalence of Bacterial Taxa Persisting after Root Canal Chemomechanical Preparation in Permanent Teeth: A Systematic Review and Meta-analysis. J Endod. 2022;48:572–96.

604. Faggion CM, Chambrone L, Listl S, Tu Y-K. Network meta-analysis for evaluating interventions in implant dentistry: the case of peri-implantitis treatment. Clin Implant Dent Relat Res. 2013;15:576–88.

605. Gölz L, Papageorgiou SN, Jäger A. Nickel hypersensitivity and orthodontic treatment: a systematic review and meta-analysis. Contact Dermatitis. 2015;73:1–14.

606. Watanabe T, Sieg M, Lunde SJ, Persson M, Taneja P, Baad-Hansen L, et al. Nocebo response in dentistry: A systematic review and meta-analysis of adverse events in analgesic trials of third molar removal. J Oral Rehabil. 2023;50:332–42.

607. Rojo-Sanchis C, Montiel-Company JM, Tarazona-Álvarez B, Haas-Junior OL, Peiró-Guijarro MA, Paredes-Gallardo V, et al. Non-Surgical Management of the Gingival Smile with Botulinum Toxin A-A Systematic Review and Meta-Analysis. J Clin Med. 2023;12:1433.

608. Musa AAR, Sethi S, Poirier BF, Oliver KJ, Jensen ED. Non-traumatic emergency department dental visits among patients 0-25 years of age: A systematic review and meta-analysis. Int J Paediatr Dent. 2023;33:457–67.

609. Urquhart O, Tampi MP, Pilcher L, Slayton RL, Araujo MWB, Fontana M, et al. Nonrestorative Treatments for Caries: Systematic Review and Network Meta-analysis. J Dent Res. 2019;98:14–26.

610. Smith EA, Marshall JG, Selph SS, Barker DR, Sedgley CM. Nonsteroidal Anti-inflammatory Drugs for Managing Postoperative Endodontic Pain in Patients Who Present with Preoperative Pain: A Systematic Review and Meta-analysis. J Endod. 2017;43:7–15.

611. Barootchi S, Ravidà A, Tavelli L, Wang H-L. Nonsurgical treatment for peri-implant mucositis: A systematic review and meta-analysis. Int J Oral Implantol (Berl). 2020;13:123–39.

612. Hardan L, Bourgi R, Cuevas-Suárez CE, Lukomska-Szymanska M, Monjarás-Ávila AJ, Zarow M, et al. Novel Trends in Dental Color Match Using Different Shade Selection Methods: A Systematic Review and Meta-Analysis. Materials (Basel). 2022;15:468.

613. Daudt Polido W, Aghaloo T, Emmett TW, Taylor TD, Morton D. Number of implants placed for complete-arch fixed prostheses: A systematic review and meta-analysis. Clin Oral Implants Res. 2018;29 Suppl 16:154–83.

614. Granja GL, Lacerda-Santos JT, Firmino RT, Jiao R, Martins CC, Granville-Garcia AF, et al. Occurrence of bruxism in individuals with autism spectrum disorder: A systematic review and meta-analysis. Spec Care Dentist. 2022;42:476–85.

615. Vatėnas I, Linkevičius T. One abutment one time vs. repeatable abutment disconnections in implants, restored with cemented / screw retained fixed partial dentures: Marginal bone level changes. A systematic review and meta-analysis. Stomatologija. 2021;23:35–40.

616. Kang Y, Zheng X, Zhang D, Li S, Xu S, Chen L, et al. One-Abutment at One-Time in Posterior Edentulism: A Systematic Review and Meta-Analysis. J Craniofac Surg. 2023;34:e720–4.

617. Sjögren P, Wårdh I, Zimmerman M, Almståhl A, Wikström M. Oral Care and Mortality in Older Adults with Pneumonia in Hospitals or Nursing Homes: Systematic Review and Meta-Analysis. J Am Geriatr Soc. 2016;64:2109–15.

618. Qi X, Northridge ME, Hu M, Wu B. Oral health conditions and COVID-19: A systematic review and meta-analysis of the current evidence. Aging Health Res. 2022;2:100064.

619. Lai YYL, Zafar S, Leonard HM, Walsh LJ, Downs JA. Oral health education and promotion in special needs children: Systematic review and meta-analysis. Oral Dis. 2022;28:66–75.

620. Brouns VEHW, de Waal A-LML, Bronkhorst EM, Kuijpers-Jagtman AM, Ongkosuwito EM. Oral health-related quality of life before, during, and after orthodontic-orthognathic treatment: a systematic review and meta-analysis. Clin Oral Investig. 2022;26:2223–35.

621. Sivaramakrishnan G, Sridharan K. Oral Ketorolac with Inferior Alveolar Nerve Block for Irreversible Pulpitis: A Systematic Review and Meta-analysis. Open Dent J. 2018;12:340–6.

622. Aragoneses J, Suárez A, Algar J, Rodríguez C, López-Valverde N, Aragoneses JM. Oral Manifestations of COVID-19: Updated Systematic Review With Meta-Analysis. Front Med (Lausanne). 2021;8:726753.

623. Schiegnitz E, Al-Nawas B, Kämmerer PW, Grötz KA. Oral rehabilitation with dental implants in irradiated patients: a meta-analysis on implant survival. Clin Oral Investig. 2014;18:687–98.

624. Ata-Ali J, Peñarrocha-Oltra D, Candel-Marti E, Peñarrocha-Diago M. Oral rehabilitation with tilted dental implants: a metaanalysis. Med Oral Patol Oral Cir Bucal. 2012;17:e582-587.

625. Chen P, Chen Z, Teoh Y-Y, Peters OA, Peters CI. Orifice barriers to prevent coronal microleakage after root canal treatment: systematic review and meta-analysis. Aust Dent J. 2023;68:78–91.

626. Dos Santos A-L-C, Wambier L-M, Wambier D-S, Moreira K-M-S, Imparato J-C-P, Chibinski A-C-R. Orthodontic bracket bonding techniques and adhesion failures: A systematic review and meta-analysis. J Clin Exp Dent. 2022;14:e746–55.

627. Kim M-R, Graber TM, Viana MA. Orthodontics and temporomandibular disorder: a meta-analysis. Am J Orthod Dentofacial Orthop. 2002;121:438–46.

628. Huynh NT, Desplats E, Almeida FR. Orthodontics treatments for managing obstructive sleep apnea syndrome in children: A systematic review and meta-analysis. Sleep Med Rev. 2016;25:84–94.

629. Bidjan D, Sallmann R, Eliades T, Papageorgiou SN. Orthopedic Treatment for Class II Malocclusion with Functional Appliances and Its Effect on Upper Airways: A Systematic Review with Meta-Analysis. J Clin Med. 2020;9:3806.

630. Pieralli S, Kohal R-J, Lopez Hernandez E, Doerken S, Spies BC. Osseointegration of zirconia dental implants in animal investigations: A systematic review and meta-analysis. Dent Mater. 2018;34:171–82.

631. Olivieri JG, Elmsmari F, Miró Q, Ruiz X-F, Krell KV, García-Font M, et al. Outcome and Survival of Endodontically Treated Cracked Posterior Permanent Teeth: A Systematic Review and Meta-analysis. J Endod. 2020;46:455–63.

632. Bassir SH, El Kholy K, Chen C-Y, Lee KH, Intini G. Outcome of early dental implant placement versus other dental implant placement protocols: A systematic review and meta-analysis. J Periodontol. 2019;90:493–506.

633. Setzer FC, Shah SB, Kohli MR, Karabucak B, Kim S. Outcome of endodontic surgery: a meta-analysis of the literature--part 1: Comparison of traditional root-end surgery and endodontic microsurgery. J Endod. 2010;36:1757–65.

634. Setzer FC, Kohli MR, Shah SB, Karabucak B, Kim S. Outcome of endodontic surgery: a meta-analysis of the literature--Part 2: Comparison of endodontic microsurgical techniques with and without the use of higher magnification. J Endod. 2012;38:1–10.

635. Kohli MR, Berenji H, Setzer FC, Lee S-M, Karabucak B. Outcome of Endodontic Surgery: A Meta-analysis of the Literature-Part 3: Comparison of Endodontic Microsurgical Techniques with 2 Different Root-end Filling Materials. J Endod. 2018;44:923–31.

636. Elmsmari F, Ruiz X-F, Miró Q, Feijoo-Pato N, Durán-Sindreu F, Olivieri JG. Outcome of Partial Pulpotomy in Cariously Exposed Posterior Permanent Teeth: A Systematic Review and Meta-analysis. J Endod. 2019;45:1296-1306.e3.

637. Almeida DO, Chaves SC, Souza RA, Soares FF. Outcome of Single- vs Multiple-visit Endodontic Therapy of Nonvital Teeth: A Meta-analysis. J Contemp Dent Pract. 2017;18:330–6.

638. Atieh MA, AlAli F, Alsabeeha NHM. Outcome of supportive peri-implant therapy on the rates of peri-implant diseases and marginal bone loss: a systematic review and meta-analysis. Quintessence Int. 2021;52:122–31.

639. Papageorgiou SN, Höchli D, Eliades T. Outcomes of comprehensive fixed appliance orthodontic treatment: A systematic review with meta-analysis and methodological overview. Korean J Orthod. 2017;47:401–13.

640. Leong DJX, de Souza NN, Sultana R, Yap AU. Outcomes of endodontically treated cracked teeth: a systematic review and meta-analysis. Clin Oral Investig. 2020;24:465–73.

641. Theodoridis C, Grigoriadis A, Menexes G, Vouros I. Outcomes of implant therapy in patients with a history of aggressive periodontitis. A systematic review and meta-analysis. Clin Oral Investig. 2017;21:485–503.

642. Chua DR, Tan BL, Nazzal H, Srinivasan N, Duggal MS, Tong HJ. Outcomes of preformed metal crowns placed with the conventional and Hall techniques: A systematic review and meta-analysis. Int J Paediatr Dent. 2023;33:141–57.

643. Deepthi R, Bilichodmath S. Ozone Therapy in Periodontics: A Meta-analysis. Contemp Clin Dent. 2020;11:108–15.

644. Pozos-Guillén A, Loredo-Cruz E, Esparza-Villalpando V, Martínez-Rider R, Noyola-Frías M, Garrocho-Rangel A. Pain and Anxiety Levels Using Conventional versus Computer-Controlled Local Anesthetic Systems in Pediatric Patients: A Meta-Analysis. J Clin Pediatr Dent. 2020;44:371–99.

645. Salinero L, Boczar D, Barrow B, Berman ZP, Diep GK, Trilles J, et al. Patient-centred outcomes and dental implant placement in computer-aided free flap mandibular reconstruction: a systematic review and meta-analysis. Br J Oral Maxillofac Surg. 2022;60:1283–91.

646. Ribeiro AKC, Costa RTF, Vasconcelos BC do E, de Moraes SLD, Carreiro A da FP, Pellizzer EP. Patient-reported outcome measures and prosthetic events in implant-supported mandibular overdenture patients after immediate versus delayed loading: A systematic review and meta-analysis. J Prosthet Dent. 2022;S0022-3913(22)00190-1.

647. Ladewig NM, Tedesco TK, Gimenez T, Braga MM, Raggio DP. Patient-reported outcomes associated with different restorative techniques in pediatric dentistry: A systematic review and MTC meta-analysis. PLoS One. 2018;13:e0208437.

648. Domokos Z, Uhrin E, Szabó B, Czumbel ML, Dembrovszky F, Kerémi B, et al. Patients with inflammatory bowel disease have a higher chance of developing periodontitis: A systematic review and meta-analysis. Front Med (Lausanne). 2022;9:1020126.

649. Stanková M, Buček A, Dostálová T, Ginzelová K, Pacáková Z, Seydlová M. Patients with special needs within treatment under general anesthesia - meta-analysis. Prague Med Rep. 2011;112:216–25.

650. Roehling S, Schlegel KA, Woelfler H, Gahlert M. Performance and outcome of zirconia dental implants in clinical studies: A meta-analysis. Clin Oral Implants Res. 2018;29 Suppl 16:135–53.

651. Badr FF, Jadu FM. Performance of artificial intelligence using oral and maxillofacial CBCT images: A systematic review and meta-analysis. Niger J Clin Pract. 2022;25:1918–27.

652. Dini C, Borges GA, Costa RC, Magno MB, Maia LC, Barão VAR. Peri-implant and esthetic outcomes of cemented and screw-retained crowns using zirconia abutments in single implant-supported restorations-A systematic review and meta-analysis. Clin Oral Implants Res. 2021;32:1143–58.

653. de Brandão ML, Vettore MV, Vidigal Júnior GM. Peri-implant bone loss in cement- and screw-retained prostheses: systematic review and meta-analysis. J Clin Periodontol. 2013;40:287–95.

654. Ahmed A, Al-Rasheed A, Badwelan M, Alghamdi HS. Peri-Implant bone response around porous-surface dental implants: A preclinical meta-analysis. Saudi Dent J. 2021;33:239–47.

655. Annibali S, Bignozzi I, Cristalli MP, Graziani F, La Monaca G, Polimeni A. Peri-implant marginal bone level: a systematic review and meta-analysis of studies comparing platform switching versus conventionally restored implants. J Clin Periodontol. 2012;39:1097–113.

656. Baskaradoss JK, Geevarghese A, Baig MR. Peri-implant mucosal response to implant-supported overdentures: A systematic review and meta-analysis. Gerodontology. 2021;38:27–40.

657. Tavelli L, Barootchi S, Avila-Ortiz G, Urban IA, Giannobile WV, Wang H-L. Peri-implant soft tissue phenotype modification and its impact on peri-implant health: A systematic review and network meta-analysis. J Periodontol. 2021;92:21–44.

658. Zhai M, Wang M, Li L, Liu B, Wei F. Periodontal Parameters in Fixed Labial and Lingual Orthodontic Treatment: A Systematic Review and Meta-Analysis. Oral Health Prev Dent. 2022;20:475–84.

659. Chrcanovic BR, Albrektsson T, Wennerberg A. Periodontally compromised vs. periodontally healthy patients and dental implants: a systematic review and meta-analysis. J Dent. 2014;42:1509–27.

660. Sgolastra F, Petrucci A, Severino M, Gatto R, Monaco A. Periodontitis, implant loss and peri-implantitis. A meta-analysis. Clin Oral Implants Res. 2015;26:e8–16.

661. Boccatonda A, Frisone A, Lorusso F, Bugea C, Di Carmine M, Schiavone C, et al. Perioperative Management of Antithrombotic Therapy in Patients Who Undergo Dental Procedures: A Systematic Review of the Literature and Network Meta-Analysis. Int J Environ Res Public Health. 2023;20:5293.

662. Caporossi LS, Dos Santos CS, Calcia TBB, Cenci MS, Muniz FWMG, da Silveira Lima G. Pharmacological management of pain after periodontal surgery: a systematic review with meta-analysis. Clin Oral Investig. 2020;24:2559–78.

663. Angelopoulou MV, Vlachou V, Halazonetis DJ. Pharmacological management of pain during orthodontic treatment: a meta-analysis. Orthod Craniofac Res. 2012;15:71–83.

664. Pesce P, Menini M, Santori G, Giovanni ED, Bagnasco F, Canullo L. Photo and Plasma Activation of Dental Implant Titanium Surfaces. A Systematic Review with Meta-Analysis of Pre-Clinical Studies. J Clin Med. 2020;9:2817.

665. Cronshaw M, Parker S, Anagnostaki E, Mylona V, Lynch E, Grootveld M. Photobiomodulation Dose Parameters in Dentistry: A Systematic Review and Meta-Analysis. Dent J (Basel). 2020;8:114.

666. Reis CLB, de Souza Furtado TC, Mendes WD, Matsumoto MAN, Alves SYF, Stuani MBS, et al. Photobiomodulation impacts the levels of inflammatory mediators during orthodontic tooth movement? A systematic review with meta-analysis. Lasers Med Sci. 2022;37:771–87.

667. Vila-Nova TEL, Leão R de S, Santiago Junior JF, Pellizzer EP, Vasconcelos BC do E, Moraes SLD. Photodynamic therapy in the treatment of denture stomatitis: A systematic review and meta-analysis. J Prosthet Dent. 2023;130:825–32.

668. Pereira TM, Piva E, de Oliveira da Rosa WL, da Silva Nobreza AM, Pivatto K, Aranha AMF, et al. Physicomechanical Properties of Tertiary Monoblock in Endodontics: A Systematic Review and Meta-analysis. Iran Endod J. 2021;16:139–49.

669. Stacchi C, Troiano G, Berton F, Lombardi T, Rapani A, Englaro A, et al. Piezoelectric bone surgery for lateral sinus floor elevation compared with conventional rotary instruments: A systematic review, meta-analysis and trial sequential analysis. Int J Oral Implantol (Berl). 2020;13:109–21.

670. Badenoch-Jones EK, David M, Lincoln T. Piezoelectric compared with conventional rotary osteotomy for the prevention of postoperative sequelae and complications after surgical extraction of mandibular third molars: a systematic review and meta-analysis. Br J Oral Maxillofac Surg. 2016;54:1066–79.

671. Atieh MA, Alsabeeha NHM, Tawse-Smith A, Duncan WJ. Piezoelectric versus conventional implant site preparation: A systematic review and meta-analysis. Clin Implant Dent Relat Res. 2018;20:261–70.

672. Liu J, Hua C, Pan J, Han B, Tang X. Piezosurgery vs conventional rotary instrument in the third molar surgery: A systematic review and meta-analysis of randomized controlled trials. J Dent Sci. 2018;13:342–9.

673. Davidovich E, Shafir S, Shay B, Zini A. Plaque Removal by a Powered Toothbrush Versus a Manual Toothbrush in Children: A Systematic Review and Meta-Analysis. Pediatr Dent. 2020;42:280–7.

674. Gusman D-J-R, Matheus H-R, Alves B-E-S, de Oliveira A-M-P, Britto A-C-DS, Novaes V-C-N, et al. Platelet-rich fibrin for wound healing of palatal donor sites of free gingival grafts: Systematic review and meta-analysis. J Clin Exp Dent. 2021;13:e190–200.

675. Rodas MAR, Paula BL de, Pazmiño VFC, Lot Vieira FFDS, Junior JFS, Silveira EMV. Platelet-Rich Fibrin in Coverage of Gingival Recession: A Systematic Review and Meta-Analysis. Eur J Dent. 2020;14:315–26.

676. Canellas JVDS, Medeiros PJD, Figueredo CMDS, Fischer RG, Ritto FG. Platelet-rich fibrin in oral surgical procedures: a systematic review and meta-analysis. Int J Oral Maxillofac Surg. 2019;48:395–414.

677. Murray PE. Platelet-Rich Plasma and Platelet-Rich Fibrin Can Induce Apical Closure More Frequently Than Blood-Clot Revascularization for the Regeneration of Immature Permanent Teeth: A Meta-Analysis of Clinical Efficacy. Front Bioeng Biotechnol. 2018;6:139.

678. Chrcanovic BR, Albrektsson T, Wennerberg A. Platform switch and dental implants: A meta-analysis. J Dent. 2015;43:629–46.

679. Atieh MA, Ibrahim HM, Atieh AH. Platform switching for marginal bone preservation around dental implants: a systematic review and meta-analysis. J Periodontol. 2010;81:1350–66.

680. Mishra SK, Gaddale R, Sonnahalli NK, Chowdhary R. Platform-Switching Concept in Dental Implants: A Systematic Review and Meta-analysis of Randomized Controlled Trials with a Minimum Follow-up of 3 Years. Int J Oral Maxillofac Implants. 2021;36:e97–109.

681. Santiago JF, Batista VE de S, Verri FR, Honório HM, de Mello CC, Almeida DA dF, et al. Platform-switching implants and bone preservation: a systematic review and meta-analysis. Int J Oral Maxillofac Surg. 2016;45:332–45.

682. Martins CM, da Silva Machado NE, Giopatto BV, de Souza Batista VE, Marsicano JA, Mori GG. Post-operative pain after using sodium hypochlorite and chlorhexidine as irrigation solutions in endodontics: Systematic review and meta-analysis of randomised clinical trials. Indian J Dent Res. 2020;31:774–81.

683. Tennert C, Suárez Machado L, Jaeggi T, Meyer-Lueckel H, Wierichs RJ. Posterior ceramic versus metal restorations: A systematic review and meta-analysis. Dent Mater. 2022;38:1623–32.

684. Bensi C, Belli S, Paradiso D, Lomurno G. Postoperative bleeding risk of direct oral anticoagulants after oral surgery procedures: a systematic review and meta-analysis. Int J Oral Maxillofac Surg. 2018;47:923–32.

685. de Souza GM, Magesty RA, Fernandes IA, Sales KNA, Galvão EL, Falci SGM. Postoperative oral drug regimen to control the inflammatory complications in mandibular third molar surgery: protocol for a systematic review and network meta-analysis. JBI Evid Synth. 2021;19:2024–31.

686. Iocca O, Sollecito TP, Alawi F, Weinstein GS, Newman JG, De Virgilio A, et al. Potentially malignant disorders of the oral cavity and oral dysplasia: A systematic review and meta-analysis of malignant transformation rate by subtype. Head Neck. 2020;42:539–55.

687. Troiano G, Mastrangelo F, Caponio VCA, Laino L, Cirillo N, Lo Muzio L. Predictive Prognostic Value of Tissue-Based MicroRNA Expression in Oral Squamous Cell Carcinoma: A Systematic Review and Meta-analysis. J Dent Res. 2018;97:759–66.

688. Xiao J, Alkhers N, Kopycka-Kedzierawski DT, Billings RJ, Wu TT, Castillo DA, et al. Prenatal Oral Health Care and Early Childhood Caries Prevention: A Systematic Review and Meta-Analysis. Caries Res. 2019;53:411–21.

689. Alves Dos Santos GN, Sousa-Neto MD, Assis HC, Lopes-Olhê FC, Faria-E-Silva AL, Oliveira ML, et al. Prevalence and morphological analysis of dens invaginatus in anterior teeth using cone beam computed tomography: A systematic review and meta-analysis. Arch Oral Biol. 2023;151:105715.

690. Binmadi NO, Alblowi JA. Prevalence and policy of occupational violence against oral healthcare workers: systematic review and meta-analysis. BMC Oral Health. 2019;19:279.

691. Mergoni G, Percudani D, Lodi G, Bertani P, Manfredi M. Prevalence of Candida Species in Endodontic Infections: Systematic Review and Meta-analysis. J Endod. 2018;44:1616-1625.e9.

692. da Silva CG, Pachêco-Pereira C, Porporatti AL, Savi MG, Peres MA, Flores-Mir C, et al. Prevalence of clinical signs of intra-articular temporomandibular disorders in children and adolescents: A systematic review and meta-analysis. J Am Dent Assoc. 2016;147:10-18.e8.

693. Silva MAG, Pantoja LLQ, Dutra-Horstmann KL, Valladares-Neto J, Wolff FL, Porporatti AL, et al. Prevalence of degenerative disease in temporomandibular disorder patients with disc displacement: A systematic review and meta-analysis. J Craniomaxillofac Surg. 2020;48:942–55.

694. Kimmie-Dhansay F, Bhayat A. Prevalence of dental caries in the permanent dentition amongst 12-year-olds in Africa: a systematic review and meta-analysis. BMC Oral Health. 2022;22:453.

695. Oliveira Werlich M, Honnef LR, Silva Bett JV, Domingos FL, Pauletto P, Dulcineia Mendes de Souza B, et al. Prevalence of dentofacial injuries in contact sports players: A systematic review and meta-analysis. Dent Traumatol. 2020;36:477–88.

696. Chenna D, Pentapati KC, Kumar M, Madi M, Siddiq H. Prevalence of musculoskeletal disorders among dental healthcare providers: A systematic review and meta-analysis. F1000Res. 2022;11:1062.

697. Bett JVS, Batistella EÂ, Melo G, Munhoz E de A, Silva CAB, Guerra EN da S, et al. Prevalence of oral mucosal disorders during pregnancy: A systematic review and meta-analysis. J Oral Pathol Med. 2019;48:270–7.

698. Batistella EÂ, Sabino da Silva R, Rivero ERC, Silva CAB. Prevalence of oral mucosal lesions in patients with pemphigus vulgaris: A systematic review and meta-analysis. J Oral Pathol Med. 2021;50:750–7.

699. de Oliveira JMD, Pauletto P, Werlich MO, Massignan C, Lehmkuhl KM, Porfírio GJM, et al. Prevalence of orofacial injuries in wheeled non-motor sports athletes: A systematic review and meta-analysis. Dent Traumatol. 2021;37:546–56.

700. Gambin DJ, Vitali FC, De Carli JP, Mazzon RR, Gomes BPFA, Duque TM, et al. Prevalence of red and orange microbial complexes in endodontic-periodontal lesions: a systematic review and meta-analysis. Clin Oral Investig. 2021;25:6533–46.

701. Valesan LF, Da-Cas CD, Réus JC, Denardin ACS, Garanhani RR, Bonotto D, et al. Prevalence of temporomandibular joint disorders: a systematic review and meta-analysis. Clin Oral Investig. 2021;25:441–53.

702. Singh R, Lehl G, Hussain AB, Abhang TN, Kulkarni MM, Elagib MFA, et al. Prevalence of Titanium Hypersensitivity in Patients with Titanium Implants: A Systematic Review and Meta-analysis. J Pharm Bioallied Sci. 2021;13:S1345–9.

703. Lee C-T, Huang Y-W, Zhu L, Weltman R. Prevalences of peri-implantitis and peri-implant mucositis: systematic review and meta-analysis. J Dent. 2017;62:1–12.

704. Yu X, Chang C, Guo W, Wu Y, Zhou W, Yu D. Primary implant stability based on alternative site preparation techniques: A systematic review and meta-analysis. Clin Implant Dent Relat Res. 2022;24:580–90.

705. Carra MC, Blanc-Sylvestre N, Courtet A, Bouchard P. Primordial and primary prevention of peri-implant diseases: A systematic review and meta-analysis. J Clin Periodontol. 2023;50 Suppl 26:77–112.

706. Oliveira JA, de Oliveira Alves R, Nascimento IM, Hidalgo MAR, Scarel-Caminaga RM, Cristina Pigossi S. Pro- and anti-inflammatory cytokines and osteoclastogenesis-related factors in peri-implant diseases: systematic review and meta-analysis. BMC Oral Health. 2023;23:420.

707. Gruner D, Paris S, Schwendicke F. Probiotics for managing caries and periodontitis: Systematic review and meta-analysis. J Dent. 2016;48:16–25.

708. Radi IA-W, Ibrahim W, Iskandar SMS, AbdelNabi N. Prognosis of dental implants in patients with low bone density: A systematic review and meta-analysis. J Prosthet Dent. 2018;120:668–77.

709. Scelza P, Gonçalves F, Caldas I, Nunes F, Lourenço ES, Tavares S, et al. Prognosis of Regenerative Endodontic Procedures in Mature Teeth: A Systematic Review and Meta-Analysis of Clinical and Radiographic Parameters. Materials (Basel). 2021;14:4418.

710. Chrcanovic BR, Albrektsson T, Wennerberg A. Prophylactic antibiotic regimen and dental implant failure: a meta-analysis. J Oral Rehabil. 2014;41:941–56.

711. Braun RS, Chambrone L, Khouly I. Prophylactic antibiotic regimens in dental implant failure: A systematic review and meta-analysis. J Am Dent Assoc. 2019;150:e61–91.

712. Manicone PF, De Angelis P, Rella E, Papetti L, D’Addona A. Proximal Contact Loss in Implant-Supported Restorations: A Systematic Review and Meta-Analysis of Prevalence. J Prosthodont. 2022;31:201–9.

713. Kailasam V, Rangarajan H, Easwaran HN, Muthu MS. Proximal enamel thickness of the permanent teeth: A systematic review and meta-analysis. Am J Orthod Dentofacial Orthop. 2021;160:793-804.e3.

714. Kohli N, Hugar SM, Soneta SP, Saxena N, Kadam KS, Gokhale N. Psychological behavior management techniques to alleviate dental fear and anxiety in 4-14-year-old children in pediatric dentistry: A systematic review and meta-analysis. Dent Res J (Isfahan). 2022;19:47.

715. Basso IB, Gonçalves FM, Martins AA, Schroder AGD, Taveira KVM, Stechman-Neto J, et al. Psychosocial changes in patients submitted to orthodontic surgery treatment: a systematic review and meta-analysis. Clin Oral Investig. 2022;26:2237–51.

716. Scheerman JFM, van Loveren C, van Meijel B, Dusseldorp E, Wartewig E, Verrips GHW, et al. Psychosocial correlates of oral hygiene behaviour in people aged 9 to 19 - a systematic review with meta-analysis. Community Dent Oral Epidemiol. 2016;44:331–41.

717. Saikiran KV, Gurunathan D, Elicherla SR, Mallineni SK, Nuvvula S. Pulp oxygen saturation measurement as a diagnostic tool for assessing pulp status in primary teeth: A systematic review and meta-analysis. J Indian Soc Pedod Prev Dent. 2022;40:349–55.

718. Barbosa MG, Franco A, de Oliveira RDB, Mamani MP, Junqueira JLC, Soares MQS. Pulp volume quantification methods in cone-beam computed tomography for age estimation: A critical review and meta-analysis. J Forensic Sci. 2023;68:743–56.

719. Lampl S, Gurunathan D, Krithikadatta J, Mehta D, Moodley D. Reasons for Failure of CAD/CAM Restorations in Clinical Studies: A Systematic Review and Meta-analysis. J Contemp Dent Pract. 2023;24:129–36.

720. Martins CM, De Souza Batista VE, Andolfatto Souza AC, Andrada AC, Mori GG, Gomes Filho JE. Reciprocating kinematics leads to lower incidences of postoperative pain than rotary kinematics after endodontic treatment: A systematic review and meta-analysis of randomized controlled trial. J Conserv Dent. 2019;22:320–31.

721. Lambert P, Miguens SAQ, Solda C, Sganzerla JT, Reichert LA, Estrela C, et al. Reference values for pulp oxygen saturation as a diagnostic tool in endodontics: a systematic review and meta-analysis. Restor Dent Endod. 2020;45:e48.

722. Shaik I, Tulli M, Unnam P, Karunakaran S, Vaddi DS, Jabeen R, et al. Regenerative Endodontic Therapy in the Management of Nonvital Immature Permanent teeth: A Systematic Review and Meta-analysis. J Pharm Bioallied Sci. 2021;13:S36–42.

723. Tan N, Sabalic M, Nguyen L, D’Aiuto F. Regenerative Potential of Granulation Tissue in Periodontitis: A Systematic Review and Meta-analysis. Stem Cells Int. 2023;2023:8789852.

724. Arakawa-Kaneko I, Watarai Y, Schimmel M, Abou-Ayash S. Relationship between tongue pressure and handgrip strength: A systematic review and meta-analysis. J Oral Rehabil. 2022;49:1087–105.

725. Andrade RNM, Vieira W de A, Bernardino Í de M, Franco A, Paranhos LR. Reliability of palatal rugoscopy for sexual dimorphism in forensic dentistry: A systematic literature review and meta-analysis. Arch Oral Biol. 2019;97:25–34.

726. Mohammed H, Rizk MZ, Wafaie K, Ulhaq A, Almuzian M. Reminders improve oral hygiene and adherence to appointments in orthodontic patients: a systematic review and meta-analysis. Eur J Orthod. 2019;41:204–13.

727. Kessler SQS, Lang PM, Dal-Pizzol TS, Montagner F. Resistance profiles to antifungal agents in Candida albicans isolated from human oral cavities: systematic review and meta-analysis. Clin Oral Investig. 2022;26:6479–89.

728. Innes NPT, Schwendicke F. Restorative Thresholds for Carious Lesions: Systematic Review and Meta-analysis. J Dent Res. 2017;96:501–8.

729. Jain JK, Sethuraman R, Chauhan S, Javiya P, Srivastava S, Patel R, et al. Retention failures in cement- and screw-retained fixed restorations on dental implants in partially edentulous arches: A systematic review with meta-analysis. J Indian Prosthodont Soc. 2018;18:201–11.

730. Casaña-Ruiz MD, Bellot-Arcís C, Paredes-Gallardo V, García-Sanz V, Almerich-Silla JM, Montiel-Company JM. Risk factors for orthodontic mini-implants in skeletal anchorage biological stability: a systematic literature review and meta-analysis. Sci Rep. 2020;10:5848.

731. Stacchi C, Berton F, Perinetti G, Frassetto A, Lombardi T, Khoury A, et al. Risk Factors for Peri-Implantitis: Effect of History of Periodontal Disease and Smoking Habits. A Systematic Review and Meta-Analysis. J Oral Maxillofac Res. 2016;7:e3.

732. Zou L, Hua L. Risk of bleeding with dental implant surgery in patients on anticoagulant or antiplatelet drugs: a systematic review and meta-analysis. Acta Odontol Scand. 2023;81:98–104.

733. Clauser T, Lin G-H, Lee E, Del Fabbro M, Wang H-L, Testori T. Risk of early implant failure in grafted and non-grafted sites: A systematic review and meta-analysis. Int J Oral Implantol (Berl). 2022;15:31–41.

734. Mendes LT, Pedrotti D, Casagrande L, Lenzi TL. Risk of failure of repaired versus replaced defective direct restorations in permanent teeth: a systematic review and meta-analysis. Clin Oral Investig. 2022;26:4917–27.

735. Chew RJJ, Lu JX, Sim YF, Yeo ABK. Rodent peri-implantitis models: a systematic review and meta-analysis of morphological changes. J Periodontal Implant Sci. 2022;52:479–95.

736. Ghosh M, Gupta R, Jain RA, Mehra R, Verma M. Role and influence of growth factors on early osseointegration in animal jaw bone: A meta-analysis. J Indian Prosthodont Soc. 2020;20:153–61.

737. Fang X, Qi R, Liu C. Root resorption in orthodontic treatment with clear aligners: A systematic review and meta-analysis. Orthod Craniofac Res. 2019;22:259–69.

738. Magesty RA, Galvão EL, de Castro Martins C, Dos Santos CRR, Falci SGM. Rotary Instrument or Piezoelectric for the Removal of Third Molars: a Meta-Analysis. J Maxillofac Oral Surg. 2017;16:13–21.

739. Iliadi A, Koletsi D, Papageorgiou SN, Eliades T. Safety Considerations for Thermoplastic-Type Appliances Used as Orthodontic Aligners or Retainers. A Systematic Review and Meta-Analysis of Clinical and In-Vitro Research. Materials (Basel). 2020;13:1843.

740. Vale F, Correia L, Guimarães A, Caramelo F, Francisco I. Salivary counts of Streptococcus mutans and Lactobacillus in patients with and without cleft lip and/or palate undergoing orthodontic treatment: A meta-analysis. Int J Dent Hyg. 2022;20:112–9.

741. Czumbel LM, Kerémi B, Gede N, Mikó A, Tóth B, Csupor D, et al. Sandblasting reduces dental implant failure rate but not marginal bone level loss: A systematic review and meta-analysis. PLoS One. 2019;14:e0216428.

742. Starch-Jensen T, Nielsen HB. Sandwich osteotomy of the atrophic posterior mandible with interpositional autogenous bone block graft compared with bone substitute material: a systematic review and meta-analysis. Br J Oral Maxillofac Surg. 2020;58:e237–47.

743. Bera RN, Tandon S, Singh AK, Bhattacharjee B, Pandey S, Chirakkattu T. Sandwich osteotomy with interpositional grafts for vertical augmentation of the mandible: A meta-analysis. Natl J Maxillofac Surg. 2022;13:347–56.

744. Kim AJ, Lo AJ, Pullin DA, Thornton-Johnson DS, Karimbux NY. Scaling and root planing treatment for periodontitis to reduce preterm birth and low birth weight: a systematic review and meta-analysis of randomized controlled trials. J Periodontol. 2012;83:1508–19.

745. Starch-Jensen T, Christensen A-E, Lorenzen H. Scalloped Implant-Abutment Connection Compared to Conventional Flat Implant-Abutment Connection: a Systematic Review and Meta-Analysis. J Oral Maxillofac Res. 2017;8:e2.

746. Tedesco TK, Gimenez T, Floriano I, Montagner AF, Camargo LB, Calvo AFB, et al. Scientific evidence for the management of dentin caries lesions in pediatric dentistry: A systematic review and network meta-analysis. PLoS One. 2018;13:e0206296.

747. Fleming PS, Johal A, Pandis N. Self-etch primers and conventional acid-etch technique for orthodontic bonding: a systematic review and meta-analysis. Am J Orthod Dentofacial Orthop. 2012;142:83–94.

748. Edibam NR, Lorenzo-Pouso AI, Caponio VCA. Self-reported allergy to penicillin and clindamycin administration may be risk factors for dental implant failure: A systematic review, meta-analysis and delabeling protocol. Clin Oral Implants Res. 2023;34:651–61.

749. Muniz FWMG, Maurique LS, Toniazzo MP, Silva CF, Casarin M. Self-reported depressive symptoms in dental students: Systematic review with meta-analysis. J Dent Educ. 2021;85:135–47.

750. Fakhruddin KS, Haiat A, Ngo HC, Panduwawala C, Chang JWW, Samaranayake LP. Severe acute respiratory syndrome coronavirus-2 (SARS-CoV-2) viral positivity and their burden in saliva of asymptomatic carriers - a systematic review and meta-analysis. Acta Odontol Scand. 2022;80:182–90.

751. Haridoss S, R B, Swaminathan K, P A. Shaping Properties and Outcomes of Nickel-Titanium Reciprocation Systems in Primary Teeth: A Systematic Review and Meta-Analysis of In Vitro Studies. Cureus. 2022;14:e30995.

752. Altaib FH, Alqutaibi AY, Al-Fahd A, Eid S. Short dental implant as alternative to long implant with bone augmentation of the atrophic posterior ridge: a systematic review and meta-analysis of RCTs. Quintessence Int. 2019;50:636–50.

753. Uehara PN, Matsubara VH, Igai F, Sesma N, Mukai MK, Araujo MG. Short Dental Implants (≤7mm) Versus Longer Implants in Augmented Bone Area: A Meta-Analysis of Randomized Controlled Trials. Open Dent J. 2018;12:354–65.

754. Lemos CAA, Ferro-Alves ML, Okamoto R, Mendonça MR, Pellizzer EP. Short dental implants versus standard dental implants placed in the posterior jaws: A systematic review and meta-analysis. J Dent. 2016;47:8–17.

755. Nielsen HB, Schou S, Isidor F, Christensen A-E, Starch-Jensen T. Short implants (≤8mm) compared to standard length implants (>8mm) in conjunction with maxillary sinus floor augmentation: a systematic review and meta-analysis. Int J Oral Maxillofac Surg. 2019;48:239–49.

756. Sáenz-Ravello G, Ossandón-Zúñiga B, Muñoz-Meza V, Mora-Ferraro D, Baeza M, Fan S, et al. Short implants compared to regular dental implants after bone augmentation in the atrophic posterior mandible: umbrella review and meta-analysis of success outcomes. Int J Implant Dent. 2023;9:18.

757. de N Dias FJ, Pecorari VGA, Martins CB, Del Fabbro M, Casati MZ. Short implants versus bone augmentation in combination with standard-length implants in posterior atrophic partially edentulous mandibles: systematic review and meta-analysis with the Bayesian approach. Int J Oral Maxillofac Surg. 2019;48:90–6.

758. Cruz RS, Lemos CA de A, Batista VE de S, Oliveira HFFE, Gomes JM de L, Pellizzer EP, et al. Short implants versus longer implants with maxillary sinus lift. A systematic review and meta-analysis. Braz Oral Res. 2018;32:e86.

759. Bitinas D, Bardijevskyt G. Short implants without bone augmentation vs. long implants with bone augmentation: systematic review and meta-analysis. Aust Dent J. 2021;66 Suppl 1:S71–81.

760. Toledano M, Fernández-Romero E, Vallecillo C, Toledano R, Osorio MT, Vallecillo-Rivas M. Short versus standard implants at sinus augmented sites: a systematic review and meta-analysis. Clin Oral Investig. 2022;26:6681–98.

761. Dab S, Chen K, Flores-Mir C. Short- and long-term potential effects of accelerated osteogenic orthodontic treatment: A systematic review and meta-analysis. Orthod Craniofac Res. 2019;22:61–8.

762. Grandjean M-L, Maccarone NR, McKenna G, Müller F, Srinivasan M. Silver Diamine Fluoride (SDF) in the management of root caries in elders: a systematic review and meta-analysis. Swiss Dent J. 2021;131:417–24.

763. Wakhloo T, Reddy SG, Sharma SK, Chug A, Dixit A, Thakur K. Silver Diamine Fluoride Versus Atraumatic Restorative Treatment in Pediatric Dental Caries Management: A Systematic Review and Meta-analysis. J Int Soc Prev Community Dent. 2021;11:367–75.

764. Alsilani R, Jadu F, Bogari DF, Jan AM, Alhazzazi TY. Single file reciprocating systems: A systematic review and meta-analysis of the literature: Comparison of reciproc and WaveOne. J Int Soc Prev Community Dent. 2016;6:402–9.

765. Afrashtehfar KI, Katsoulis J, Koka S, Igarashi K. Single versus splinted short implants at sinus augmented sites: A systematic review and meta-analysis. J Stomatol Oral Maxillofac Surg. 2021;122:303–10.

766. Schwendicke F, Göstemeyer G. Single-visit or multiple-visit root canal treatment: systematic review, meta-analysis and trial sequential analysis. BMJ Open. 2017;7:e013115.

767. Klijn RJ, Meijer GJ, Bronkhorst EM, Jansen JA. Sinus floor augmentation surgery using autologous bone grafts from various donor sites: a meta-analysis of the total bone volume. Tissue Eng Part B Rev. 2010;16:295–303.

768. Rongo R, D’Antò V, Bucci R, Polito I, Martina R, Michelotti A. Skeletal and dental effects of Class III orthopaedic treatment: a systematic review and meta-analysis. J Oral Rehabil. 2017;44:545–62.

769. Chrcanovic BR, Albrektsson T, Wennerberg A. Smoking and dental implants: A systematic review and meta-analysis. J Dent. 2015;43:487–98.

770. Mustapha AD, Salame Z, Chrcanovic BR. Smoking and Dental Implants: A Systematic Review and Meta-Analysis. Medicina (Kaunas). 2021;58:39.

771. Chen H, Liu N, Xu X, Qu X, Lu E. Smoking, radiotherapy, diabetes and osteoporosis as risk factors for dental implant failure: a meta-analysis. PLoS One. 2013;8:e71955.

772. Schwendicke F, Dörfer CE, Schlattmann P, Foster Page L, Thomson WM, Paris S. Socioeconomic inequality and caries: a systematic review and meta-analysis. J Dent Res. 2015;94:10–8.

773. Salem AS, Mowafey B, El-Negoly SA, Grawish ME. Socket-shield Technique vs Conventional Immediate Implant Placement for Esthetic Rehabilitation: A Systematic Review and Meta-analysis. J Contemp Dent Pract. 2022;23:237–44.

774. Atieh MA, Alsabeeha NHM. Soft tissue changes after connective tissue grafts around immediately placed and restored dental implants in the esthetic zone: A systematic review and meta-analysis. J Esthet Restor Dent. 2020;32:280–90.

775. Konstantonis D, Vasileiou D, Papageorgiou SN, Eliades T. Soft tissue changes following extraction vs. nonextraction orthodontic fixed appliance treatment: a systematic review and meta-analysis. Eur J Oral Sci. 2018;126:167–79.

776. Atieh MA, Alsabeeha N, Duncan WJ. Stability of tapered and parallel-walled dental implants: A systematic review and meta-analysis. Clin Implant Dent Relat Res. 2018;20:634–45.

777. Ramaglia L, Guida A, Iorio-Siciliano V, Cuozzo A, Blasi A, Sculean A. Stage-specific therapeutic strategies of medication-related osteonecrosis of the jaws: a systematic review and meta-analysis of the drug suspension protocol. Clin Oral Investig. 2018;22:597–615.

778. Fawzy El-Sayed KM, Ahmed GM, Abouauf EA, Schwendicke F. Stem/progenitor cell-mediated pulpal tissue regeneration: a systematic review and meta-analysis. Int Endod J. 2019;52:1573–85.

779. Moraschini V, Barboza E dS P. Success of dental implants in smokers and non-smokers: a systematic review and meta-analysis. Int J Oral Maxillofac Surg. 2016;45:205–15.

780. Rossit M, Gil-Manich V, Ribera-Uribe JM. Success rate of nitrous oxide-oxygen procedural sedation in dental patients: systematic review and meta-analysis. J Dent Anesth Pain Med. 2021;21:527–45.

781. Baima G, Citterio F, Romandini M, Romano F, Mariani GM, Buduneli N, et al. Surface decontamination protocols for surgical treatment of peri-implantitis: A systematic review with meta-analysis. Clin Oral Implants Res. 2022;33:1069–86.

782. Wei H, Liu Z, Zang J, Wang X. Surgery-first/early-orthognathic approach may yield poorer postoperative stability than conventional orthodontics-first approach: a systematic review and meta-analysis. Oral Surg Oral Med Oral Pathol Oral Radiol. 2018;126:107–16.

783. Cairo F, Barbato L, Selvaggi F, Baielli MG, Piattelli A, Chambrone L. Surgical procedures for soft tissue augmentation at implant sites. A systematic review and meta-analysis of randomized controlled trials. Clin Implant Dent Relat Res. 2019;21:1262–70.

784. Daugela P, Cicciù M, Saulacic N. Surgical Regenerative Treatments for Peri-Implantitis: Meta-analysis of Recent Findings in a Systematic Literature Review. J Oral Maxillofac Res. 2016;7:e15.

785. de Araujo CM, Trannin PD, Schroder AGD, Stechman-Neto J, Cavalcante-Leão BL, Mattos NHR, et al. Surgical-Periodontal aspects in orthodontic traction of palatally displaced canines: a meta-analysis. Jpn Dent Sci Rev. 2020;56:164–76.

786. Lee C-T, Chen Y-W, Starr JR, Chuang S-K. Survival analysis of wide dental implant: systematic review and meta-analysis. Clin Oral Implants Res. 2016;27:1251–64.

787. La Monaca G, Pranno N, Annibali S, Massimo C, Polimeni A, Patini R, et al. Survival and complication rates of tooth-implant versus freestanding implant supporting fixed partial prosthesis: a systematic review and meta-analysis. J Prosthodont Res. 2021;65:1–10.

788. Toneatti DJ, Graf RR, Burkhard J-P, Schaller B. Survival of dental implants and occurrence of osteoradionecrosis in irradiated head and neck cancer patients: a systematic review and meta-analysis. Clin Oral Investig. 2021;25:5579–93.

789. Kende PP, Ranganath S, Landge JS, Sarda A, Wadewale M, Patil A, et al. Survival of Dental Implants on Irradiated Jaws: A Systematic Review and Meta-analysis. J Maxillofac Oral Surg. 2022;21:787–95.

790. Medikeri RS, Pereira MA, Waingade M, Navale S. Survival of surface-modified short versus long implants in complete or partially edentulous patients with a follow-up of 1 year or more: a systematic review and meta-analysis. J Periodontal Implant Sci. 2022;52:261–81.

791. Andrade CAS, Paz JLC, de Melo GS, Mahrouseh N, Januário AL, Capeletti LR. Survival rate and peri-implant evaluation of immediately loaded dental implants in individuals with type 2 diabetes mellitus: a systematic review and meta-analysis. Clin Oral Investig. 2022;26:1797–810.

792. Gutiérrez Muñoz D, Obrador Aldover C, Zubizarreta-Macho Á, González Menéndez H, Lorrio Castro J, Peñarrocha-Oltra D, et al. Survival Rate and Prosthetic and Sinus Complications of Zygomatic Dental Implants for the Rehabilitation of the Atrophic Edentulous Maxilla: A Systematic Review and Meta-Analysis. Biology (Basel). 2021;10:601.

793. Shah D, Chauhan C, Shah R. Survival rate of dental implant placed using various maxillary sinus floor elevation techniques: A systematic review and meta-analysis. J Indian Prosthodont Soc. 2022;22:215–24.

794. von Stein-Lausnitz M, Nickenig H-J, Wolfart S, Neumann K, von Stein-Lausnitz A, Spies BC, et al. Survival rates and complication behaviour of tooth implant-supported, fixed dental prostheses: A systematic review and meta-analysis. J Dent. 2019;88:103167.

795. Srinivasan M, Vazquez L, Rieder P, Moraguez O, Bernard J-P, Belser UC. Survival rates of short (6 mm) micro-rough surface implants: a review of literature and meta-analysis. Clin Oral Implants Res. 2014;25:539–45.

796. Papaspyridakos P, De Souza A, Vazouras K, Gholami H, Pagni S, Weber H-P. Survival rates of short dental implants (≤6 mm) compared with implants longer than 6 mm in posterior jaw areas: A meta-analysis. Clin Oral Implants Res. 2018;29 Suppl 16:8–20.

797. Li Q-L, Yao M-F, Cao R-Y, Zhao K, Wang X-D. Survival Rates of Splinted and Nonsplinted Prostheses Supported by Short Dental Implants (≤8.5 mm): A Systematic Review and Meta-Analysis. J Prosthodont. 2022;31:9–21.

798. Lee S-A, Lee C-T, Fu MM, Elmisalati W, Chuang S-K. Systematic review and meta-analysis of randomized controlled trials for the management of limited vertical height in the posterior region: short implants (5 to 8 mm) vs longer implants (> 8 mm) in vertically augmented sites. Int J Oral Maxillofac Implants. 2014;29:1085–97.

799. Keeper JH, Kibbe LJ, Thakkar-Samtani M, Heaton LJ, Desrosiers C, Vela K, et al. Systematic review and meta-analysis on the effect of self-assembling peptide P11-4 on arrest, cavitation, and progression of initial caries lesions. J Am Dent Assoc. 2023;154:580-591.e11.

800. Smiley CJ, Tracy SL, Abt E, Michalowicz BS, John MT, Gunsolley J, et al. Systematic review and meta-analysis on the nonsurgical treatment of chronic periodontitis by means of scaling and root planing with or without adjuncts. J Am Dent Assoc. 2015;146:508-524.e5.

801. Mihit Mihit FZ, Zubizarreta-Macho Á, Montiel-Company JM, Albaladejo Martínez A. Systematic review and network meta-analysis of the accuracy of the orthodontic mini-implants placed in the inter-radicular space by image-guided-based techniques. BMC Oral Health. 2023;23:383.

802. Roca-Millan E, Estrugo-Devesa A, Merlos A, Jané-Salas E, Vinuesa T, López-López J. Systemic Antibiotic Prophylaxis to Reduce Early Implant Failure: A Systematic Review and Meta-Analysis. Antibiotics (Basel). 2021;10:698.

803. Kherul Anuwar AH, Saub R, Safii SH, Ab-Murat N, Mohd Taib MS, Mamikutty R, et al. Systemic Antibiotics as an Adjunct to Subgingival Debridement: A Network Meta-Analysis. Antibiotics (Basel). 2022;11:1716.

804. Clementini M, Rossetti PHO, Penarrocha D, Micarelli C, Bonachela WC, Canullo L. Systemic risk factors for peri-implant bone loss: a systematic review and meta-analysis. Int J Oral Maxillofac Surg. 2014;43:323–34.

805. Kanzow P, Lechte C, Wiegand A, Wilson NHF, Lynch CD, Blum IR. Teaching of posterior composites for the restoration of permanent teeth in undergraduate dental training programmes: Systematic review and meta-analysis. J Dent. 2023;135:104589.

806. Fernández CE, Maturana CA, Coloma SI, Carrasco-Labra A, Giacaman RA. Teledentistry and mHealth for Promotion and Prevention of Oral Health: A Systematic Review and Meta-analysis. J Dent Res. 2021;100:914–27.

807. Uhrin E, Domokos Z, Czumbel LM, Kói T, Hegyi P, Hermann P, et al. Teledentistry: A Future Solution in the Diagnosis of Oral Lesions: Diagnostic Meta-Analysis and Systematic Review. Telemed J E Health. 2023;29:1591–600.

808. Tahmaseb A, Wu V, Wismeijer D, Coucke W, Evans C. The accuracy of static computer-aided implant surgery: A systematic review and meta-analysis. Clin Oral Implants Res. 2018;29 Suppl 16:416–35.

809. Arraj GP, Rossi-Fedele G, Doğramacı EJ. The association of overjet size and traumatic dental injuries-A systematic review and meta-analysis. Dent Traumatol. 2019;35:217–32.

810. Rabel K, Spies BC, Pieralli S, Vach K, Kohal R-J. The clinical performance of all-ceramic implant-supported single crowns: A systematic review and meta-analysis. Clin Oral Implants Res. 2018;29 Suppl 18:196–223.

811. Gheisary Z, Mahmood R, Harri Shivanantham A, Liu J, Lieffers JRL, Papagerakis P, et al. The Clinical, Microbiological, and Immunological Effects of Probiotic Supplementation on Prevention and Treatment of Periodontal Diseases: A Systematic Review and Meta-Analysis. Nutrients. 2022;14:1036.

812. Ganji K, Rostamzadeh M, Talimkhani I, Moradi Y. The Comparison of Survival Rates between Narrow Diameter Implants and Standard Diameter Implants: An Updated Meta-analysis. Med J Islam Repub Iran. 2023;37:5.

813. Ostrowski P, Bonczar M, Wilk J, Michalczak M, Czaja J, Niziolek M, et al. The complete anatomy of the lingual nerve: A meta-analysis with implications for oral and maxillofacial surgery. Clin Anat. 2023;36:905–14.

814. Perinetti G, Westphalen GH, Biasotto M, Salgarello S, Contardo L. The diagnostic performance of dental maturity for identification of the circumpubertal growth phases: a meta-analysis. Prog Orthod. 2013;14:8.

815. Aulestia-Viera PV, Braga MM, Borsatti MA. The effect of adjusting the pH of local anaesthetics in dentistry: a systematic review and meta-analysis. Int Endod J. 2018;51:862–76.

816. Altmann ASP, Collares FM, Leitune VCB, Samuel SMW. The effect of antimicrobial agents on bond strength of orthodontic adhesives: a meta-analysis of in vitro studies. Orthod Craniofac Res. 2016;19:1–9.

817. Stavropoulos A, Bertl K, Pietschmann P, Pandis N, Schiødt M, Klinge B. The effect of antiresorptive drugs on implant therapy: Systematic review and meta-analysis. Clin Oral Implants Res. 2018;29 Suppl 18:54–92.

818. Youssef M, Marzouk T, Abdelsalam H, Malmstrom H, Barmak AB, Fraser D, et al. The effect of electronic cigarette use on peri-implant conditions in men: a systematic review and meta-analysis. Oral Surg Oral Med Oral Pathol Oral Radiol. 2023;135:492–500.

819. Espinosa DG, Cruz CM da V, Normando D. The effect of extraction of lower primary canines on the morphology of dental arch: A systematic review and meta-analysis. Int J Paediatr Dent. 2021;31:583–97.

820. Ding L, Chen R, Liu J, Wang Y, Chang Q, Ren L. The effect of functional mandibular advancement for adolescent patients with skeletal class II malocclusion on the TMJ: a systematic review and meta-analysis. BMC Oral Health. 2022;22:51.

821. Machtei EE. The effect of membrane exposure on the outcome of regenerative procedures in humans: a meta-analysis. J Periodontol. 2001;72:512–6.

822. Scardini IL, Sarra G, Braga MM, Dos Santos M, Freire LG. The Effect of Number of Visits, Use of Solvent and Gutta-percha Removal Technique on Postoperative Pain following Nonsurgical Endodontic Retreatment; A Systematic Review and Meta-analysis. Iran Endod J. 2023;18:71–84.

823. Kouskoura T, Ochsner T, Verna C, Pandis N, Kanavakis G. The effect of orthodontic treatment on facial attractiveness: a systematic review and meta-analysis. Eur J Orthod. 2022;44:636–49.

824. Dodge A, Garcia J, Luepke P, Lai Y-L, Kassab M, Lin G-H. The effect of partially exposed connective tissue graft on root-coverage outcomes: a systematic review and meta-analysis. Eur J Oral Sci. 2018;126:84–92.

825. Lima Neto JC, Fernandes LM, Magno MB, Rocha Lima TF, de Almeida L de FD, Santiago BM, et al. The Effect of Reciprocating and Rotary Systems on Postoperative Endodontic Pain: A Systematic Review and Meta-analysis. Iran Endod J. 2020;15:198–210.

826. Lee C-T, Hum L, Chen Y-W. The effect of regenerative periodontal therapy in preventing periodontal defects after the extraction of third molars: A systematic review and meta-analysis. J Am Dent Assoc. 2016;147:709-719.e4.

827. Oliveira BH, Rajendra A, Veitz-Keenan A, Niederman R. The Effect of Silver Diamine Fluoride in Preventing Caries in the Primary Dentition: A Systematic Review and Meta-Analysis. Caries Res. 2019;53:24–32.

828. Mohaghegh S, Soleimani M, Kouhestani F, Motamedian SR. The effect of single/multiple micro-osteoperforation on the rate of orthodontic tooth movement and its possible complications: A systematic review and meta-analysis. Int Orthod. 2021;19:183–96.

829. De Angelis P, Manicone PF, Rella E, Liguori MG, De Angelis S, Tancredi S, et al. The effect of soft tissue augmentation on the clinical and radiographical outcomes following immediate implant placement and provisionalization: a systematic review and meta-analysis. Int J Implant Dent. 2021;7:86.

830. Linkevicius T, Vaitelis J. The effect of zirconia or titanium as abutment material on soft peri-implant tissues: a systematic review and meta-analysis. Clin Oral Implants Res. 2015;26 Suppl 11:139–47.

831. Chęcińska K, Chęciński M, Sikora M, Nowak Z, Karwan S, Chlubek D. The Effect of Zirconium Dioxide (ZrO2) Nanoparticles Addition on the Mechanical Parameters of Polymethyl Methacrylate (PMMA): A Systematic Review and Meta-Analysis of Experimental Studies. Polymers (Basel). 2022;14:1047.

832. Fleming PS, Johal A, Pandis N. The effectiveness of laceback ligatures during initial orthodontic alignment: a systematic review and meta-analysis. Eur J Orthod. 2013;35:539–46.

833. Ramli H, Nor Aripin KN, Mohd Said S, Mohamad Hanafiah R, Mohd Dom TN. The effectiveness of miswak (Salvadora persica L. and Azadirachta indica A.Juss.) practices in reducing plaque and gingivitis among adults: A systematic review and meta-analysis. J Ethnopharmacol. 2022;298:115598.

834. Eid Alroudhan I, Gamal M, Ganji KK, Khan AM, Alsharari KN, Alruwaili MK, et al. The Effectiveness of Mouthwashes With Various Ingredients in Plaque Control: A Systematic Review and Meta-Analysis. Altern Ther Health Med. 2021;27:52–7.

835. Li J, Ge X, Guan H, Jia L, Chang W, Ma W. The Effectiveness of Photobiomodulation on Accelerating Tooth Movement in Orthodontics: A Systematic Review and Meta-Analysis. Photobiomodul Photomed Laser Surg. 2021;39:232–44.

836. Alsawaf DH, Almaasarani SG, Hajeer MY, Rajeh N. The effectiveness of the early orthodontic correction of functional unilateral posterior crossbite in the mixed dentition period: a systematic review and meta-analysis. Prog Orthod. 2022;23:5.

837. Willenbacher M, Al-Nawas B, Berres M, Kämmerer PW, Schiegnitz E. The Effects of Alveolar Ridge Preservation: A Meta-Analysis. Clin Implant Dent Relat Res. 2016;18:1248–68.

838. Cheng C, Xie T, Wang J. The efficacy of analgesics in controlling orthodontic pain: a systematic review and meta-analysis. BMC Oral Health. 2020;20:259.

839. Mando M, Talaat S, Bourauel C. The efficacy of chewing gum in the reduction of orthodontic pain at its peak intensity: a systematic review and meta-analysis. Angle Orthod. 2023;93:580–90.

840. Wilensky A, Shapira L, Limones A, Martin C. The efficacy of implant surface decontamination using chemicals during surgical treatment of peri-implantitis: A systematic review and meta-analysis. J Clin Periodontol. 2023;50 Suppl 26:336–58.

841. Ahmad N, Grad HA, Haas DA, Aronson KJ, Jokovic A, Locker D. The efficacy of nonopioid analgesics for postoperative dental pain: a meta-analysis. Anesth Prog. 1997;44:119–26.

842. Park JS, Anthonappa RP, King NM, McGrath CP. The family impact of dental general anaesthesia in children: A meta-analysis. Int J Paediatr Dent. 2018;

843. Fan S, Sáenz-Ravello G, Al-Nawas B, Schiegnitz E, Diaz L, Sagheb K. The feasibility of ultrasonography for the measurement of periodontal and peri-implant phenotype: A systematic review and meta-analysis. Clin Implant Dent Relat Res. 2023;25:892–909.

844. Abdel-Halim M, Issa D, Chrcanovic BR. The Impact of Dental Implant Length on Failure Rates: A Systematic Review and Meta-Analysis. Materials (Basel). 2021;14:3972.

845. Botelho J, Machado V, Proença L, Bellini DH, Chambrone L, Alcoforado G, et al. The impact of nonsurgical periodontal treatment on oral health-related quality of life: a systematic review and meta-analysis. Clin Oral Investig. 2020;24:585–96.

846. Bezerra PMM, Vieira TI, Dos Santos FG, Ribeiro ILA, de Sousa SA, Valença AMG. The impact of oral health education on the incidence and severity of oral mucositis in pediatric cancer patients: a systematic review and meta-analysis. Support Care Cancer. 2022;30:8819–29.

847. Silva Teófilo MÍ, de Carvalho Russi TMAZ, de Barros Silva PG, Balhaddad AA, Melo MAS, Rolim JPML. The Impact of Photosensitizer Selection on Bactericidal Efficacy Of PDT against Cariogenic Biofilms: A Systematic Review and Meta-Analysis. Photodiagnosis Photodyn Ther. 2021;33:102046.

848. Aminoshariae A, Kulild JC. The impact of sealer extrusion on endodontic outcome: A systematic review with meta-analysis. Aust Endod J. 2020;46:123–9.

849. Gupta S, Del Fabbro M, Chang J. The impact of simvastatin intervention on the healing of bone, soft tissue, and TMJ cartilage in dentistry: a systematic review and meta-analysis. Int J Implant Dent. 2019;5:17.

850. Chang J, Meng H-W, Lalla E, Lee C-T. The impact of smoking on non-surgical periodontal therapy: A systematic review and meta-analysis. J Clin Periodontol. 2021;48:60–75.

851. Fouda AAH. The impact of the alveolar bone sites on early implant failure: a systematic review with meta-analysis. J Korean Assoc Oral Maxillofac Surg. 2020;46:162–73.

852. Caricasulo R, Malchiodi L, Ghensi P, Fantozzi G, Cucchi A. The influence of implant-abutment connection to peri-implant bone loss: A systematic review and meta-analysis. Clin Implant Dent Relat Res. 2018;20:653–64.

853. Atieh MA, Baqain ZH, Tawse-Smith A, Ma S, Almoselli M, Lin L, et al. The influence of insertion torque values on the failure and complication rates of dental implants: A systematic review and meta-analysis. Clin Implant Dent Relat Res. 2021;23:341–60.

854. Reis INRD, do Amaral GCLS, Hassan MA, Villar CC, Romito GA, Spin-Neto R, et al. The influence of smoking on the incidence of peri-implantitis: A systematic review and meta-analysis. Clin Oral Implants Res. 2023;34:543–54.

855. Bienz SP, Pirc M, Papageorgiou SN, Jung RE, Thoma DS. The influence of thin as compared to thick peri-implant soft tissues on aesthetic outcomes: A systematic review and meta-analysis. Clin Oral Implants Res. 2022;33 Suppl 23:56–71.

856. Mahardawi B, Jiaranuchart S, Damrongsirirat N, Arunjaroensuk S, Mattheos N, Somboonsavatdee A, et al. The lack of keratinized mucosa as a risk factor for peri-implantitis: a systematic review and meta-analysis. Sci Rep. 2023;13:3778.

857. Musskopf ML, Finger Stadler A, Wikesjö UM, Susin C. The minipig intraoral dental implant model: A systematic review and meta-analysis. PLoS One. 2022;17:e0264475.

858. Manipal S, Hussain S, Wadgave U, Duraiswamy P, Ravi K. The Mouthwash War - Chlorhexidine vs. Herbal Mouth Rinses: A Meta-Analysis. J Clin Diagn Res. 2016;10:ZC81-83.

859. Atieh MA, Tawse-Smith A, Alsabeeha NHM, Ma S, Duncan WJ. The One Abutment-One Time Protocol: A Systematic Review and Meta-Analysis. J Periodontol. 2017;88:1173–85.

860. Ningrum V, Bakar A, Shieh T-M, Shih Y-H. The Oral Health Inequities between Special Needs Children and Normal Children in Asia: A Systematic Review and Meta-Analysis. Healthcare (Basel). 2021;9:410.

861. Hao C-P, Cao N-J, Zhu Y-H, Wang W. The osseointegration and stability of dental implants with different surface treatments in animal models: a network meta-analysis. Sci Rep. 2021;11:13849.

862. Brandt RG, Anderson PF, McDonald NJ, Sohn W, Peters MC. The pulpal anesthetic efficacy of articaine versus lidocaine in dentistry: a meta-analysis. J Am Dent Assoc. 2011;142:493–504.

863. Girijan P, Boedi R, Mânica S, Franco A. The radiographic diversity of dental patterns for human identification - Systematic review and meta-analysis. J Forensic Leg Med. 2023;95:102507.

864. Liu J, Shi Q, Yang S, Wang Q, Xu J, Guo B. The relationship between levels of salivary and serum interleukin-6 and oral lichen planus: A systematic review and meta-analysis. J Am Dent Assoc. 2017;148:743-749.e9.

865. AlMoharib HS, AlRowis R, AlMubarak A, Waleed Almadhoon H, Ashri N. The relationship between matrix metalloproteinases-8 and peri-implantitis: A systematic review and meta-analysis. Saudi Dent J. 2023;35:283–93.

866. Ravidà A, Arena C, Tattan M, Caponio VCA, Saleh MHA, Wang H-L, et al. The role of keratinized mucosa width as a risk factor for peri-implant disease: A systematic review, meta-analysis, and trial sequential analysis. Clin Implant Dent Relat Res. 2022;24:287–300.

867. Gao J, Nguyen T, Oberoi S, Oh H, Kapila S, Kao RT, et al. The Significance of Utilizing A Corticotomy on Periodontal and Orthodontic Outcomes: A Systematic Review and Meta-Analysis. Biology (Basel). 2021;10:803.

868. Atieh MA, Shah M, Abdulkareem M, AlQahtani HA, Alsabeeha NHM. The socket shield technique for immediate implant placement: A systematic review and meta-analysis. J Esthet Restor Dent. 2021;33:1186–200.

869. Waechter J, Leite FR, Nascimento GG, Carmo Filho LC, Faot F. The split crest technique and dental implants: a systematic review and meta-analysis. Int J Oral Maxillofac Surg. 2017;46:116–28.

870. Li X, Lin X, Guo J, Wang Y. The Stability and Survival Rate of Dental Implants After Preparation of the Site by Piezosurgery vs Conventional Drilling: A Systematic Review and Meta-Analysis. Int J Oral Maxillofac Implants. 2020;30:e51–6.

871. Desjardins PJ, Mehlisch DR, Chang DJ, Krupa D, Polis AB, Petruschke RA, et al. The time to onset and overall analgesic efficacy of rofecoxib 50 mg: a meta-analysis of 13 randomized clinical trials. Clin J Pain. 2005;21:241–50.

872. Canullo L, Troiano G, Sbricoli L, Guazzo R, Laino L, Caiazzo A, et al. The Use of Antibiotics in Implant Therapy: A Systematic Review and Meta-Analysis with Trial Sequential Analysis on Early Implant Failure. Int J Oral Maxillofac Implants. 2020;35:485–94.

873. Franchini M, Cruciani M, Mengoli C, Masiello F, Marano G, D’Aloja E, et al. The use of platelet-rich plasma in oral surgery: a systematic review and meta-analysis. Blood Transfus. 2019;17:357–67.

874. Awad ME, Altman A, Elrefai R, Shipman P, Looney S, Elsalanty M. The use of vascularized fibula flap in mandibular reconstruction; A comprehensive systematic review and meta-analysis of the observational studies. J Craniomaxillofac Surg. 2019;47:629–41.

875. Bourgi R, Hardan L, Cuevas-Suárez CE, Scavello F, Mancino D, Kharouf N, et al. The Use of Warm Air for Solvent Evaporation in Adhesive Dentistry: A Meta-Analysis of In Vitro Studies. J Funct Biomater. 2023;14:285.

876. Franco A, Lima LKG, de Oliveira MN, de Andrade Vieira W, Blumenberg C, Costa MM, et al. The weak evidence of lip print analysis for sexual dimorphism in forensic dentistry: a systematic literature review and meta-analysis. Sci Rep. 2021;11:24192.

877. Guo T, Gulati K, Shen Z, Han P, Fan Z. Therapeutic outcomes of non-grafted and platelet concentrations-grafted transcrestal maxillary sinus elevation (TSFE): a systematic review and meta-analysis. Sci Rep. 2020;10:5935.

878. Mesko ME, Hutton B, Skupien JA, Sarkis-Onofre R, Moher D, Pereira-Cenci T. Therapies for bruxism: a systematic review and network meta-analysis (protocol). Syst Rev. 2017;6:4.

879. Glória JCR, Martins CC, Armond ACV, Galvão EL, Dos Santos CRR, Falci SGM. Third Molar and Their Relationship with Caries on the Distal Surface of Second Molar: A Meta-analysis. J Maxillofac Oral Surg. 2018;17:129–41.

880. Niu X, Di Carlo G, Cornelis MA, Cattaneo PM. Three-dimensional analyses of short- and long-term effects of rapid maxillary expansion on nasal cavity and upper airway: A systematic review and meta-analysis. Orthod Craniofac Res. 2020;23:250–76.

881. Chrcanovic BR, Albrektsson T, Wennerberg A. Tilted versus axially placed dental implants: a meta-analysis. J Dent. 2015;43:149–70.

882. Claudy MP, Miguens SAQ, Celeste RK, Camara Parente R, Hernandez PAG, da Silva AN. Time interval after radiotherapy and dental implant failure: systematic review of observational studies and meta-analysis. Clin Implant Dent Relat Res. 2015;17:402–11.

883. Silva EVF, Commar BC, Bitencourt SB, Bonatto LR, Dos Santos DM, Bittencourt ABBC, et al. Titanium versus ceramic implants for overdentures: a meta-analysis of prospective studies. Gen Dent. 2021;69:e1–5.

884. Husain F, Gupta S, Sood S, Bhaskar N, Jain A. To evaluate the effect of anodized dental implant surface on cumulative implant survival and success. A systematic review and meta-analysis. J Indian Soc Periodontol. 2022;26:525–32.

885. Rezende M, Coppla FM, Chemin K, Chibinski AC, Loguercio AD, Reis A. Tooth Sensitivity After Dental Bleaching With a Desensitizer-containing and a Desensitizer-free Bleaching Gel: A Systematic Review and Meta-analysis. Oper Dent. 2019;44:E58–74.

886. Borse S, Chaware SH. Tooth shade analysis and selection in prosthodontics: A systematic review and meta-analysis. J Indian Prosthodont Soc. 2020;20:131–40.

887. de Vasconcellos SJ de A, de Santana Santos T, Reinheimer DM, Faria-E-Silva AL, de Melo M de FB, Martins-Filho PRS. Topical application of tranexamic acid in anticoagulated patients undergoing minor oral surgery: A systematic review and meta-analysis of randomized clinical trials. J Craniomaxillofac Surg. 2017;45:20–6.

888. Manchanda S, Sardana D, Liu P, Lee GH, Li KY, Lo EC, et al. Topical fluoride to prevent early childhood caries: Systematic review with network meta-analysis. J Dent. 2022;116:103885.

889. Zhang J, Sardana D, Li KY, Leung KCM, Lo ECM. Topical Fluoride to Prevent Root Caries: Systematic Review with Network Meta-analysis. J Dent Res. 2020;99:506–13.

890. Barakat D, Bakdach WMM, Youssef M. Treatment effects of Carriere Motion Appliance on patients with class II malocclusion: A systematic review and meta-analysis. Int Orthod. 2021;19:353–64.

891. Vetromilla BM, Opdam NJ, Leida FL, Sarkis-Onofre R, Demarco FF, van der Loo MPJ, et al. Treatment options for large posterior restorations: a systematic review and network meta-analysis. J Am Dent Assoc. 2020;151:614-624.e18.

892. Li J, Zheng L, Daraqel B, Liu J, Hu Y. Treatment Outcome of Regenerative Endodontic Procedures for Necrotic Immature and Mature Permanent Teeth: A Systematic Review and Meta-Analysis Based on Randomised Controlled Trials. Oral Health Prev Dent. 2023;21:141–52.

893. Nangia D, Saini A, Sharma S, Kumar V, Chawla A, Perumal V, et al. Treatment outcome of regenerative endodontic procedures in mature permanent teeth compared to nonsurgical endodontic treatment: A systematic review and meta-analysis. J Conserv Dent. 2021;24:530–8.

894. Chrcanovic BR, Albrektsson T, Wennerberg A. Turned versus anodised dental implants: a meta-analysis. J Oral Rehabil. 2016;43:716–28.

895. Patini R, Gallenzi P, Lione R, Cozza P, Cordaro M. Ultrasonographic Evaluation of The Effects of Orthodontic or Functional Orthopaedic Treatment on Masseter Muscles: A Systematic Review and Meta-Analysis. Medicina (Kaunas). 2019;55:256.

896. Kanzow P, Wiegand A, Göstemeyer G, Schwendicke F. Understanding the management and teaching of dental restoration repair: Systematic review and meta-analysis of surveys. J Dent. 2018;69:1–21.

897. Favoreto MW, de Souza Carneiro T, Forville H, Burey A, Simas Dreweck FD, Loguercio AD, et al. Use of calcium-containing bioactive desensitizers in dental bleaching: A systematic review and meta-analysis. J Am Dent Assoc. 2023;154:245-259.e12.

898. Ghassib I, Chen Z, Zhu J, Wang H-L. Use of IL-1 β, IL-6, TNF-α, and MMP-8 biomarkers to distinguish peri-implant diseases: A systematic review and meta-analysis. Clin Implant Dent Relat Res. 2019;21:190–207.

899. Moraschini V, Barboza E dos SP. Use of Platelet-Rich Fibrin Membrane in the Treatment of Gingival Recession: A Systematic Review and Meta-Analysis. J Periodontol. 2016;87:281–90.

900. Nishi SE, Basri R, Alam MK. Uses of electromyography in dentistry: An overview with meta-analysis. Eur J Dent. 2016;10:419–25.

901. Marconi V, Iommi M, Monachesi C, Faragalli A, Skrami E, Gesuita R, et al. Validity of age estimation methods and reproducibility of bone/dental maturity indices for chronological age estimation: a systematic review and meta-analysis of validation studies. Sci Rep. 2022;12:15607.

902. Casett E, Réus JC, Stuginski-Barbosa J, Porporatti AL, Carra MC, Peres MA, et al. Validity of different tools to assess sleep bruxism: a meta-analysis. J Oral Rehabil. 2017;44:722–34.

903. Arakawa I, Igarashi K, Imamura Y, Müller F, Abou-Ayash S, Schimmel M. Variability in tongue pressure among elderly and young healthy cohorts: A systematic review and meta-analysis. J Oral Rehabil. 2021;48:430–48.

904. Terheyden H, Meijer GJ, Raghoebar GM. Vertical bone augmentation and regular implants versus short implants in the vertically deficient posterior mandible: a systematic review and meta-analysis of randomized studies. Int J Oral Maxillofac Surg. 2021;50:1249–58.

905. Hameed MH, Gul M, Ghafoor R, Khan FR. Vertical Ridge Gain with Various Bone Augmentation Techniques: A Systematic Review and Meta-Analysis. J Prosthodont. 2019;28:421–7.

906. Akbari A, Gandhi V, Chen J, Turkkahraman H, Yadav S. Vibrational Force on Accelerating Orthodontic Tooth Movement: A Systematic Review and Meta-Analysis. Eur J Dent. 2023;17:951–63.

907. Gupta N, Gupta A, Sarma R, Batra A, Madan K. Video laryngoscopy vs. direct laryngoscopy for nasotracheal intubation in oromaxillofacial surgery: a systematic review and meta-analysis of randomized controlled trials. Korean J Anesthesiol. 2021;74:439–48.

908. Schwendicke F, Elhennawy K, El Shahawy O, Maher R, Gimenez T, Mendes FM, et al. Visual and radiographic caries detection: a tailored meta-analysis for two different settings, Egypt and Germany. BMC Oral Health. 2018;18:105.

909. Gimenez T, Piovesan C, Braga MM, Raggio DP, Deery C, Ricketts DN, et al. Visual Inspection for Caries Detection: A Systematic Review and Meta-analysis. J Dent Res. 2015;94:895–904.

910. Buck LM, Dalci O, Darendeliler MA, Papageorgiou SN, Papadopoulou AK. Volumetric upper airway changes after rapid maxillary expansion: a systematic review and meta-analysis. Eur J Orthod. 2017;39:463–73.

911. Kim JS, Choi SM, Yoon JH, Lee EJ, Yoon J, Kwon SH, et al. What Affects Postoperative Sinusitis and Implant Failure after Dental Implant: A Meta-analysis. Otolaryngol Head Neck Surg. 2019;160:974–84.

912. Kyburz KS, Eliades T, Papageorgiou SN. What effect does functional appliance treatment have on the temporomandibular joint? A systematic review with meta-analysis. Prog Orthod. 2019;20:32.

913. Ata-Ali J, Ata-Ali F, Peñarrocha-Oltra D, Galindo-Moreno P. What is the impact of bisphosphonate therapy upon dental implant survival? A systematic review and meta-analysis. Clin Oral Implants Res. 2016;27:e38-46.

914. Diaz P, Gonzalo E, Villagra LJG, Miegimolle B, Suarez MJ. What is the prevalence of peri-implantitis? A systematic review and meta-analysis. BMC Oral Health. 2022;22:449.

915. Gangwani P, Almana M, Barmak B, Kolokythas A. What Is the Success of Implants Placed in Fibula Flap? A Systematic Review and Meta-Analysis. J Oral Maxillofac Res. 2022;13:e3.

916. Canellas JVDS, Medeiros PJD, Figueredo CM da S, Fischer RG, Ritto FG. Which is the best choice after tooth extraction, immediate implant placement or delayed placement with alveolar ridge preservation? A systematic review and meta-analysis. J Craniomaxillofac Surg. 2019;47:1793–802.

917. Soeteman GD, Valkenburg C, Van der Weijden GA, Van Loveren C, Bakker E, Slot DE. Whitening dentifrice and tooth surface discoloration-a systematic review and meta-analysis. Int J Dent Hyg. 2018;16:24–35.

918. Manfredi M, Dave B, Percudani D, Christoforou J, Karasneh J, Diz Dios P, et al. World workshop on oral medicine VII: Direct anticoagulant agents management for invasive oral procedures: A systematic review and meta-analysis. Oral Dis. 2019;25 Suppl 1:157–73.

919. Martins JNR, Nole C, Ounsi HF, Parashos P, Plotino G, Ragnarsson MF, et al. Worldwide Assessment of the Mandibular First Molar Second Distal Root and Root Canal: A Cross-sectional Study with Meta-analysis. J Endod. 2022;48:223–33.

920. Pereira R da PL, de Oliveira JMD, Pauletto P, Munhoz E de A, Silva Guerra EN, Massignan C, et al. Worldwide prevalence of geographic tongue in adults: A systematic review and meta-analysis. Oral Dis. 2023;29:3091–100.

921. González-Moles MÁ, Warnakulasuriya S, González-Ruiz I, González-Ruiz L, Ayén Á, Lenouvel D, et al. Worldwide prevalence of oral lichen planus: A systematic review and meta-analysis. Oral Dis. 2021;27:813–28.

922. Martins JNR, Worldwide Anatomy Research Group, Versiani MA. Worldwide Prevalence of the Lingual Canal in Mandibular Incisors: A Multicenter Cross-sectional Study with Meta-analysis. J Endod. 2023;49:819–35.

923. Roehling S, Schlegel KA, Woelfler H, Gahlert M. Zirconia compared to titanium dental implants in preclinical studies-A systematic review and meta-analysis. Clin Oral Implants Res. 2019;30:365–95.

924. Borges H, Correia ARM, Castilho RM, de Oliveira Fernandes GV. Zirconia Implants and Marginal Bone Loss: A Systematic Review and Meta-Analysis of Clinical Studies. Int J Oral Maxillofac Implants. 2020;35:707–20.

**2.List of excluded articles after screening the abstract and individual reasons for exclusion**

1. Zhang X, Tian X, Wei Y, Deng H, Ma L, Chen Z. Activity and Safety of Tegafur, Gimeracil, and Oteracil Potassium for Nasopharyngeal Carcinoma: A Systematic Review and Meta-Analysis. J Oncol. 2021;2021:6690275.

Reason for exclusion: Article is not related to dentistry.

2. Jacometti V, Sato CM, Meireles DA, Silva RHA da. Age estimation using London Atlas methodology: A systematic review and meta-analysis. Forensic Sci Int. 2023;342:111532.

Reason for exclusion: Article is not related to the topic.

3. Seehra J, Stonehouse-Smith D, Pandis N. Assessment of early exaggerated treatment effects in orthodontic interventions using cumulative meta-analysis. Eur J Orthod. 2021;43:601–5.

Reason for exclusion: Article is not a review.

4. Liang M, Lian Q, Kotsakis GA, Michalowicz BS, John MT, Chu H. Bayesian Network Meta-analysis of Multiple Outcomes in Dental Research. J Evid Based Dent Pract. 2020;20:101403.

Reason for exclusion: Article is not a review.

5. Mupparapu M, Nath S. Calcified carotid artery atheroma and stroke risk assessment. Use of Doppler ultrasonography as a secondary marker: a meta-analysis. Quintessence Int. 2021;52:348–59.

Reason for exclusion: Article is not related to dentistry.

6. Zhang J, Xu J, Zhang J, Ren Y. Chinese herbal compound combined with western medicine therapy in the treatment of plasma cell mastitis: A protocol for systematic review and meta-analysis. Medicine (Baltimore). 2020;99:e22858.

Reason for exclusion: Article is not related to dentistry.

7. Wu Z-X, Cai M-J, Huang P-D, Chen J-Y, Lv Z-H, Huang X-Y. Comparative efficacy and dysmenorrhea score of 6 object-separated moxibustions for the treatment of Chinese patients with dysmenorrhea: A systematic review and network meta-analysis. Medicine (Baltimore). 2021;100:e26185.

Reason for exclusion: Article is not related to dentistry.

8. Zhang Y, Ma H, Nan T, Li Y, Zheng W, Zhou Z, et al. Comparative Efficacy of Oral Chinese Patent Medicine for Chronic Prostatitis/Chronic Pelvic Pain Syndrome With Sexual Dysfunction: A Bayesian Network Meta-Analysis of Randomized Controlled Trials. Front Pharmacol. 2021;12:649470.

Reason for exclusion: Article is not related to dentistry.

9. Pinto A. Considerations for planning and designing meta-analysis in oral medicine. Oral Surg Oral Med Oral Pathol Oral Radiol. 2013;116:194–202.

Reason for exclusion: Article is either an opinion paper, Editorial, Letter to the Editor, Response to published article or correspondence.

10. Martin E, Nimmo A, Lee A, Jennings E. Correction: Articaine in dentistry: an overview of the evidence and meta-analysis of the latest randomised controlled trials on articaine safety and efficacy compared to lidocaine for routine dental treatment. BDJ Open. 2021;7:29.

Reason for exclusion: Article is either an opinion paper, Editorial, Letter to the Editor, Response to published article or correspondence.

11. Schwendicke F, Tzschoppe M, Paris S. Corrigendum to “Radiographic caries detection: A systematic review and meta-analysis” [Journal of Dentistry 43 (2015) 924-933]. J Dent. 2021;114:103783.

Reason for exclusion: Article is either an opinion paper, Editorial, Letter to the Editor, Response to published article or correspondence.

12. Jayaraman J, Nagendrababu V, Pulikkotil SJ, Innes NP. Critical appraisal of methodological quality of Systematic Reviews and Meta-analysis in Paediatric Dentistry journals. Int J Paediatr Dent. 2018;28:548–60.

Reason for exclusion: Article is either an opinion paper, Editorial, Letter to the Editor, Response to published article or correspondence.

13. Schiegnitz E, Al-Nawas B, Kämmerer PW, Grötz KA. Erratum to: oral rehabilitation with dental implants in irradiated patients: a meta-analysis on implant survival. Clin Oral Investig. 2015;19:1693.

Reason for exclusion: Article is either an opinion paper, Editorial, Letter to the Editor, Response to published article or correspondence.

14. Xia QC, Feng ZX, Ping CX. Evaluating the efficacy of Tui Na in treatment of childhood anorexia: a meta-analysis. Altern Ther Health Med. 2014;20:45–52.

Reason for exclusion: Article is not related to dentistry.

15. Manfredini D, Greene CS, Ahlberg J, De Laat A, Lobbezoo F, Klasser GD. Evidence-based dentistry or meta-analysis illness? A commentary on current publishing trends in the field of temporomandibular disorders and bruxism. J Oral Rehabil. 2019;46:1–4.

Reason for exclusion: Article is either an opinion paper, Editorial, Letter to the Editor, Response to published article or correspondence.

16. Balevi B. In selected sites, short, rough-surfaced dental implants are as successful as long dental implants: a critical summary of Pommer B, Frantal S, Willer J, Posch M, Watzek G, Tepper G. Impact of dental implant length on early failure rates: a meta-analysis of observational studies. J Clin Periodontol 2011;38(9):856-863. J Am Dent Assoc. 2013;144:195–6.

Reason for exclusion: Article is either an opinion paper, Editorial, Letter to the Editor, Response to published article or correspondence.

17. Machmud PB, Djuwita R, Gayatri D, Khairani N, Putra WKY, Ronoatmodjo S. Influence of Micronutrient Consumption by Tuberculosis Patients on the Sputum Conversion Rate: A Systematic Review and Meta-analysis Study. Acta Med Indones. 2020;52:118–24.

Reason for exclusion: Article is not related to dentistry.

18. Moles DR, Needleman IG, Niederman R, Lau J. Introduction to cumulative meta-analysis in dentistry: lessons learned from undertaking a cumulative meta-analysis in periodontology. J Dent Res. 2005;84:345–9.

Reason for exclusion: Article is either an opinion paper, Editorial, Letter to the Editor, Response to published article or correspondence.

19. Tilliss T, Carey CM. Insufficient Evidence within a Systematic Review and Meta-Analysis of Powered Toothbrushes over Manual Toothbrushes for Soft Tissue Health During Orthodontic Treatment. J Evid Based Dent Pract. 2018;18:176–7.

Reason for exclusion: Article is either an opinion paper, Editorial, Letter to the Editor, Response to published article or correspondence.

20. Lee HY, Pyun JH, Shim SR, Kim JH. Medical Treatment for Peyronie’s Disease: Systematic Review and Network Bayesian Meta-Analysis. World J Mens Health. 2024;42:133–47.

Reason for exclusion: Article is not related to dentistry.

21. van ’t Hof MA. [Meta-analysis and “evidence-based practice” in dentistry]. Ned Tijdschr Tandheelkd. 2001;108:266–8.

Reason for exclusion: Article is either an opinion paper, Editorial, Letter to the Editor, Response to published article or correspondence.

22. Papageorgiou SN. Meta-analysis for orthodontists: Part I--How to choose effect measure and statistical model. J Orthod. 2014;41:317–26.

Reason for exclusion: Article is either an opinion paper, Editorial, Letter to the Editor, Response to published article or correspondence.

23. Papageorgiou SN. Meta-analysis for orthodontists: Part II--Is all that glitters gold? J Orthod. 2014;41:327–36.

Reason for exclusion: Article is either an opinion paper, Editorial, Letter to the Editor, Response to published article or correspondence.

24. Papadopoulos MA. Meta-analysis in evidence-based orthodontics. Orthod Craniofac Res. 2003;6:112–26.

Reason for exclusion: Article is not a review.

25. Cohen PA. Meta-analysis: application to clinical dentistry and dental education. J Dent Educ. 1992;56:172–5.

Reason for exclusion: No Abstract or Full Text available.

26. Yang S, Ying K, Wang F, Wang L, Ren X, Yang Q. [Methodological and reporting quality assessment for Chinese systematic reviews and meta analysis in oral medicine]. Shanghai Kou Qiang Yi Xue. 2015;24:505–10.

Reason for exclusion for exclusion: Article is not a review.

27. Kamaleldin M, Kilcommons S, Opgenorth D, Fiest K, Karvellas CJ, Kutsogiannis J, et al. Midodrine therapy for vasopressor dependent shock in the intensive care unit: a protocol for a systematic review and meta-analysis. BMJ Open. 2022;12:e064060.

Reason for exclusion: Article is not related to dentistry.

28. Roman MA, Abbasciano RG, Pathak S, Oo S, Yusoff S, Wozniak M, et al. Patient blood management interventions do not lead to important clinical benefits or cost-effectiveness for major surgery: a network meta-analysis. Br J Anaesth. 2021;126:149–56.

Reason for exclusion: Article is not related to dentistry.

29. Li C, Lü J, Su N, Li S, Shi Z. [Preferred reporting items for systematic reviews and meta-analysis for reporting quality of Chinese meta-analysis on stomatology]. Zhonghua Kou Qiang Yi Xue Za Zhi. 2011;46:257–62.

Reason for exclusion: Article is not a review.

30. Ravidà A, Wang I-C, Sammartino G, Barootchi S, Tattan M, Troiano G, et al. Prosthetic Rehabilitation of the Posterior Atrophic Maxilla, Short (≤6 mm) or Long (≥10 mm) Dental Implants? A Systematic Review, Meta-analysis, and Trial Sequential Analysis: Naples Consensus Report Working Group A. Implant Dent. 2019;28:590–602.

Reason for exclusion: Article is retracted.

31. Zhang Z, Xiao W, Jia J, Chen Y, Zong C, Zhao L, et al. Retraction notice to “The effect of combined application of pentoxifylline and vitamin E for the treatment of osteoradionecrosis of the jaws: A meta-analysis” [Oral Surgery, Oral Medicine, Oral Pathology and Oral Radiology 129/3 (2020) 207-214]. Oral Surg Oral Med Oral Pathol Oral Radiol. 2021;132:751.

Reason for exclusion: Article is retracted.

32. Cao YB, Zhang JD, Shen H, Jiang YY. Rivaroxaban versus enoxaparin for thromboprophylaxis after total hip or knee arthroplasty: a meta-analysis of randomized controlled trials. Eur J Clin Pharmacol. 2010;66:1099–108.

Reason for exclusion: Article is not related to dentistry.

33. Manini DR, Shega FD, Guo C, Wang Y. Role of Platelet-Rich Plasma in Spinal Fusion Surgery: Systematic Review and Meta-Analysis. Adv Orthop. 2020;2020:8361798.

Reason for exclusion: Article is not related to dentistry.

34. Sensever F de A, de Lucena Alves CP, Lima G da S, Loomans B, Opdam N, Pereira-Cenci T. Spin and reporting in systematic reviews with meta-analysis of randomized clinical trials in restorative dentistry. J Dent. 2022;125:104282.

Reason for exclusion: Article is not a review.

35. de Lucena Alves CP, Vetromilla BM, Moreno LB, Helal L, Sarkis-Onofre R, Pereira-Cenci T. Systematic reviews on the success of dental implants present low spin of information but may be better reported and interpreted: An overview of systematic reviews with meta-analysis. Clin Implant Dent Relat Res. 2022;24:105–15.

Reason for exclusion: Article is not a review.

36. The Editor recommends this issue’s article to the reader: Anaesthetic efficacy of Articaine versus Lidocaine in children’s dentistry: a systematic review and meta-analysis. Int J Paediatr Dent. 2018;28:346.

Reason for exclusion: Article is either an opinion paper, Editorial, Letter to the Editor, Response to published article or correspondence.

37. The Editor recommends this issue’s article to the reader: Critical appraisal of methodological quality of Systematic Reviews and Meta-analysis in Paediatric Dentistry journals. Int J Paediatr Dent. 2018;28:547.

Reason for exclusion: Article is either an opinion paper, Editorial, Letter to the Editor, Response to published article or correspondence.

38. Wei W, Liu R, ZhangTong Y, Qiu Z. The efficacy of specific neuromodulators on human refractory chronic cough: a systematic review and meta-analysis. J Thorac Dis. 2016;8:2942–51.

Reason for exclusion: Article is not related to dentistry.

39. Polychronopoulou A. The reporting quality of meta-analysis results of systematic review abstracts in periodontology and implant dentistry is suboptimal. J Evid Based Dent Pract. 2014;14:209–10.

Reason for exclusion: Article is not a review.

40. Zhang Y, Sun X, Li K, Wang X, Cai L, Li X, et al. “The Therapy of Elimination First” for Early Acute Mastitis: A Systematic Review and Meta-Analysis. Evid Based Complement Alternat Med. 2018;2018:8059256.

Reason for exclusion: Article is not related to dentistry.

41. Mittal N, Goyal M, Mittal PK. Understanding and Appraising Systematic Reviews and Meta-Analysis. J Clin Pediatr Dent. 2017;41:317–26.

Reason for exclusion: Article is not related to the topic.

42. Mendes V, Dos Santos GO, Moraschini V. WITHDRAWN: Interrelation of periodontal parameters between asthmatics and nonasthmatics subjects: a systematic review and meta-analysis. J Dent. 2018;69:32–40.

Reason for exclusion: Article is withdrawn.

**3.List of articles which have not been retrieved**

1. da Cunha BM, Wambier LM, da Rosa SV, Botelho-Filho CR, Rocha JS, Vettore MV, et al. Association between sense of coherence and oral clinical conditions in adults and the elderly: systematic review and meta-analysis. Community Dent Health. 2022;39:74–85.

**4.List of excluded articles after screening the full text and individual reasons for exclusion**

1. Wagner D, Lévy-Benichou H, Lefebvre F, Bolender Y. [Are self-ligating brackets more efficient than conventional brackets ? A meta-analysis of randomized controlled and split-mouth trials]. Orthod Fr. 2020;91:303–21.

Reason for exclusion: Article not in English.

2. Chen L, Chen C, Li Z-Y, Zhang Q. Clinical performance of intraoral digital impression for fixed prosthodontics: a Meta-analysis. Hua Xi Kou Qiang Yi Xue Za Zhi. 2021;39:306–12.

Reason for exclusion: Article not in English.
